# Local Environmental Effects on Light-Driven CO_2_ Reduction in Liposomes

**DOI:** 10.1021/acscatal.5c03610

**Published:** 2026-02-25

**Authors:** Amir Abbas, Richard Jacobi, Ingrid Merker, Riccarda Müller, Nathaniel R. Ritz, Nitish Kumar, Hani M. Elbeheiry, Dieter Sorsche, Kerstin Leopold, Leticia González, Andrea Pannwitz

**Affiliations:** † Institute of Inorganic Chemistry I, 9189 Ulm University, Albert-Einstein-Allee 11, Ulm 89081, Germany; ‡ Institute of Theoretical Chemistry, Faculty of Chemistry, 27258 University of Vienna, Währinger Straße 17, Vienna 1090, Austria; § Doctoral School in Chemistry (DoSChem), University of Vienna, Währinger Straße 42, Vienna 1090, Austria; ∥ Institute of Analytical and Bioanalytical Chemistry (IABC), Ulm University, Albert-Einstein-Allee 11, Ulm 89081, Germany; ⊥ Department of Chemistry, University of Michigan, Ann Arbor, Michigan 48109, United States; # Institute for Inorganic and Analytical Chemistry, 9378 Friedrich Schiller University Jena, Humboldtstraße 8, Jena 07743, Germany; ∇ Vienna Research Platform on Accelerating Photoreaction Discovery, University of Vienna, Währinger Straße 17, Vienna 1090, Austria; ○ Center for Energy and Environmental Chemistry Jena (CEEC Jena), Friedrich Schiller University Jena, Philosophenweg 7a, Jena 07743, Germany; ◆ Helmholtz Institute for Polymers in Energy Applications Jena (HIPOLE Jena), Lessingstraße 12−14, Jena 07743, Germany

**Keywords:** photocatalysis, liposomes, CO_2_ reduction, local environment, cobalt porphyrin

## Abstract

We report the governing principles that regulate the activity of light-driven CO_2_ reduction by a molecular photosensitizer bis­(2,2′-bipyridine)-(4,4′-dinonyl-2,2′-bipyridine)-ruthenium­(II) (**RuC**_**9**_) and a molecular catalyst (5,10,15,20-tetra­(4-methylphenyl)­porphinato)­cobalt­(II) (**CoTTP**) in supramolecular assembly within the lipid bilayers of liposomes suspended in water. We tested six different lipids with membranes in either the gel phase, fluid phase, or at the transition between both states, as well as zwitterionic or negatively charged headgroups. The correlation of the membrane rigidity with light-driven catalysis performance is not conclusive for the investigated set of lipid membranes, but molecular dynamics simulations elucidate how catalyst efficiency increases with the distance from the membrane center as well as their calculated vertical reduction energies. Luminescence quenching studies revealed that mainly dynamic quenching was observed with the highest quenching efficiency found with 1,2-dimyristoyl-*sn*-glycero-3-phosphocholine (DMPC) and 1,2-dipalmitoyl-*sn*-glycero-3-phospho-(1′-rac-glycerol)­(sodium salt) (DPPG)-based liposomes, in agreement with the results of the best performance in photocatalysis and the computational insights. A variation of cations did not show any significant influence on the performance, as opposed to electrochemical studies. The overall mechanistic findings of this study provide design principles for light-driven CO_2_ reduction by molecular components in liposomes.

## Graphical abstract

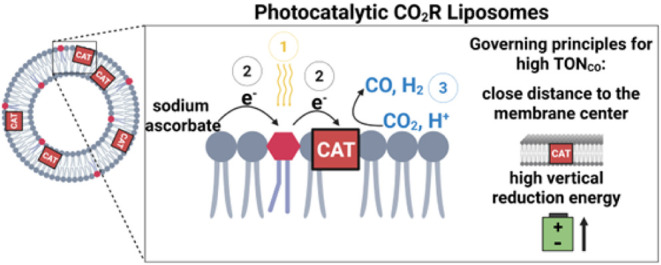

## Introduction

In the context of carbon capture, utilization, and storage, chemical conversion of CO_2_ to syngas, feedstock materials, and fuels is a challenging, but promising approach.^1–3^ In natural photosynthesis, light-driven CO_2_ conversion occurs in the chloroplasts, where light-absorbing molecular components are assembled within the thylakoid’s lipid bilayer. A variety of lipid bilayer-based systems were reported to perform light-driven processes such as CO_2_ reduction reactions, by embedding active units, ensuring solubility in water, and maintaining mild reaction conditions.^4–8^ The use of self-assembled lipid bilayers promotes a high local concentration of reactive components, accelerates charge separation, and suppresses side reactions such as charge recombination and photosensitizer degradation.^5,9,10^ In typical artificial photocatalytic and bioinspired systems, three components are present: a sacrificial electron donor, a photosensitizer (PS), and a catalyst (CAT). Frequently, second- and third-row transition metals (e.g., Ru, Ir, Re) are chosen for the PS and CAT, due to their (photo)­redox chemistry and stability, at the cost of side effects such as toxicity and low abundance.^11–15^

Herein we report on the light-driven CO_2_ reduction reaction at lipid bilayer membranes in water, using as embedding units the amphiphilic PS bis­(2,2′-bipyridine)-(4,4′-dinonyl-2,2′-bipyridine)-ruthenium­(II) (**RuC**_**9**_) and the (5,10,15,20-tetra­(4-methylphenyl)­porphinato)­cobalt­(II) (**CoTTP**) as CAT (Figure 1). The amphiphilic **RuC**_**9**_ is known for its electrostatic and hydrophobic interactions of its [Ru­(bpy)_3_]^2+^-type headgroup and alkyl tails at the bilayer–water interface of phospholipid membranes.^10,16–18^ Additionally, **RuC**_**9**_ retains its activity upon embedding within the membrane, as observed for various liposome-based systems, including for light-driven CO_2_ reduction.^6,10^ The cobalt porphyrin **CoTTP** is a hydrophobic, earth-abundant metal complex well known for its activity in catalyzing CO_2_ reduction.^19^ Other water-soluble cobalt porphyrins were reported to be active in light-driven CO_2_ reduction, using [Ru­(bpy)_3_]^2+^ as PS in an aqueous environment and in the presence of sodium ascorbate as a sacrificial electron donor under homogeneous conditions.^20^ Embedding a cobalt porphyrin with long alkyl tails along with **RuC**_**9**_ PS in lipid bilayers of liposomes proved to accelerate the light-induced charge separation and slow down charge recombination.^10^

**Figure 1:**
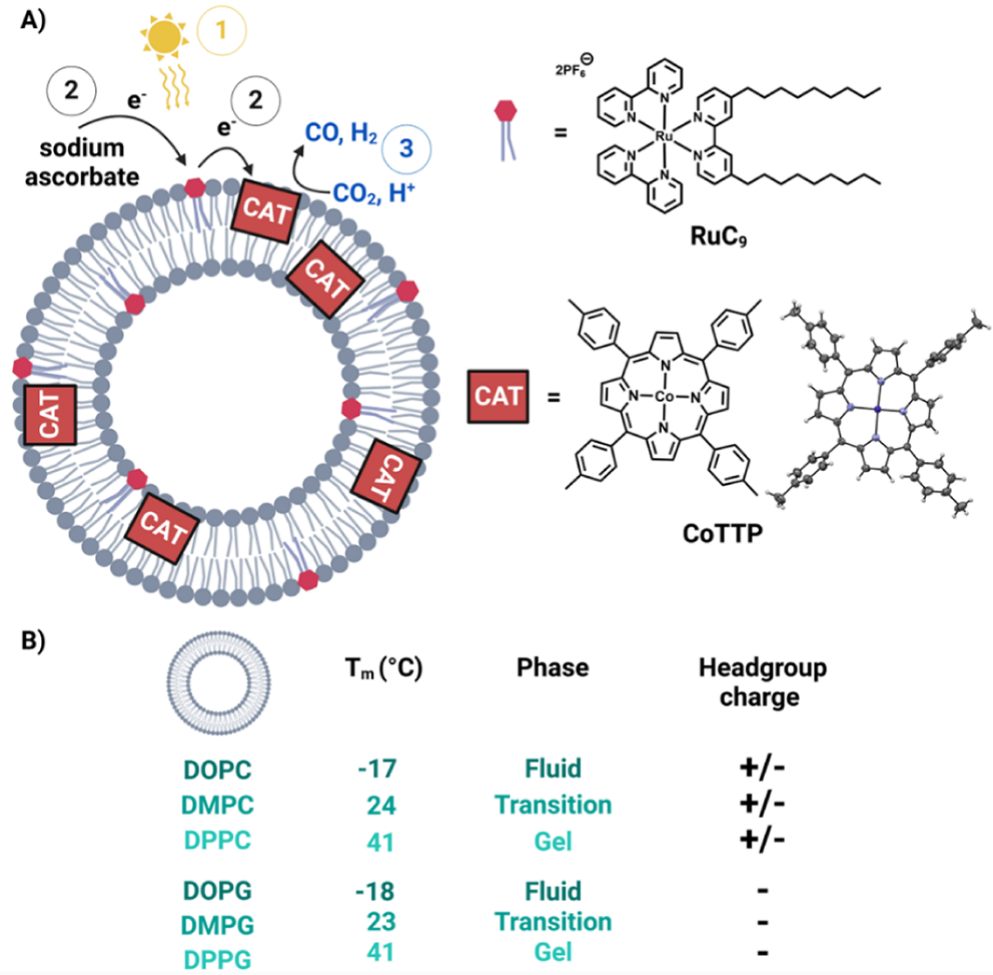
A) Overview of the light-active system for CO_2_ reduction including the lipid bilayer, structures of the photosensitizer **RuC_9_** and the catalyst **CoTTP**, and a schematic of electron transfers dynamics upon irradiation. Solid-state structure of **CoTTP**, CCDC identifier: 2394847. B) Scheme depicting the six lipids applied to produce liposome membranes, with their respective transition temperature (*T*_m_), aggregation phase, and headgroup charge. Created with permission from Biorender.com.

In the work presented here, we studied the governing design principles of the local lipid bilayer environment and its influence on light-driven CO_2_ reduction catalysis. For this purpose, we chose six lipids with zwitterionic and negatively charged headgroups and the respective transition temperatures (*T*_m_) at, below, and above room temperature (see Figure 1B). By varying the *T*_m_, positive and negative effects on diffusion mobility and local concentration within the membrane, as well as intermolecular interactions between components, have been reported in previous liposome-based systems.^4,9,21,22^ Using molecular dynamics simulations and theoretical, membrane-specific redox potential calculations of the catalyst in these six supramolecular assemblies, we elucidated the local solvation effect on the catalyst’s electronics and correlated it to the light-driven catalysis performance. As the cations play a crucial role in electrocatalytic CO_2_ reduction,^23,24^ we also varied the cations. Catalysis and electron transfer dynamics were elucidated in the presence of only Na^+^, K^+^, Li^+^, and Cs^+^ cations in the aqueous solution. CAT and PS uptake into the liposome samples was quantified by high-resolution continuum source graphite furnace atomic absorption spectrometry (HR-CS-GFAAS). The light-driven reduction products were determined via gas chromatography (GC). Molecular dynamics simulations and reduction energy computations yielded detailed information about the local environment around **CoTTP** and **RuC**_**9**_ in the different lipid bilayers, explaining the variations in the catalytic performance. The findings are complemented by *Stern–Volmer* quenching studies using steady-state and time-resolved emission spectroscopy, evaluating the initial electron transfer dynamics in correlation with the catalysis performance.

## Results and Discussion

### Synthesis and Liposome Preparation

The PS **RuC**_**9**_ ^17,25^ and the CAT (**CoTTP**)^26^ were synthesized as previously reported and described in the Supporting Information. A solid-state structure was obtained from a dark red single crystal grown via vapor diffusion of diethyl ether into an acetonitrile solution of **CoTTP**. The asymmetric unit contains two **CoTTP** complexes. Both structures show the cobalt­(II) center to be in a distorted square-planar coordination sphere defined by a twisted **TTP** ligand. Cocrystallized diethyl ether shows no interaction with the metal ion; however, there are short contact interactions on the axial positions of each cobalt center with the carbon–carbon bond of a pyrrolyl ring of neighboring **CoTTP** molecules oriented in a side-on fashion. These interactions range from 3.188 Å to 3.347 Å and have been observed in comparable structures.^27–32^ The tolyl substituents are twisted out of plane relative to the porphyrin core by angles ranging from roughly 50–84° which reduces steric repulsion between protons of the tolyl group and protons of the porphyrin backbone. A representative solid-state structure of **CoTTP** is shown in Figure 1, further details can be found in the Supporting Information (page 3), and the respective structural data have been deposited in the CCDC database with identifier 2394847.

For catalysis samples, a molar ratio of 10:1 for **RuC**_**9**_ **:CoTTP** was chosen. An excess of PS compared to CAT molecules is typically applied in light-driven catalysis with individual molecular PS and CAT molecules, especially when a CAT molecule needs to receive or release multiple electrons prior to catalytic activity (here: 2 electron accumulation for CO_2_ to CO reduction). This ratio also optimizes the performance of the CAT as an excess of PS surrounds the CAT and therefore improves electron transfer dynamics toward the CAT molecule.^4–6,9,10,18^

The following lipids with the transition temperature (*T*_m_) below, at, or above room temperature and negatively or neutrally charged headgroups were applied (see Figure 1): 1,2-dioleoyl-*sn*-glycero-3-phosphocholine (DOPC, fluid phase, neutral), 1,2-dimyristoyl-*sn*-glycero-3-phosphocholine (DMPC, transition phase, neutral), 1,2-dipalmitoyl-*sn*-glycero-3-phosphocholine (DPPC, gel phase, neutral), 1,2-dioleoyl-*sn*-glycero-3-phospho-(1′-rac-glycerol) (sodium salt) (DOPG, fluid phase, negative), 1,2-dimyristoyl-*sn*-glycero-3-phospho-(1′rac-glycerol) (sodium salt) (DMPG, transition phase, negative), 1,2-dipalmitoyl-*sn*-glycero-3-phospho-(1′-rac-glycerol) (sodium salt) (DPPG, gel phase, negative), and 1,2-dimyristoyl-*sn*-glycero-3-phosphoethanolamine-N-[methoxy­(polyethylene glycol)-2000] (14:0 PEG2000 PE) as a stabilizing agent which prevents aggregation via steric repulsion at the membrane surfaces.^33^ In a typical experiment, liposomes with embedded **RuC**_**9**_ and **CoTTP** components were prepared in a 100:1:2:0.2 molar ratio of the (main lipid):(14:0 PEG2000 PE):**RuC**_**9**_:**CoTTP** via thin-film hydration with a sodium bicarbonate aqueous solution (0.1 M), as described in the literature.^18^ Follow-up extrusion and purification via size exclusion chromatography yielded monodisperse liposomes. Size exclusion chromatography was performed to remove potential excess molecular PS and CAT that might not be embedded within the liposomes, ensuring that the measured catalytic activity is attributed to liposome-embedded PS and CAT only.

Liposome’s size distribution was characterized by dynamic light scattering, with a typical hydrodynamic diameter (Z_Avg_) in the range of 127–146 nm and 125–165 nm, before and after photocatalysis, in agreement with previous studies.^18^ Measurements of the zeta potential (ζ) indicated a neutral, negative, or positive surface charge of the liposomes which was in all cases in line with the molar ratio of the (main lipid):(14:0 PEG2000 PE):**RuC**_**9**_ applied (see Table S3). Additional measurements were performed by changing the cation (Na^+^ replaced with K^+^, Li^+^, or Cs^+^) in the bicarbonate aqueous solution (see details in Supporting Information, page 6). The liposome stability and size distribution were only partially affected before irradiation, with Z_Avg_ in the range of 121–190 nm, the only exception was for DPPG with Cs^+^ cation with Z_Avg_ = 70.0 ± 1.8 nm. After catalysis, in both DPPG- and DPPC-based liposomes (Li^+^) a great increase in size distribution was observed, with 517.4 ± 1.1 nm and 500.2 ± 4.3 nm, respectively (Table S4).

### Membrane Uptake

To quantify the resulting actual amounts of **RuC**_**9**_ and **CoTTP** in light-active liposome samples under the experimental conditions, high-resolution continuum source graphite furnace atomic absorption spectrometry was performed (see Tables 1 and S5–S6). HR-CS-GFAAS is an established method for direct, interference-free quantification of elements at trace levels (down to low μg/kg) in complex matrices with high precision.^34^ In the row of zwitterionic lipid membranes (DOPC, DMPC, and DPPC), **Ru** concentrations of 2774 ± 207, 662 ± 64, and 706 ± 47 μg L^–1^ and **Co** concentrations of 100 ± 3, 77 ± 1, and 58 ± 1 μg L^–1^ were measured, respectively. In the row of negatively charged lipid membranes (DOPG, DMPG, and DPPG), **Ru** mass concentrations of 2266 ± 48, 1094 ± 115, and 1079 ± 76 μg L^–1^ and **Co** mass concentrations of 94 ± 2, 78 ± 2, and 68 ± 1 μg L^–1^ were determined, respectively. For both metals’ concentrations, a decreasing trend with increasing *T*_m_ was observed. Within the same headgroup class lipids, the **Ru** uptake was similar for lipids at the transition phase and gel phase (DMPC vs DPPC, DMPG vs DPPG). Interestingly, in DPPC, which immobilized 16 ± 1 nmol **RuC**_**9**_, our system showed a lower immobilization efficiency in comparison with a similar system where Klein et al. performed light-driven CO_2_ reduction with DPPC-based liposomes (>75 nmol).^6^ These additional losses of **RuC**_**9**_ in our case might be caused by the additional size exclusion purification step and different buffer. Nevertheless, with the uptake quantification of PS and CAT, it was possible to avoid underestimation of TON_CO_ and TOF_CO_ results in photocatalytic experiments.^6^

**Table 1:** Overview of Mass Concentrations of Ruthenium and Cobalt (γ_Ru_ and γ_Co_) Determined by HR-CS-GFAAS and the Corresponding Actual Amounts of **RuC_9_** and **CoTTP** in Different Liposome Samples^a^

Host lipid	γ_Ru_ (μg L^–1^)	RuC_9_ (nmol)^b^	γ_Co_ (μg L^–1^)	CoTTP (nmol)^c^
DOPC	2774 ± 207	55 ± 4	100 ± 3	3.4 ± 0.1
DMPC	662 ± 64	13 ± 1	77 ± 1	2.61 ± 0.05
DPPC	706 ± 47	16 ± 1	58 ± 1	2.35 ± 0.04
DOPG	2266 ± 48	45 ± 1	94 ± 2	3.2 ± 0.1
DMPG	1094 ± 115	22 ± 2	78 ± 2	3.0 ± 1.0
DPPG	1079 ± 76	24 ± 2	68 ± 1	2.60 ± 0.06
DPPC^d^	3772	75	-	-

a Experimental conditions: liposome samples with a 100:1:2:0.2 molar ratio of (main lipid):(14:0 PEG2000 PE):**RuC_9_**:**CoTTP** with c­(main lipid) = 5 mM, based on theoretical values.

b 100 nmol expected from vesicle preparation.

c 10 nmol expected from vesicle preparation.

d From ref 6 Experimental conditions before extrusion: [DPPC] = 6.25 mM, [NaDSPE-PEG2K] = 62.5 μM, [**RuC**_**9**_] = 25 μM in 0.1 M NaH_2_PO_4_ buffer.

### UV–Vis Spectra of CoTTP in Various Lipid Bilayers

To elucidate the lipid bilayer’s influence on the **CoTTP** catalyst, UV–vis studies were performed. To this end, liposome samples with 1% **CoTTP** loading and in the absence of **RuC**_**9**_ were prepared to analyze spectroscopically the environmental effect on the catalyst (Table S7). In accordance with the literature and in the organic solvent dichloromethane,^35^ **CoTTP** has two distinct absorption bands. One is the typical intense Soret band, which absorbs at λ_max,1_ = 412 nm and is characterized by a transition into the second electronic excited state S_2_. The second is the Q-band, with an absorption maximum at λ_max,2_ = 529 nm and referring to the porphyrin’s π–π* transition from the v = 0 vibrational level of the S_0_ electronic ground state to the v = 0 and v = 1 vibrational levels of the first S_1_ excited state (see Figure S2A).^36^

The presence of only one Q-band in **CoTTP** instead of four Q-bands in metal-free porphyrin is typical for metal-coordinated porphyrins and confirms the successful metalation.^37^ Under inert conditions, all lipids yielded approximately the same absorption maximum of the Soret band at 412 ± 1 nm. A slight increase in absorbance intensity was observed for lipids in the gel phase (DPPC, DPPG), partially reflecting the uptake measured by HR-CS-GFAAS (Table 1). In the presence of air, additional Soret bands and Q-bands of the Co­(III) species^35^ appeared, and in some cases the original bands of the Co­(II) vanished completely. The spectra are shown in Figure S2–S3 and the spectral data of the Co­(II) and Co­(III) species are reported in Table S7.

### Light-Driven CO_2_ Reduction

The visible-light-driven CO_2_ reduction was performed by combining liposome samples with a sacrificial electron donor (sodium ascorbate, 0.1 M) and bubbling CO_2_ through the solution (Supporting Information, page 9), obtaining an average final pH of 6.9. The experiment was typically performed at room temperature using a 460 nm LED light source, in an open-source, modular, and 3D-printed photoreactor.^38^ The CO and H_2_ amounts were quantified by gas chromatography with a barrier ionization discharge detector (GC-BID). Photocatalysis performance was defined by the TON_CO_ and the selectivity parameters, applying the following equations: TON_CO_ = $\frac{n\left(\mathrm{CO}\right)}{n\left(\mathrm{CAT or PS}\right)}$ and CO selectivity in % = $\left(\frac{n\left(\mathrm{CO}\right)}{n\left(H_{2}\right)+n\left(\mathrm{CO}\right)}\cdot 100\right)$ , where n­(X) is the amount of the respective substance in moles, and the only products of this photocatalysis are CO and H_2_. Other products like methane, formaldehyde, methanol, formic acid, or products with higher carbons were not detected in either the^1^H NMR or the GC-MS measurements (Figures S9, S10, and S15–S19). TON_CO_ and selectivity were calculated with the actual uptake of **CoTTP** and **RuC**_**9**_. The TONs_CO_ and selectivities after 24 h of irradiation in different liposomes and in the absence of lipids are pictured in Figure 2A–C, respectively, which show a great dependence on the main lipid used for photocatalysis. Regarding the lower activity of the reference in the absence of lipids (TON_CO_ of 6 ± 3), it must be considered that the C_9_ chains of the **RuC**_**9**_ may be less soluble in aqueous media and not as active in an aqueous environment as when intercalated in the lipid membrane. Sakai et al. investigated a homogeneous CO_2_ reduction system using water-soluble Ru­(bpy)_3_^2+^ and CoTPPS. The latter is a negatively charged derivative of the applied **CoTTP** here. In aqueous solution and in the presence of sodium ascorbate as an electron donor, they obtained a maximum TON_CO_ of 926.^20^

**Figure 2:**
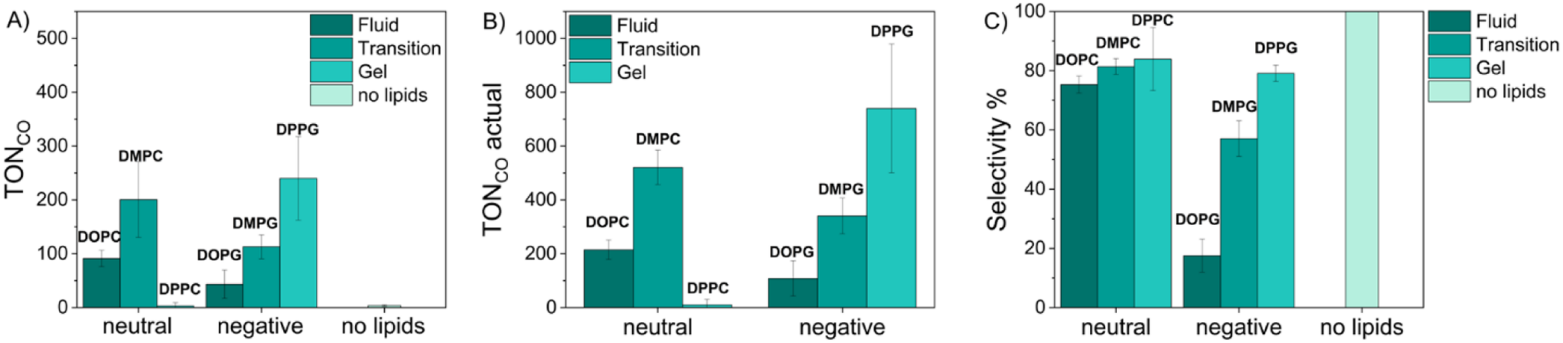
A) TON_CO_ results, after 24 h, of the light-active liposomes (neutral: DOPC, DMPC, DPPC; negative: DOPG, DMPG, DPPG) and the homogeneous solution of the equivalent amounts of **RuC_9_** and **CoTTP**, under irradiation of a 460 nm LED and in the presence of 0.1 M sodium ascorbate as a sacrificial electron donor. B) TON_CO_ calculated with the actual uptake of **CoTTP** determined by HR-CS-GFAAS. C) Corresponding selectivity (%) of the photocatalytic systems. Experimental conditions: liposome samples in a CO_2_ atmosphere with a composition of 100:1:2:0.2 of main lipid:(14:0 PEG2000 PE):**RuC_9_:CoTTP**, with c­(main lipid) = 0.3 mM and c­(sodium ascorbate) = 0.1 M. All values were acquired in triplicate with error bars representing the standard deviation.

In the series of zwitterionic lipids (DOPC, DMPC, and DPPC), TON_CO,DOPC_ (fluid phase) = 215 ± 42, TON_CO,DMPC_ (at transition phase) = 520 ± 63, and TON_CO,DPPC_ (gel phase) = 10 ± 6 were obtained (Table 2), showing a maximum TON_CO_ at the lipid’s transition phase with DMPC and a minimum TON _CO_ for the most rigid, gel phase system with DPPC. Moreover, the corresponding selectivity values were approximately the same within the entire series (≈80%).

**Table 2:** Overview of the Different Lipid Membranes’ Charge and *T*_m_, the Produced Amount of H_2_ and CO, the Theoretical (P)­TON_CO_ Based on Sample Preparation: 100:1:2:0.2 Molar Ratio of the (Main lipid):(14:0 PEG2000 PE):**RuC_9_**:**CoTTP** with c­(main Lipid) = 0.3 mM), the Actual (P)­TON_CO_, Selectivity (%), and Control Experiments without the CAT (100:1:2:0 Molar Ratio) or PS (100:1:0:0.2 Molar Ratio) as Indicated in the Column Note on Composition.^a^

Overall charge	*T*_m_ (°C)	Lipid	Note on composition	H_2_ (nmol)	CO (nmol)	TON_CO_ ^b^	TON_CO_ actual^c^	PTON_CO_ ^b^	PTON_CO_ actual^d^	Selectivity (%)
±	–17	DOPC		1.3 ± 0.4	4 ± 0.7	91 ± 15	215 ± 42	18 ± 3	27 ± 6	75 ± 3
±	24	DMPC		1.7 ± 0.3	7.5 ± 0.9	170 ± 21	521 ± 76	34 ± 4	207 ± 56	81 ± 3
±	41	DPPC		0.3 ± 0.3	1.3 ± 0.9	6 ± 3	10 ± 6	6 ± 4	30 ± 21	84 ± 11
-	–18	DOPG		9 ± 1	2 ± 1	43 ± 26	108 ± 68	9 ± 5	16 ± 10	17 ± 21
-	23	DMPG		4 ± 1	5 ± 0.9	113 ± 22	341 ± 72	23 ± 4	83 ± 24	57 ± 6
-	41	DPPG		3 ± 1	11 ± 3	240 ± 79	740 ± 256	48 ± 16	157 ± 20	79 ± 3
±	–17	DOPC	no CAT	0	0.3 ± 0.5	-	-	1 ± 2	1 ± 3	100
±	24	DMPC	no CAT	3 ± 2	2 ± 1	-	-	9 ± 7	28 ± 46	29 ± 15
±	41	DPPC	no CAT	0	0.3 ± 0.3	-	-	2 ± 2	4 ± 8	4 ± 8
-	–18	DOPG	no CAT	5 ± 5	1 ± 0.6	-	-	4 ± 3	4 ± 5	31 ± 27
-	23	DMPG	no CAT	4 ± 1	2.5 ± 0.2	-	-	11 ± 1	21 ± 9	21 ± 9
-	41	DPPG	no CAT	0	0.4 ± 0.3	-	-	2 ± 2	3 ± 5	100
±	–17	DOPC	no PS	0	0.5 ± 0.9	0.2 ± 0.4	0.5 ± 0.8	-	-	100
±	24	DMPC	no PS	0	0	0	0	-	-	-
±	41	DPPC	no PS	0	20 ± 20	8 ± 8	26 ± 27	-	-	100
-	–18	DOPG	no PS	78 ± 123	8 ± 3	3 ± 1	8 ± 3	-	-	46 ± 49
-	23	DMPG	no PS	0	0	0	0	-	-	-
-	41	DPPG	no PS	0	0	0	0	-	-	-

a All values were acquired after 24 h in triplicate, with error representing the standard deviation.

b Based on sample preparation.

c Based on actual **CoTTP** amount.

d Based on actual **RuC**_**9**_ amount.

The time trace of catalytic activity for the DMPC-based system is shown in Figure 3 and exhibits an increase in TON_CO_ in the first 4 h, then reaches a plateau, followed by a small decrease after 24 h, which might be due to the diffusion of gas through the vial. The selectivity increased within the first few hours, reaching a plateau at 80% after 2 h. These results are in agreement with the similar work by Reisner et al., who obtained the same time trace and TON_CO_ for their CO_2_ reduction system based on a **CoTTP** catalyst with C_17_ chains and a RuC_17_ photosensitizer on DMPC (at the transition phase).^10^

**Figure 3:**
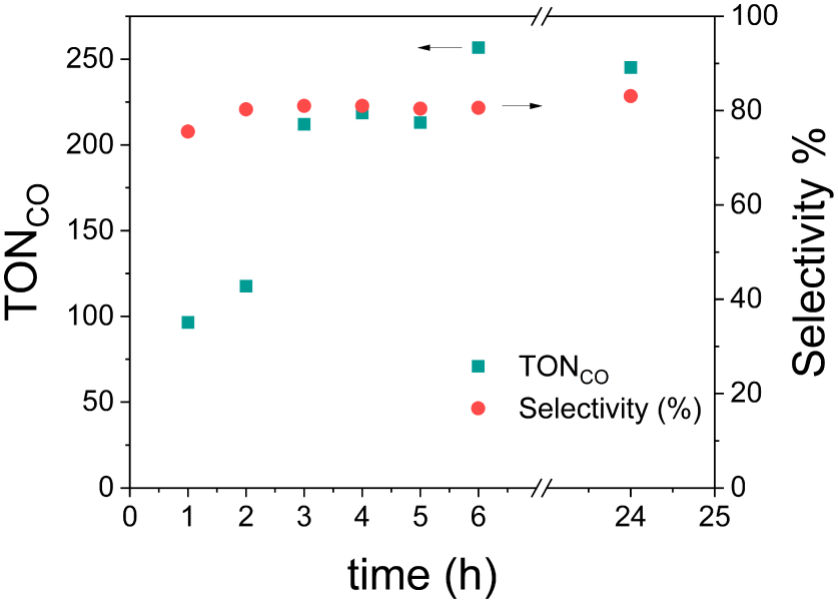
Time-dependent CO_2_ reduction activity: TON_CO_ and selectivity (%) results of the light-active DMPC vesicles, under irradiation of a 460 nm LED and monitored over 24 h. Experimental conditions: liposome samples in a CO_2_ atmosphere with a composition of 100:1:2:0.2 of main lipid:(14:0 PEG2000 PE):**RuC_9_:CoTTP**, with c­(main lipid) = 0.3 mM and c­(sodium ascorbate) = 0.1 M.

Additionally, the influence of **RuC**_**9**_:**CoTTP** ratio was tested by reducing the **CoTTP** amount in a ratio of 100:1:2:0.02 of the (main lipid):(14:0 PEG2000 PE):**RuC**_**9**_:**CoTTP**. A TON_CO_ of 889 ± 334 was observed, which is 4.5 times higher than the typical ratio applied in our experiments (Figure S6). Interestingly, the selectivity values were not affected by this modification. These results suggest that a higher ratio of **RuC**_**9**_:**CoTTP** could lead to an increase in the chance of electron transfers toward the catalyst, similar to previous studies on H_2_ evolution with DMPC liposomes and **RuC**_**9**_ as photosensitizer.^18^

In the series of negatively charged lipid membranes (DOPG, DMPG, and DPPG), TON_CO,DOPG_ (fluid phase) = 108 ± 65, TON_CO,DMPG_ (at transition phase) = 340 ± 66, and TON_CO,DPPG_ (gel phase) = 740 ± 240 were obtained (Table 2), observing an increasing catalytic activity with higher transition temperature and higher membrane rigidity of the main lipid phase. The same trend was observed for the corresponding selectivity values, where the selectivity is maximal and around 80% for the most rigid lipid bilayer, DPPG, and minimal for the most fluid lipid bilayer, DOPG, within the series of investigated lipids. This difference might be due to the electrostatic attraction of cations, such as H^+^ and Na^+^ from the buffer to the negatively charged membrane surface,^16,39^ which might influence photocatalytic CO_2_ reduction activity and the ratio of H_2_ to CO.

It is well known from literature how the presence of different cations play a crucial role in CO_2_ electrochemical reduction.^23,24^ Therefore, additional experiments were conducted to investigate the influence of various monovalent cations on photocatalysis (Na^+^ in the aqueous bicarbonate and ascorbate solution was replaced by K^+^, Li^+^, or Cs^+^ respectively, see details in Supporting Information, page 10). For this purpose, lipids with similar rigidity but different headgroup charges were chosen: the negatively charged DPPG and the zwitterionic DPPC. No particular trends in cation influence were observed for TON_CO_ and selectivity for both DPPG- and DPPC-based systems, with the exception of Li^+^, which induced a slight decrease, with TON_CO,DPPG,Li+_ = 170 ± 11 and TON_CO,DPPC,Li+_ = 7 ± 2, lower than the average value of TON_CO,DPPG,Na+_ = 245 ± 55 and TON_CO,DPPC,Na+_ = 24 ± 5 (Figure S7–8A,B). This might be explained by Li^+^ binding to the phospholipid headgroups which is stronger for Li^+^ than for other cations such as K^+^ and significantly stiffens the membrane.^40^ It might also interact more strongly with the **RuC**_**9**_ PS and thereby influence electron transfer dynamics. Regarding selectivity, a minor impact was observed in all cases, with an average selectivity_DPPG_ = 78% and selectivity_DPPC_ = 80%, basically in line with the original experiments (Table 2). These results lead to the conclusion that the choice of cations has a minor influence on light-driven CO_2_ reduction in the photocatalysis process for the larger cations Na^+^, K^+^, and Cs^+^, suggesting that—opposed to electrocatalysis—the transition state in catalysis is mostly cation size independent. Additionally, the fact that only the TON, but not the selectivity, is affected by Li^+^ might indicate that the H^+^ accessibility across the membrane is not influenced by the presence of different cations including Li^+^.

A final remark on this photocatalysis investigation is the confirmation of the source of CO produced. GC-MS analysis was carried out on the best-performing systems (DMPC and DPPG) where samples were bubbled with ^12^CO_2_ or ^13^CO_2_ in the presence of nonlabeled buffer or ^13^C-labeled buffer. Samples were then irradiated and submitted to GC-MS, and the ratio of the abundance of the ion *m*/*z* = 29 (^13^CO) to the ion *m*/*z* = 28 was recorded for all samples. An enhancement in this ratio relative to the blank experiment (^12^CO_2_ and nonlabeled buffer) was observed only for all the samples that were bubbled with ^13^CO_2_ (Figures S11–S14 and Table S8). Samples with labeled buffer but bubbled with regular ^12^CO_2_ did not show any improvement in the ^13^CO signal. All in all, the increase in the ^13^CO signal with only ^13^CO_2_ bubbling confirms that the source of CO is CO_2_.

### Computational Investigation of CoTTP in the Membranes

To rationalize the different catalytic performances of the different liposomes, we carried out classical molecular dynamics simulations on the neutral and negatively charged liposome systems. For the sake of simplicity and to single out key properties influencing the catalytic efficiency, only **CoTTP** was placed in a lipid bilayer membrane consisting of the respective lipid. This mimics the experimental sample preparation (see above and Supporting Information, page 5) where **RuC9** and **CoTTP** are combined for self-assembly prior to lipid film hydration. Since the diameter of the liposomes (around 150 nm) is significantly larger than the resulting simulation box (below 10 nm), we ignore the curvature of the lipids and approximate the lipid bilayer as a flat, two-dimensional membrane. Initially, **CoTTP** was placed upright in one of the membrane leaflets. The generation of the initial system was performed using the input generator CHARMM-GUI^41,42^ (see Supporting Information, page 19). The resulting systems were minimized, equilibrated, and simulated for 1.4 μs at 300 K with the program package AMBER22 ^43^ using established simulation conditions.^25^ We investigated a plethora of both geometrical and electrostatic properties of **CoTTP** in the different membranes, which can be found in detail in the Supporting Information, pages 19–23. In the following, we focus on the properties that uniquely distinguish each membrane and explain the differences in catalytic performance.

**CoTTP** remains embedded in the lipid bilayer membrane during all simulations, confirming its affinity for the hydrophobic lipid bilayer rather than transitioning to the aqueous phase. The density profiles of the Co metal center in the specific membranes are shown in Figure 4A. These density profiles are to be read as a cross-section normal (perpendicular) to the membrane surface. The left-hand side of the plots at distance 0 represents the center of the membrane, and at a relative distance of 1 is the interface to the aqueous bulk, where the headgroup density is maximum. Since the membranes are of different widths, all distances are scaled by half of the membrane width such that the center of the membrane is at 0 and the water interface is at 1. This representation allows us to compare both the distance to the center of the membrane as well as the distance to the surface of the membrane between the different lipids. Unscaled density profiles can be found in the Supporting Information, Figure S20. It is not purposeful to compare the negatively charged liposomes to the neutral ones, so as done above, the analysis is split into two for the two different charge states of the membrane. This split is justified as the surface charge of the lipids most definitely affects key parameters for the catalysis. These parameters include the diffusion and binding of reactants and products, as well as the electron transfer dynamics between the catalyst and the photosensitizer, or between the photosensitizer and the sacrificial electron donor. To reduce the impact of these headgroup-induced effects, we focus on trends between the three different phospholipids in each of the two charge states rather than comparing between the two.

**Figure 4:**
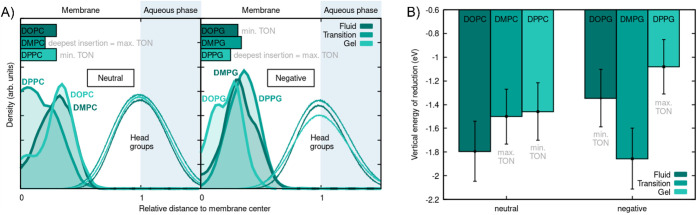
A) Computed density profiles of the Co metal center in the neutral (left) or negatively charged membranes (right) shown as shaded histograms. Headgroup densities are shown to indicate the interface between the membrane and the aqueous bulk solution. For improved visibility, the density profiles of Co are amplified by a factor of 5000. The average insertion depths of Co in each lipid are shown by bars at the top of the panels. B) Computed average vertical energy reduction for the six lipids. The error bars show the standard error. For reference, the liposomes that show the maximum and minimum TON experimentally are indicated next to the bars in both subfigures.

Both for the zwitterionic (the PCs) and negatively charged lipid bilayers (the PGs), the Co atom is located closer to the membrane center in one of the lipids compared to the respective other two. Interestingly, these two lipids do not have comparable transition temperatures *T*_m_. Rather, the lipid in which Co is located closest to the membrane center is, in the zwitterionic case, the lipid that has its transition phase at room temperature (DMPC), while for the negatively charged liposomes it is the lipid that is in its gel phase at room temperature (DPPG). Coincidentally, for these two lipids, the highest TONs were observed experimentally. This match between the largest insertion depth and the highest TONs in catalysis indicates that the center of the membrane represents the most favorable environment for catalysis, while the catalytic efficiency is reduced when the catalyst is closer to the charges of the negative or zwitterionic headgroups.

The two remaining systems (DOPC and DPPC or DOPG and DMPG, respectively) exhibit similar density profiles of cobalt in both the zwitterionic and negative cases. In these cases, **CoTTP** is clearly localized closer to the membrane–water interface rather than at the center of the membrane. While there are minor differences in the profiles and the resulting average insertion depths, these do not explain the differences in the catalytic performance for these four lipids. For instance, the insertion depths for DOPC and DPPC are identical; however, the DOPC liposomes show decent catalytic activity, with TON_CO,DOPC_ (fluid phase) = 215 ± 42, while the DPPC liposomes do not, with TON_CO,DPPC_ (gel phase) = 10 ± 6. Also, while DOPG shows slightly increased densities at the membrane center compared to DMPG, it actually exhibits a lower catalytic performance, with TON_CO,DOPG_ (*T*_m_ < rt) = 108 ± 65 and TON_CO,DMPG_ (at transition phase) = 340 ± 66.

Given the complexity of the systems, it is unlikely that a single property alone (such as the insertion depth) can fully explain the differences in catalytic performance. Accordingly, in addition to the insertion depths, we computed the vertical reduction energies of **CoTTP** in the different lipid bilayers using the B3LYP^44,45^ functional with the def2-SVP basis set,^46,47^ as implemented in the Gaussian 16 program package^48^ (details in the Supporting Information, page 19). We perform two single-point calculations, each on 100 snapshots sampled at equal temporal intervals for each of the six systems. We use a quantum mechanical/molecular mechanics (QM/MM) hybrid approach where we place **CoTTP** in the quantum mechanical region and represent the environment as point charges using electrostatic embedding. In the first computation, we set the total charge of **CoTTP** to zero, representing the doublet electronic state of Co­(II) before electron transfer; in the second computation, we set the charge to −1, which corresponds to the closed-shell electronic state after electron transfer. Low-spin configurations were used in both cases as they are preferred in the d^7_‑_^Co­(II) and d^8^-Co­(I) cases within the square planar ligand field induced by the porphyrin.^49^ The energy difference between the two calculations corresponds to the energy released upon binding of a free electron. The resulting values should not be mistakenly taken as redox potentials, as we do not explicitly include the electron donor and structural relaxation. However, assuming the electron donor is the same across all six systems and that structural rearrangements occur on a comparable scale in all of them, the relative energy differences between the systems are expected to qualitatively reflect trends in redox potentials. This approach involves a fraction of the computational cost that would be required for full electron transfer computations, such as those based on Marcus theory.^50^ In the framework described here, more negative vertical energies of reduction thus represent energetically more favorable reductions.

When comparing the DOPC- and DPPC-based liposomes (see Figure 4B), **CoTTP** in DOPC (−1.79 ± 0.25 eV) has a significantly lower (i.e., more negative) vertical reduction potential than DPPC (−1.46 ± 0.24 eV). This explains why, at similar insertion depths of the Co metal center, DOPC yields much larger TONs (TON_CO,DOPC_ (fluid phase) = 215 ± 42) than DPPC (TON_CO,DPPC_ (gel phase) = 10 ± 6). Analogously, **CoTTP** is embedded slightly less deeply in DMPG compared to DOPG, and it exhibits a significantly more negative vertical reduction potential in DMPG (−1.86 ± 0.26 eV), resulting in increased TONs (TON_CO,DMPG_ (at transition phase) = 340 ± 66) compared to DOPG (−1.35 ± 0.24 eV, TON_CO,DOPG_ (fluid phase) = 108 ± 65). Although it is gratifying to see that the so-calculated vertical reduction energies align with the different TONs, they alone do not explain catalytic performances, as the liposomes with the highest TONs, both neutral (DMPC) and negative (DPPG), do not actually exhibit the lowest reduction energies. Only the combination of vertical reduction energies and insertion depths can explain the different catalytic efficiencies. Based on the data in Figure 4, the following hypothesis can be formulated (see Figure 5): In general, the center of the membrane exhibits better conditions for photocatalysis than the regions closer to the headgroups. If the density of the catalyst is high at the center of the membrane, then high TONs can be achieved even at less-than-optimal reduction energies. However, if the insertion depths are equal to or similar between two systems, the vertical energy of reduction determines which system performs better under photocatalytic conditions.

**Figure 5:**
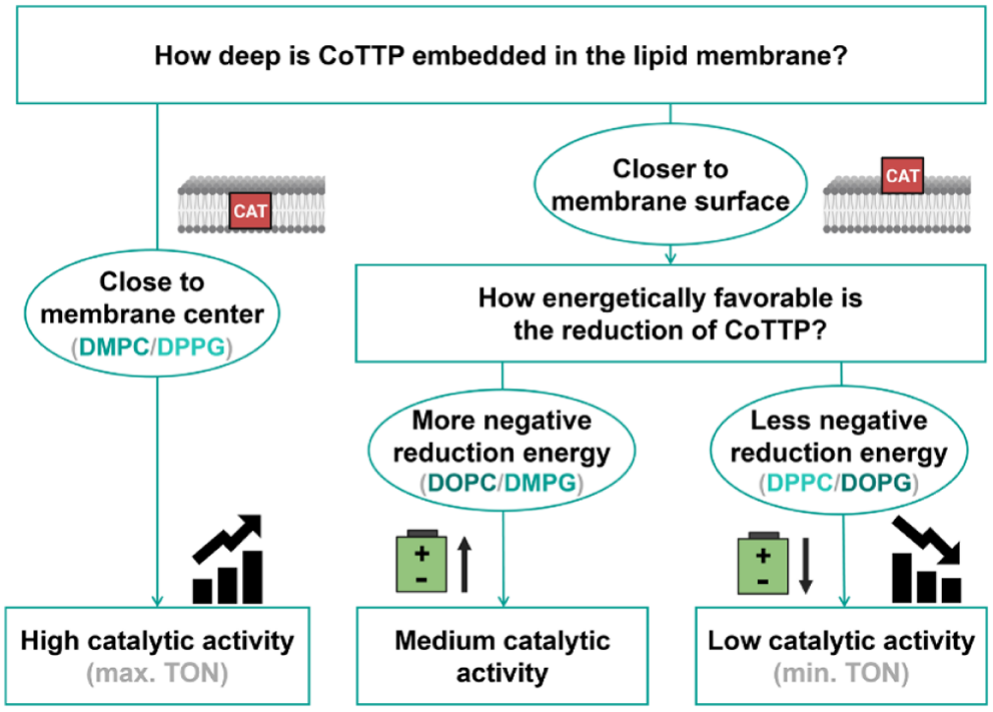
Decision tree representing the hypothesis on how the different liposome environments affect the catalytic performances of **CoTTP**.

### Excited-State Electron-Transfer Dynamics

In one of our previous studies on light-driven H_2_ evolution, with **RuC**_**9**_ and [Mo_3_S_13_]^2–^ as catalysts in liposomes, we had identified that the initial charge transfer upon photoirradiation determined the photocatalysis performances in different lipid bilayers.^18^ Thus, we investigated the initial electron transfer between the excited state of **RuC**_**9**_ and the electron donor ascorbate and catalyst in Stern–Volmer experiments by steady-state luminescence and time-resolved spectroscopy. For this purpose, the Stern–Volmer equation (eq 1) was used to calculate the Stern–Volmer (*K*_SV_) constants and quenching constants (*k*_q_).

1 $$\frac{I_{0}}{I}{=1+K}_{\mathrm{sv}}\left[Q\right]{=1+k}_{q}\tau_{0}\left[Q\right]$$

Experimentally, it was observed that the excited-state lifetime (τ_0_) of **RuC**_**9**_ was between 470 and 590 ns (see Table S9). In almost all lipids, the lifetime and emission intensity decreased with increasing quencher concentration (see Figure 6 and Supporting Information, pages 23–29), which indicates that emission quenching takes place with a dynamic-based quenching mechanism. Dynamic quenching is a diffusion-based process that occurs upon diffusion-controlled collisions or electron transfer between the quencher and the PS in the excited state.^36^ Interestingly, previous work has observed mainly static quenching as the common excited-state electron transfer mechanism involving the positively charged **RuC**_**9**_ within the lipid membrane and a negatively charged quencher prior to photoexcitation.^10,18,51^ However, the work presented here showed mainly dynamic, diffusion-based quenching which might be due to the fact that the previous works all operated in phosphate buffers and we applied bicarbonate buffer in the presence of CO_2_. Regarding quenching efficiency, DMPC (neutral, at transition phase, *k*_q_ = (4.0 ± 0.8) · 10^7^ L mol^–1^ s^–1^) is one order of magnitude more effective than DOPC (neutral, fluid phase, *k*_q_ = (6.0 ± 2.0) · 10^6^ L mol^–1^ s^–1^), DPPC (neutral, gel phase, *k*_q_ = (6.8 ± 2.6) · 10^6^ L mol^–1^ s^–1^), and DPPG (negative, gel phase, *k*_q_ = (6.7 ± 1.4) · 10^6^ L mol^–1^ s^–1^). In the case of DOPG (negative, fluid phase, *k*_q_ = (3.0 ± 0.7) · 10^6^ L mol^–1^ s^–1^) and DMPG (negative, at transition phase, *k*_q_ = (1.5 ± 0.6) · 10^6^ L mol^–1^ s^–1^), lower quenching was observed (Table S9), which is in line with the literature where electrostatic repulsion negatively affects the initial electron transfer.^18^ Considering the overall quenching efficiencies, the following series was observed: neutral lipids: DMPC ≫ DPPC > DOPC; negatively charged lipids: DPPG > DOPG ≫ DMPG. The trend in the quenching dynamics by ascorbate reflects only partially the trend in photocatalytic performances (Figure 7A).

**Figure 6:**
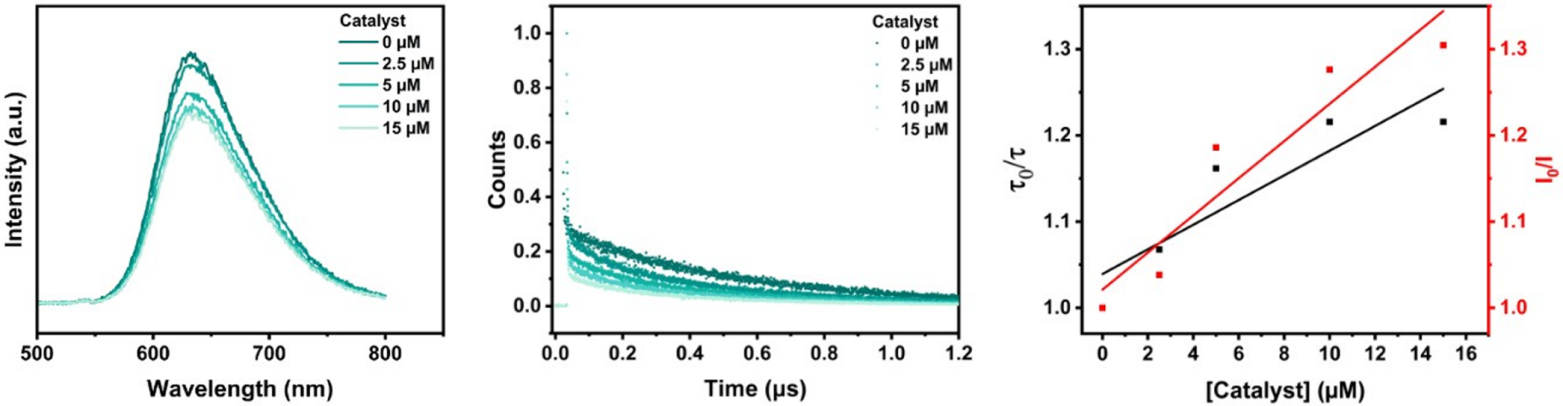
Photoinduced charge transfer in liposome samples, where *I*_0_ and *τ*_0_ are the values in the absence of quencher (sodium ascorbate and catalyst). Emission intensity, normalized lifetime measurements, and Stern–Volmer plots as a function of quencher concentration: Experimental conditions: DMPG liposome samples, with a composition of 100:1:2:X of main lipid:(14:0 PEG2000 PE):**RuC_9_**:**CoTTP** (main lipid = 0.3 mM), prepared in CO_2_ *-*saturated 0.1 M bicarbonate solution and in the presence of sodium ascorbate as a sacrificial electron donor (0.1 M).

**Figure 7:**
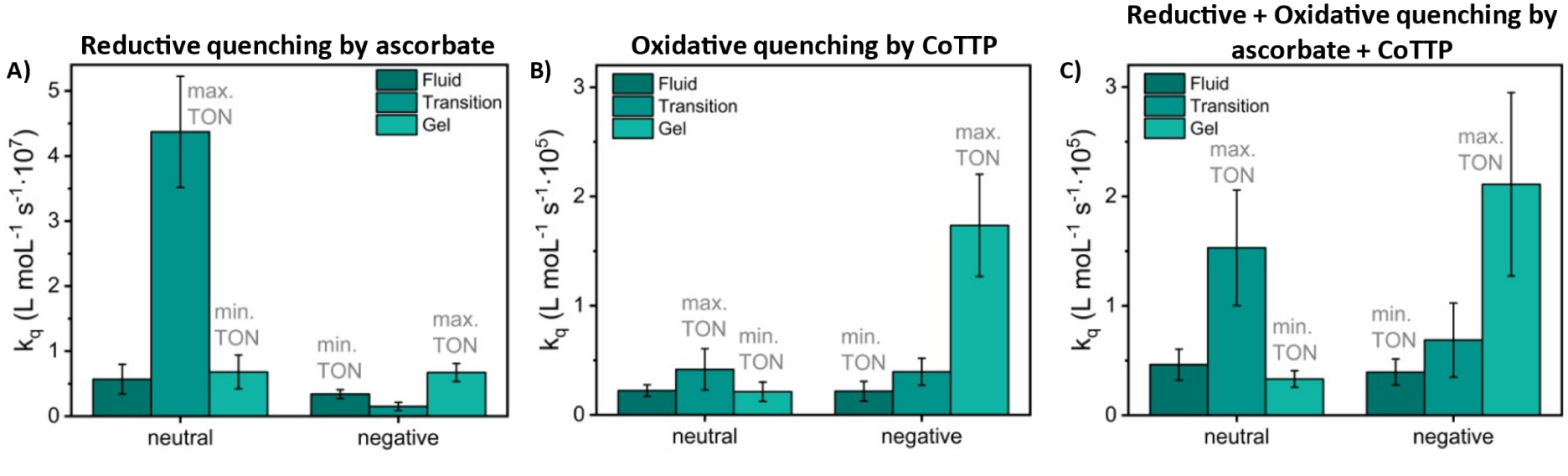
Rate constants comparison, based on emission intensity: A) Quenching constant *k*_q_ by sodium ascorbate as quencher. B) Quenching constant *k*_q_ by catalyst in the absence of a sacrificial electron donor. C) Quenching constant *k*_q_ under catalytic conditions. Experimental conditions: neutral (DOPC, DMPC, and DPPC) and negative (DOPG, DMPG, and DPPG) liposome samples with a composition of 100:1:2 of main lipid:(14:0 PEG2000 PE):**RuC_9_** or 100:1:2:X of main lipid:(14:0 PEG2000 PE):**RuC_9_**:**CoTTP**, at various loadings of **CoTTP**, with c­(main lipid) = 0.3 mM. Samples were prepared in CO_2_ *-*saturated 0.1 M bicarbonate solution. Values are reported as mean values ± error given by the respective fit function in the Stern–Volmer plots and the error of the lifetime as described in the Supporting Information.

In order to better correlate these findings with the photocatalysis results and the insights from molecular dynamic simulations, additional experiments, with liposome samples containing a constant amount of **RuC**_**9**_ at various loadings of catalyst as quencher, were performed. Dynamic-based quenching was observed in almost all cases (Table S9) with the following quenching efficiency: DMPC > DOPC > DPPC for neutral lipids and DPPG > DMPG > DOPG for negatively charged lipids. Interestingly, most samples showed efficiency two orders of magnitude lower, in comparison to cases where the electron donor was the quencher with DOPC (neutral, fluid phase, *k*_q_ = (2.2 ± 0.5) · 10^4^ L mol^–1^ s^–1^), DMPC (neutral, at transition phase, *k*_q_ = (4.2 ± 2.0) · 10^4^ L mol^–1^ s^–1^), DPPC (neutral, gel phase, *k*_q_ = (2.0 ± 0.9) · 10^4^ L mol^–1^ s^–1^), DOPG (negative, fluid phase, *k*_q_ = (2.0 ± 0.9) · 10^4^ L mol^–1^ s^–1^), and DMPG (negative, at transition phase, *k*_q_ = (4.0 ± 1.0) · 10^4^ L mol^–1^ s^–1^), respectively (Figure 7B). Notably, DPPG (negative, gel phase, *k*_q_ = (2.0 ± 0.5) · 10^5^ L mol^–1^ s^–1^) showed the highest efficiency and was the only case where static-based quenching was observed.

The competition between oxidative and reductive quenching was evaluated by testing various loadings of the catalyst in the presence of a fixed sodium ascorbate concentration as a sacrificial electron donor (0.1 M). The quenching dynamics showed similar values of *K*_sv_ and *k*_q_ and the same trends as observed in the quenching experiments where no reductive quencher was present (Figure 7C vs B). Additionally, an increase was observed for the DMPC-based sample, with *k*_q_ = (1.5 ± 1.0) · 10^5^ L mol^–1^ s^–1^ being three times higher than in the experiments conducted in the absence of an electron donor. Interestingly, the quenching in the series with combined quenching by the electron donor and **CoTTP** is most effective in DMPC- and DPPG-based liposomes. These lipids also provide the local environment for the most active photocatalysis (Figure 2) and the cases in which the catalyst is inserted into the membrane core (Figure 4A). For the cases with medium (DOPC, DMPG) and poor (DPPC, DOPG) photocatalytic activity, the trend is also reflected by the quenching in the presence of ascorbate and **CoTTP** (Figure 7C). However, the absolute values do not correlate, and the vertical reduction energy simulations (Figure 4B) are a better match for explaining the light-driven catalysis activity (Figures 8A,B and S25).

**Figure 8:**
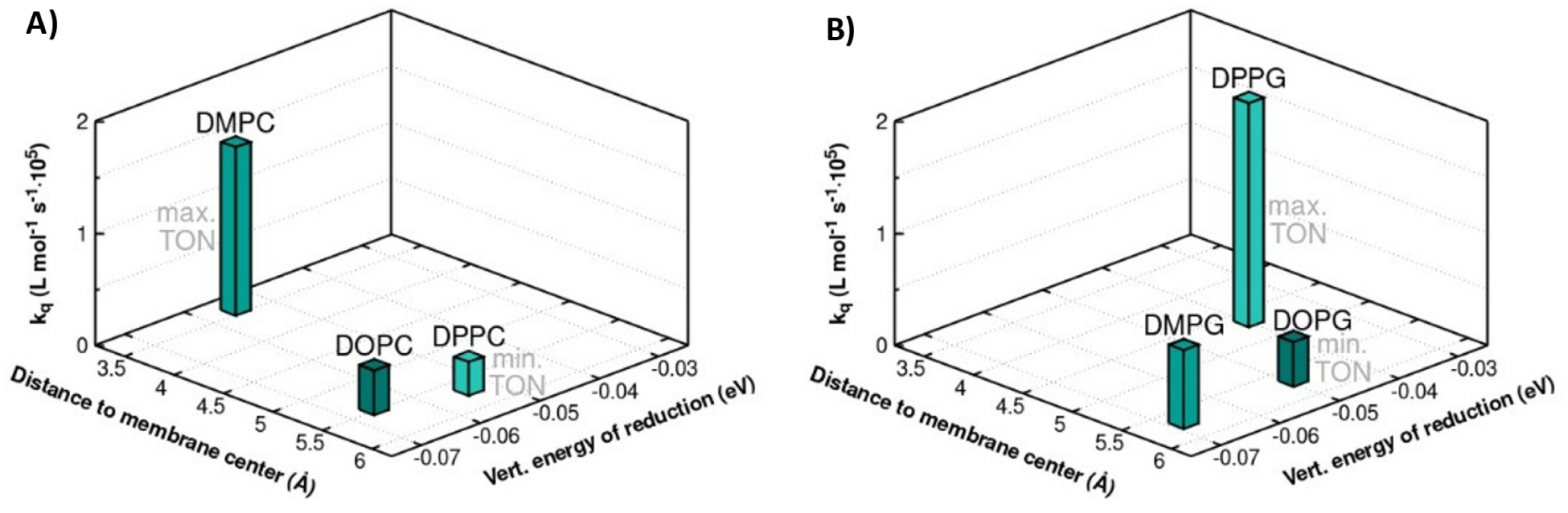
3D diagrams depicting: A) and B) Quenching constants (*k*_q_) related to the combined charge transfer between **RuC_9_** and **CoTTP** in the presence of sodium ascorbate in neutral (DOPC, DMPC, and DPPC) and negative (DOPG, DMPG, and DPPG) liposome samples under catalytic conditions, related to the computed Co metal center distance to the lipid membrane center and the computed vertical energy reduction.

It is concluded here that the deep membrane insertion governs the productive light-driven electron transfer. The deep insertion of **CoTTP** in the membrane core also correlates with a longer distance between **CoTTP** and **RuC**_**9**_. The latter is situated closer to the membrane surface due to its 2-fold positive charge. According to Marcus’ theory and due to a distance-correlated reorganization energy, electron transfer can become fastest at an ideal distance between the electron donor and acceptor.^52,53^ This distance-dependent effect on electron transfer dynamics might also play a dominant role in the supramolecular assembly of **CoTTP** and **RuC**_**9**_ reported here within lipid bilayers.

## Conclusions

Biomimetic lipid bilayers were functionalized with the molecular photosensitizer **RuC**_**9**_ and the catalyst **CoTTP**, forming photocatalytically active liposomes in water for CO_2_ reduction. In the two series of zwitterionic and negatively charged lipid membranes, the highest TON_CO_ values were observed for DMPC- and DPPG-based liposomes with TON_CO,DPPG_ (negative, gel phase) = 740 ± 240 and TON_CO,DMPC_ (neutral, at transition phase) = 520 ± 63, respectively. A variation of cations did not show significant influence on performance, as opposed to electrochemical studies. Luminescence quenching studies reveal the electron transfer dynamics of the initial charge transfer to the electron donor and **CoTTP** catalyst. The quenching dynamics show the same trends as the catalytic performance. It was found that the rigidity of the membrane cannot be used to predict catalytic activity. Instead, it was found that the governing design principle determining catalytic activity is a deep insertion of the **CoTTP** catalyst into the lipid bilayer toward the hydrophobic core as achieved by DMPC and DPPG. When the **CoTTP** catalyst is located closer to the membrane–water interface and the position of the **RuC**_**9**_ PS, the vertical reduction energy of the catalyst governs the catalytic performance with a more negative reduction energy of the catalyst being superior. These parameters were determined by molecular dynamics simulations and hybrid QM/MM calculations. The overall study explains the strong, medium, or poor photocatalytic activity of **CoTTP** in different local environments of supramolecular liposome-based systems. It also provides design guidelines and key parameters that are relevant for advancing light-driven CO_2_ reduction in various soft-matter architectures or compartmentalized conditions—an essential step toward scalable future technologies.

## Experimental Section

All experimental information, computational methods, and supplementary results of this study are reported in the Supporting Information.

## Supplementary Material





## Notes

Materials and techniques, synthesis and characterization (including the synthesis of (5,10,15,20-tetra­(4-methylphenyl)­porphinato)­cobalt­(II) (CoTTP), preparation of stock solutions, vesicle preparation, dynamic light scattering (DLS) and zeta potential (ELS), atomic absorption spectroscopy, photocatalysis, GC-MS and NMR analysis, computational studies, excited-state electron-transfer dynamics (PDF)
Crystal structure CoTTP (CIF)
