# Supplementary material for: Local Environmental Effects on Light-Driven CO2 Reduction in Liposomes
Source: ACS Catal. 2026 Feb 25;16(5):4362–72. doi: 10.1021/acscatal.5c03610 (PMC12973296; doi:10.1021/acscatal.5c03610)
Supplement: Supplementary file 1 [file cs5c03610_si_001.pdf]

Supporting Information

Local environment effects on light-driven CO_2 reduction in liposomes

Authors: Amir Abbas,[a,+] Richard Jacobi,[b,c,+] Ingrid Merker,[a] Riccarda Müller,[d] Nathaniel R. Ritz,[e] Nitish Kumar,[f] Hani M. Elbeheiry,[f] Dieter Sorsche,[a] Kerstin Leopold,[d] Leticia González,[b,g] Andrea Pannwitz[a,f,h,i]

[a] Institute of Inorganic Chemistry I, Ulm University, Albert-Einstein-Allee 11, 89081 Ulm, Germany

[b] Institute of Theoretical Chemistry, Faculty of Chemistry, University of Vienna, Währinger Straße 17, 1090 Vienna, Austria

[c] Doctoral School in Chemistry (DoSChem), University of Vienna, Währinger Straße 42, 1090 Vienna, Austria

[d] Institute of Analytical and Bioanalytical Chemistry (IABC), Ulm University, Albert-Einstein-Allee 11, 89081 Ulm, Germany

[e] Department of Chemistry, University of Michigan, Ann Arbor, MI 48109, USA

[f] Institute for Inorganic and Analytical Chemistry, Friedrich Schiller University Jena, Humboldtstraße 8, 07743 Jena, Germany

[g] Vienna Research Platform on Accelerating Photoreaction Discovery, University of Vienna, Währinger Straße 17, 1090 Vienna, Austria

[h] Center for Energy and Environmental Chemistry Jena (CEEC Jena), Friedrich Schiller University Jena, Philosophenweg 7a, 07743 Jena, Germany

[i] Helmholtz Institute for Polymers in Energy Applications Jena (HIPOLE Jena), Lessingstraße 12–14, 07743 Jena, Germany

+ These authors contributed equally.

Corresponding authors:

Leticia González leticia.gonzalez@univie.ac.at, Andrea Pannwitz andrea.pannwitz@uni-jena.de

Summary

1. Materials and Techniques 2

2. Synthesis and Characterization 3

2.1 Synthesis of (5,10,15,20-tetra(4-methylphenyl)porphinato)cobalt(II) (CoTTP) 3

2.2 Preparation of stock solutions 4

2.3 Vesicle preparation 5

2.4 Dynamic light scattering (DLS) and Zeta potential (ELS) 6

2.5 Atomic absorption spectroscopy 7

2.6 Spectroscopy 8

3. Photocatalysis 9

4. GC-MS and NMR analysis 11

5. Computational studies 19

6. Excited-state electron-transfer dynamics 23

References 29

1. Materials and Techniques

The synthesis of the photosensitizer, **RuC₉** and (5,10,15,20-tetra(4-methylphenyl)porphinato)cobalt(II) (**CoTTP**) were performed as described previously.[1–3]

The chemicals used for synthesis were commercially available from Merck KGaA. The porphyrins were purchased from PorphyChem. The chemicals were used without further purification. Matrix assisted laser desorption ionisation (MALDI) was performed on a Bruker solariX. Elemental analysis was performed by Mikroanalytisches Laboratorium Kolbe in Oberhausen, Germany. The elemental content of the molecules was reported as the elements' mass fraction percentage. ^{1}H-NMR spectra were recorded on a Bruker Ascend^{TM} 400 at a frequency of 400 MHz. Mestre Nova was used for the evaluation of the spectra. The chemical shift was given in ppm, the coupling constants J are given in Hertz (Hz). Multiplets were abbreviated as follows: s=singlet, d=doublet, t=triplet, q=quartet and m=multiplet. The residual peak of the respective deuterated solvent was used as internal reference. Sodium ascorbate 99%, sodium bicarbonate 98%, lithium carbonate 99% and cesium bicarbonate 99% were purchased from Sigma Aldrich, chloroform and dichloromethane with analytical grade of purity. Single-crystal X-ray diffraction studies were performed on a Bruker D8 Quest single-crystal diffractometer with a PHOTON II detector using a Mo-Kα radiation (wavelength λ = 0.71073 Å). CCDC 2394847 contains supplementary crystallographic data of **CoTTP**.

The lipids 1,2 dioleoyl-sn-glycero-3-phosphocholine (DOPC), 1,2-dimyristoyl-sn-glycero-3-phosphocholine (DMPC), 1,2-dipalmitoyl-sn-glycero-3-phosphocholine (DPPC), 1,2-dioleoyl-sn-glycero-3-phospho-(1'-rac-glycerol)(sodium salt) (DOPG), 1,2-dimyristoyl-sn-glycero-3-phospho-(1'rac-glycerol)(sodium salt) (DMPG), 1,2-dipalmitoyl-sn-glycero-3-phospho-(1'-rac-glycerol)(sodium salt) (DPPG) and 1,2-dimyristoyl-sn-glycero-3-phosphoethanolamine-N-[methoxy(polyethylene glycol)-2000] (14:0 PEG2000 PE), are depicted in the table below and were purchased from Avanti Polar.

Particle size measurements were performed with a Zetasizer Pro from Malvern Panalytical by Dynamic Light Scatttering (DLS)-Non Invasive Back Scatter (NIBS) using 900 µL of sample in a disposable polystyrene cell (DTS0012) with a measurement angle of 173°, 13° in a diameter range of 0.3 nm - 10 µm at 20 °C. Zeta potential measurements were performed with a Zetasizer Pro from Malvern Panalytical by Mixed-Mode Measurement phase analysis light scattering (M3-PALS) using a disposable folded capillary cell (DTS1070) in a diameter range of 3.8 nm – 100 µm.

High-resolution continuum source graphite furnace atomic absorption spectrometry (HR-CS-GFAAS) was performed using ContrAA 600 spectrometer (Analytik Jena GmbH) equipped with a graphite furnace atomization unit and MPE 60 auto sampler for liquid sampling (Analytik Jena GmbH). Argon with a purity of 99.996% (MTI, Neu-Ulm, Germany) was used as purge and protective gas.

Absorption spectroscopy was performed on a V-760 JASCO UV-Vis-NIR Spectrophotometer. [Gas-tight] Quartz glass cuvettes (d = 10.0 mm) were used for optical measurements. Typical experiments were performed at RT and aerobic conditions. All absorption spectra were recorded in bicarbonate solution with pH 8.19.

Photocatalysis was performed in a 3D printed photoreactor from project C6 in SFB project TRR234 "CataLight" network equipped with a LZ1-00DB00 High Efficacy 5W Dental Blue LED light-source (λ=460 nm, 800 to 1250 mW). CO and H_2 evolution were quantified using a Shimadzu GC-2030 with a barrier ionization discharge (BID-2030) detector (detection limit 0.1 ppm) and helium as carrier gas (column: Restek SH-Rt-Msieve 5A, ID: 0.32 mm; film thickness: 30 [micro]m, length: 30 m, oven temp. 80 °C by injecting manually 100 µL of the head space.

Emission intensity and lifetime measurements were performed, respectively, using an Horiba Jobin-Yvon FluoroMax Plus C automated benchtop spectrofluorometer equipped with a 150 W Xe arc excitation lamp (horizontal, continuous wave), a R13456 photon-counting PMT detector (190-930 nm) and a DeltaPro from Horiba Scientific using a 372 nm pulsed Laser source (Class 3B Laser Product, <0.5 W peak in pulsed and CW mode).

2. Synthesis and Characterization

2.1 Synthesis of (5,10,15,20-tetra(4-methylphenyl)porphinato)cobalt(II) (CoTTP)

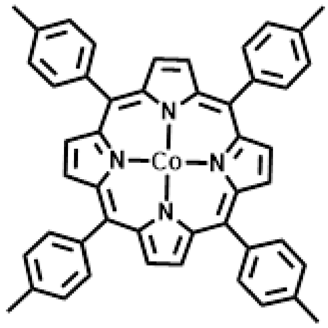

The metalation of 5,10,15,20-tetra(4-methylphenyl) porphyrin with cobalt acetate was performed according to the literature of Shen et al., 90 mL of DMF was purged with argon for 10 min. 53 mg (299 µmol, 1eq.) anhydrous cobalt acetate and 200 mg (298 µmol, 1 eq) 5,10,15,20-tetra(4-methylphenyl) porphyrin were added under argon reverse flow. The mixture was stirred for over 18 h under reflux and argon. DMF was removed under reduced pressure. The solid was dissolved in DCM and washed with water three times. The solid was purified by dry-loaded silica column with 4:1 n-hexane:DCM increasing the mixture to 1:1 n-hexane:DCM as eluent. **CoTTP** was obtained as brownish-red solid with a yield of 136.5 mg (187 µmol, 63%).

^{1}H-NMR (400 MHz, $CDCl_3$, NaAsc) δ=15.93 (s, 8H), 9.75 (s, 8H), 4.16 (s, 12H).

HRMS (MALDI-FT-ICR) m/z calculated for [$C_{48}H_{36}CoN_4$]: m/z=727.2272 found: m/z=727.2259.

Elemental Analysis: Calc: C 79.22%; H 4.99%; N 7.10%; Found: C 78.96%; H 5.01%; CN 7.60%.

Crystal structure: Asymmetric unit of the solid-state structure of **CoTTP**; the complex solvate CoTTP·Et2O crystallizes in the triclinic space group $P\overline{1}$ upon vapor diffusion of diethylether into a saturated acetonitrile solution of **CoTTP**.

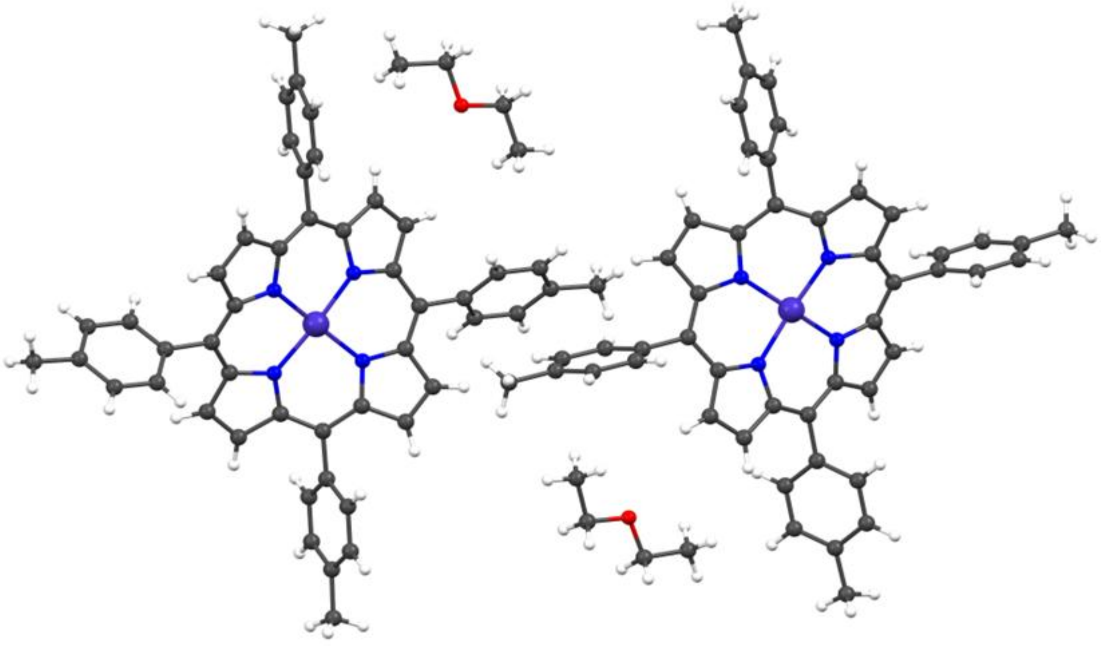

Table S1. Crystallographic parameters of **CoTTP**.

**Empirical formula**	$C_{48}H_{36}CoN_4$
**Formula weight**	727.23
**Temperature/K**	150
**Crystal system**	triclinic

**Space group**	P-1
**a/Å**	9.989(5)
**b/Å**	15.744(6)
**c/Å**	26.725(9)
**α/°**	79.573(15)
**β/°**	87.63(2)
**γ/°**	77.498(16)
**Volume/Å3**	4036(3)
**Z**	4
**ρ_{calc}g/cm^3**	1.32
**μ/mm^{-1}**	0.471
**F(000)**	1684
**Crystal size/mm^3**	0.323 × 0.222 × 0.134
**Radiation**	MoKα (λ = 0.71073)
**2Θ range for data collection/°**	6.84 to 52.744
**Index ranges**	-12 ≤ h ≤ 12, -19 ≤ k ≤ 19, -33 ≤ l ≤ 33
**Reflections collected**	64116
**Independent reflections**	16393 [Rint = 0.0730, Rsigma = 0.0649]
**Data/restraints/parameters**	16393/0/1057
**Goodness-of-fit on F^2**	1.022
**Final R indexes [I>=2σ (I)]**	R1 = 0.0466, wR2 = 0.1127
**Final R indexes [all data]**	R1 = 0.0676, wR2 = 0.1233
**Largest diff. peak/hole / e Å^{-3}**	0.90/-0.47

2.2 Preparation of stock solutions

For lipids solutions 99.8 mg of 18:1 (Δ9-Cis) PC (DOPC), 67.8 mg of 14:0 PC (DMPC), 73.4 mg of 16:0 PC (DPPC), 79.7 mg of 18:1 (Δ9-Cis) DOPG, 69.7 mg of 14:0 PG (DMPG), and 72.1 mg of 16:0 PG (DPPG) were respectively dissolved in 20 mL of chloroform, reaching a concentration of 5 mM. And 2.2 mg of 14:0 PEG2000 PE were dissolved in 20 mL of chloroform, reaching a concentration of 50 µM. Solutions were stored at 4 °C.

Sodium bicarbonate (8.4 g, 0.1 mol), was dissolved in 1 L of Milli-Q water, reaching a concentration of 0.1 M.

Potassium bicarbonate (10.0 g, 0.1 mol), was dissolved in 1 L of Milli-Q water, reaching a concentration of 0.1 M.

Lithium carbonate (7.4 g, 0.1 mol), was dissolved in 1 L of Milli-Q water, reaching a concentration of 0.1 M.

Cesium bicarbonate (19.4 g, 0.1 mol), was dissolved in 1 L of Milli-Q water, reaching a concentration of 0.1 M.

To prepare catalyst and photosensitizer solutions:

Catalyst **CoTTP** (1.59 mg, 2.19 nmol) was dissolved in 20 mL of chloroform, reaching a concentration of 110 µM.

RuC$_9$ (11.12 mg, 9.9 nmol) was dissolved in 20 mL of acetonitrile, reaching a concentration of 500 µM.

Electron donor solutions were prepared as follows:

Sodium ascorbate (396.2 mg, 2 mmol) was dissolved in 10 mL of sodium bicarbonate solution (0.1 M), reaching a concentration of 0.2 M.

Potassium ascorbate was prepared dissolving potassium bicarbonate (200.6 mg, 2 mmol) in 10 mL of ascorbic acid solution (0.2 M), reaching a concentration of 0.2 M.

Lithium ascorbate was prepared by dissolving lithium carbonate (74.6 mg, 1 mmol) in 10 mL of ascorbic acid solution (0.2 M), reaching a concentration of 0.2 M.

Cesium ascorbate was prepared by dissolving cesium bicarbonate (387.8 mg, 2 mmol) in 10 mL of ascorbic acid solution (0.2 M), reaching a concentration of 0.2 M.

Table S2*. Molecular structures, names, transition temperatures (T_m) and head group charge of investigated main phospholipids and the sterically stabilizing phospholipid 14:0 PEG2000 PE.*

	Phospholipid Structure	Name	T_m (°C)	Charge of the head group
1	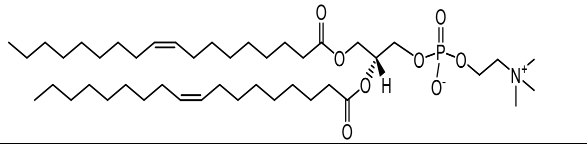	1,2-dioleoyl-sn-glycero-3-phosphocholine 18:1 (Δ9-Cis) PC (**DOPC**)	-17	Zwitterionic
2	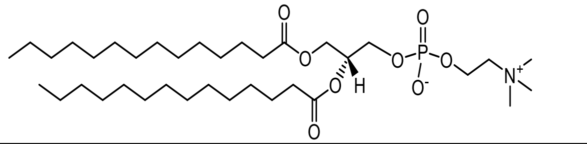	1,2-dimyristoyl-sn-glycero-3-phosphocholine 14:0 PC (**DMPC**)	24	Zwitterionic
3	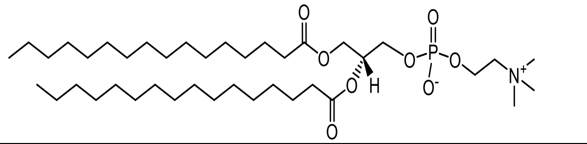	1,2-dipalmitoyl-sn-glycero-3-phosphocholine 16:0 PC (**DPPC**)	41	Zwitterionic
4	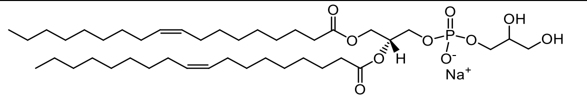	1,2-dioleoyl-sn-glycero-3-phospho-(1'-rac-glycerol)(sodium-salt) (**DOPG**)	-18	Negative
5	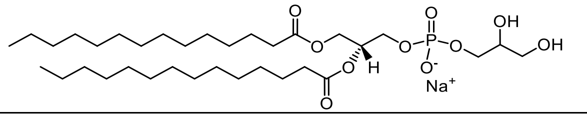	1,2-dimyristoyl-sn-glycero-3-phospho-(1'-rac-glycerol)(sodium salt)(**DMPG**)	23	Negative
6	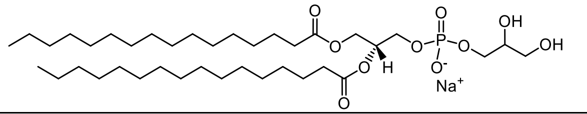	1,2-dipalmitoyl-sn-glycero-3-phospho-(1'-rac glycerol)(sodium salt) (**DPPG**)	41	Negative
7	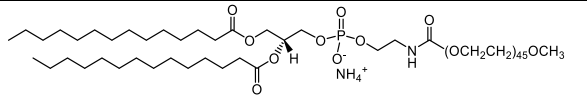	1,2-dimyristoyl-sn-glycero-3 phosphoethanolamine-N-[methoxy(polyethylene glycol)-2000] (**14:0 PEG2000 PE**)	n.a.	Negative

2.3 Vesicle preparation

In a typical procedure a mixture composed by 1 mL of 14:0 PEG2000 PE (50 µM), 1 mL of 18:1 (Δ9-Cis) PC (DOPC) (5 mM) and 200 µL of **RuC$_9$** (500 µM) and 90.8 µL of **CoTTP** (110 µM) was prepared. Solvents were removed by a rotovap and dried for 1 h at high vacuum using a Schlenk line. Then 1 mL of sodium bicarbonate solution (0.1 M) was added to the dried lipid film and followed by freeze-thaw cycles using liquid N_2 with a water bath at 10 ˚C above the transition temperature (T_m) of main lipid. This step was repeated 3-5 times until the solution was homogeneous. An extrusion was carried out (11 times) with a mini extruder and a heating block set from Avantes Polar Lipids using as filters Nucleopore Polycarbonate Track-Etch membrane with pore size of 200 nm and filter supports with pore size of 10 mm. A 0.4 mL sample of the liposome solution was purified by size exclusion chromatography (Sephadex^{TM} G25 superfine,

6 cm height, 2 cm diameter), 0.1 M sodium bicarbonate solution). Same procedure was applied for each lipid. After preparation every liposome sample was stored at 4 °C for a maximum of one week.

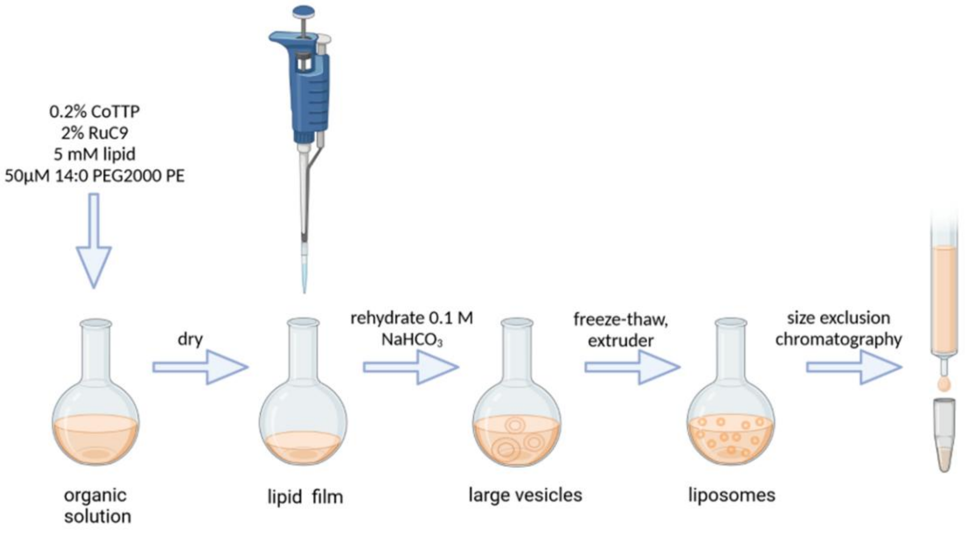

Figure S1. *A step-by-step view of liposomes preparation via thin film hydration procedure. Created with Biorender.com.*

2.4 Dynamic light scattering (DLS) and Zeta potential (ELS)

Particle size measurements were performed using 900 µL of sample in a disposable polystyrene cell (DTS0012) at 20 °C. Zeta potential measurements were performed using a disposable folded capillary cell (DTS1070). Every size measurement was performed in triplicate and the uncertainty was defined as one standard deviation. Size distribution was analyzed right after preparation of samples and after irradiation.

Table S3. *Overview on size distribution (Z-Average) in nm, polydispersity index (PDI) and zeta potential (ζ) of different liposomes samples before catalysis and after 24 h of irradiation.*

Host lipid	Z-Average (nm)		Polydispersity Index (PDI)		Zeta potential (mV)
	Before catalysis	After catalysis	Before catalysis	After catalysis	
**DOPC**	142.7±1.3	165.1±0.8	0.13±0.05	0.34±0.02	2.5±0.6
**DMPC**	146.4±0.4	136.6±1.5	0.08±0.01	0.14±0.01	-2.0±2.0
**DPPC**	145.4±1.8	160.1±2.0	0.05±0.03	0.10±0.03	6.8±0.9
**DOPG**	127.5±1.1	132.9±1.1	0.08±0.02	0.21±0.01	-27.5±0.3
**DMPG**	144.0±1.0	125.6±1.2	0.08±0.04	0.08±0.02	-40.0±1.0
**DPPG**	146.4±0.7	165.1±1.6	0.09±0.01	0.16±0.03	-61.4±0.9

Table S4. *Overview on size distribution (Z-Average) in nm and polydispersity index (PDI) of different liposomes samples before catalysis and after 24 h of irradiation.*

Host lipid	Cation	Z-Average (nm)		Polydispersity Index (PDI)	
		Before catalysis	After catalysis	Before catalysis	After catalysis
**DPPG**	**Na^+**	121.1±2.1	190.3±0.8	0.07±0.02	0.24±0.09
	**K^+**	129.4±1.3	167.1±2.2	0.11±0.07	0.36±0.05
	**Li^+**	139.3±0.9	517.4±1.1	0.14±0.03	0.80±0.11
	**Cs^+**	70.0±1.8	145.2±1.5	0.26±0.08	0.37±0.17
**DPPC**	**Na^+**	196.2±3.1	189.1±1.4	0.08±0.02	0.15±0.04
	**K^+**	186.4±1.5	257.3±1.1	0.13±0.05	0.51±0.02
	**Li^+**	177.1±5.4	500.2±4.3	0.16±0.03	0.56±0.05
	**Cs^+**	155.5±1.4	173.5±2.1	0.22±0.01	0.31±0.01

2.5 Atomic absorption spectroscopy

For HR-CS-GFAAS measurements the temperature programs as presented in **Table S5** and **Table S6** were applied. Three replicate measurements were performed by pipetting 20 µL of the respective solution into the graphite furnace tubes with dosing hole and without platform (Analytik Jena GmbH). The most sensitive lines of Ru (349.895 nm) and Co (240.752 nm) were selected, and Ru and Co mass concentrations were determined using external calibration ranging from 0.25 to 2.5 µg L^{-1} for Co and from 2 to 20 µg L^{-1} for Ru. For this purpose, Co and Ru stock standard solution of 5 µg L^{-1} and 20 µg L^{-1}, respectively, were prepared by dilution of 1000 mg L^{-1} standard solutions (VWR International GmbH, Darmstadt, Germany).

For quantification of Co and Ru in liposomes the samples were prepared as follows. 1000 µL of liposome solution was acidified by adding 100 µL precleaned concentrated HNO_3 (AnalaR NORMAPUR®, VWR International GmbH). For HR-CS-GFAAS analysis, the samples are diluted by adding an appropriate volume of 10 % HNO_3.

Table S5. *Used temperature program for the Co determination by HR-CS-GFAAS.*

**Step**	**Name**	**Temperature / °C**	**Ramp / °C s^{-1}**	**Hold / s**	**Time / s**	**Gas int.**
**1**	Drying	80	6	20	44.2	Max
**2**	Drying	90	3	20	50.0	Max
**3**	Drying	110	5	10	60.0	Max
**4**	Pyrolysis	350	50	20	44.3	Max
**5**	Pyrolysis	700	100	20	16.5	Max
**6**	Pyrolysis	1200	300	20		Max
**7**	Gas adjustment	1200	0	5	5.0	Stopp
**8**	Atomization	2100	1500	4.6	7.1	Stopp
**9**	Clean out	2450	500	4.7	4.1	Max

Table S6. *Used temperature program for the Ru determination by HR-CS-GFAAS.*

**Step**	**Name**	**Temperature / °C**	**Ramp / °C s^{-1}**	**Hold / s**	**Time / s**	**Gas int.**
**1**	Drying	85	6	35	44.2	Max
**2**	Drying	95	1	40	50.0	Max
**3**	Drying	110	1	45	60.0	Max
**4**	Pyrolysis	550	15	15	44.3	Max
**5**	Pyrolysis	1000	300	15	16.5	Max
**6**	Gas adjustment	1000	0	5	5.0	Stopp
**7**	Atomization	2700	1500	6	7.1	Stopp
**8**	Clean out	2750	500	4	4.1	Max

2.6 Spectroscopy

Table S7. *Overview of the influence of different lipid environments on absorption maxima observed under air and inert conditions. Experimental conditions: 50 µM **CoTTP** in 5 mM neutral liposomes (DOPC, DMPC, DPPC) and negatively charged liposomes (DOPG, DMPG, DPPG).*

	**Soret band**		**Q-band**	
**Matrix**	**λ_{Abs} (nm)**	**$\lambda_{Abs,\ inert\ conditions}$ (nm)**	**λ_{Abs} (nm)**	**$\lambda_{Abs,\ inert\ conditions}$ (nm)**
**DOPC (T_m < rt)**	412	412	535	529
**DMPC (T_m = rt)**	426	412	-	-
**DPPC (T_m > rt)**	425	412	-	528
**DOPG (T_m < rt)**	412	413	546	534
**DMPG (T_m = rt)**	408	413	545	-
**DPPG (T_m > rt)**	413	411	-	537
**Organic solvent (DCM)**	412	-	529	-

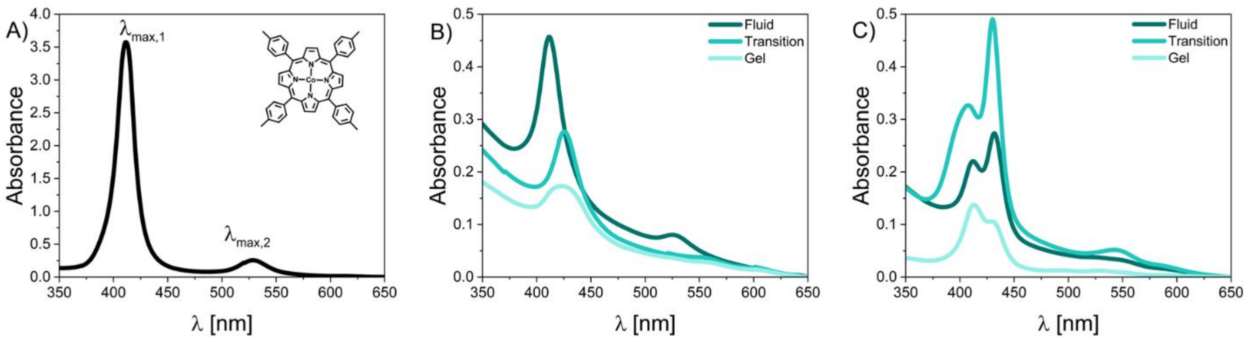

Figure S2. *Absorption spectra of A) 50 µM **CoTTP** in dichloromethane. B) 50 µM **CoTTP** in 5 mM neutral liposomes (DOPC, DMPC, DPPC). C) 50 µM **CoTTP** in 5 mM negative liposomes (DOPG, DMPG, DPPG).*

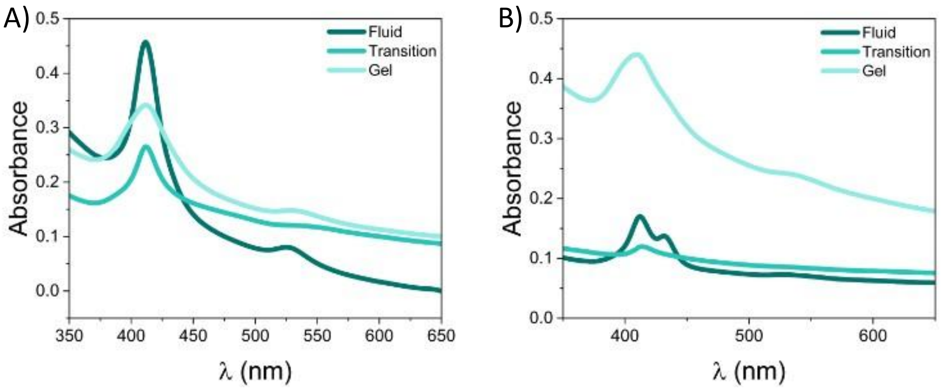

Figure S3. *Absorption spectra under inter conditions of A) 50 µM **CoTTP** in 5 mM neutral liposomes (DOPC, DMPC, DPPC). B) 50 µM **CoTTP** in 5 mM negative liposomes (DOPG, DMPG, DPPG).*

3. Photocatalysis

In a typical preparation 120 µL of liposomes solution at 5 mM main lipid concentration or molecular components at the corresponding concentrations, 1 mL of sodium ascorbate solution (0.2 M) and 880 µL of bicarbonate solution (0.1 M), were combined in an 8 mL WICOM WIC 41600/333 clear glass screw neck GC vial (ND13) at final concentration of 0.3 mM of the main lipid. The vial was then sealed, degassed with CO_2 (30 min, average final pH 6.9±0.1) via careful bubbling with a needle and placed into a 3D printed photoreactor (current 0.7 A and voltage of 4 V), and irradiated by LZ1-00DB00 High Efficacy 5W Dental Blue LED light-source (λ=460 nm, 800 to 1250 mW). The experiment was performed at room temperature using a ventilation system. Over 24 h, the evolved CO and H_2 were detected *via* head-space gas chromatography. Turnover number data were calculated from the ratio between moles of CO and H_2 produced and moles of photosensitizer or catalyst.

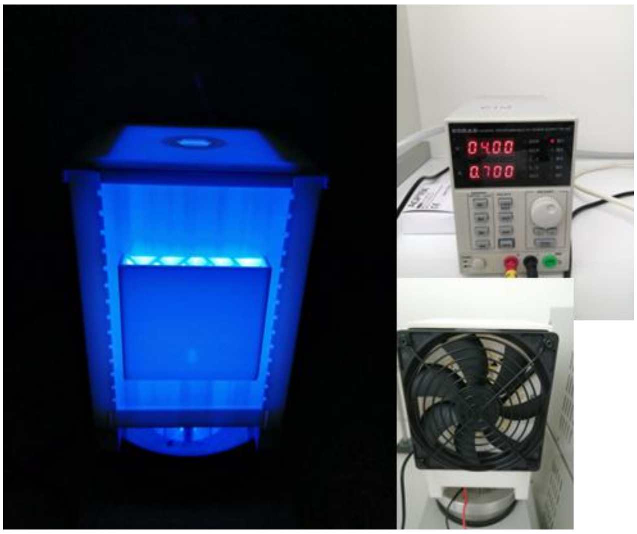

Figure S4. *3D printed photoreactor setup for the light-driven CO_2 reduction, equipped with an LED emitter at λ = 460 nm. The irradiation module was placed beneath the sample holder and the ventilation system was placed in the back of the photoreactor. The sample holder was located at the same distance from the LED emitter in every experiment.*

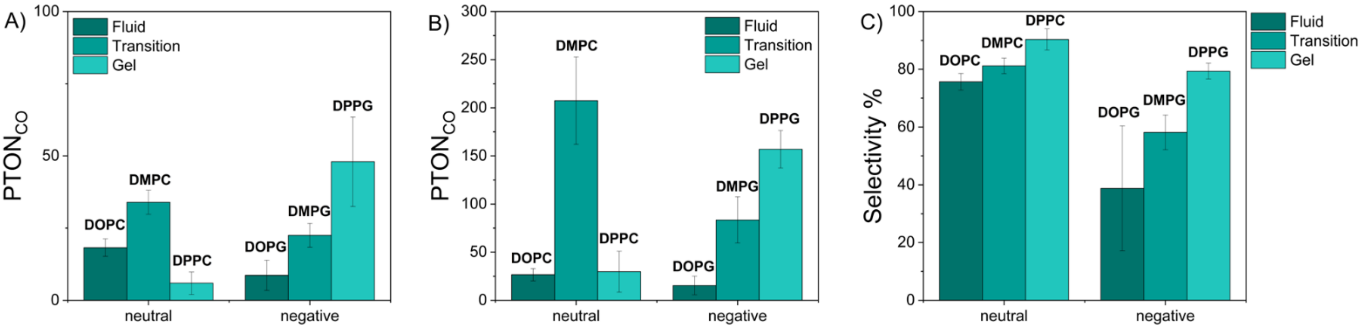

Fig. S5. *A). PTON$_{CO}$ results, after 24 h, of the light-active liposomes (neutral: DOPC, DMPC, DPPC; negative: DOPG, DMPG, DPPG), under irradiation of 460 nm LED and in presence of sodium ascorbate M as sacrificial electron donor. B) PTON$_{CO}$ calculated with the actual uptake of* ***RuC$_9$*** *determined by atomic absorption spectrometry (HR-CS-GFAAS). C) Corresponding selectivity (%) of the photocatalytic systems. Experimental conditions: liposomes sample in CO_2 atmosphere with a composition of 100:1:2:0.2 of main lipid:(14:0 PEG2000 PE):****RuC$_9$:CoTTP****, with c(main lipid) = 0.3 mM and c(sodium ascorbate) = 0.1M. All values were acquired in triplicate with error bars presenting the standard deviation.*

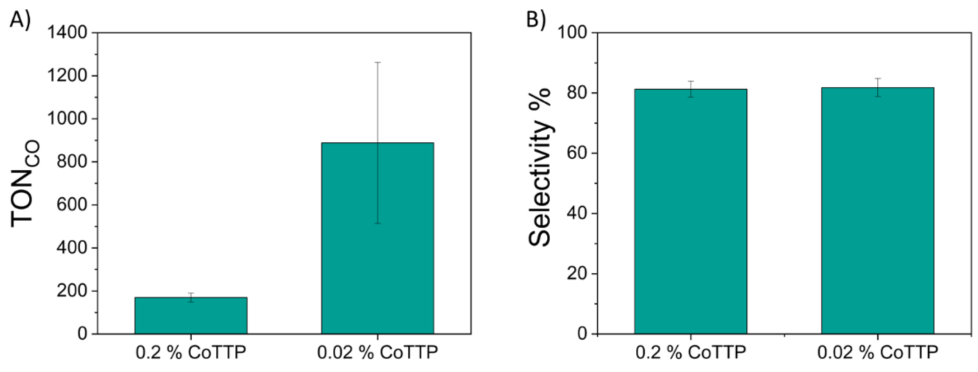

Figure S6. *A) TON$_{CO}$ results and B) selectivity, after 24 h, of the light-active liposomes (neutral: DMPC), under irradiation of 460 nm LED and in presence of sodium ascorbate M as sacrificial electron donor. Experimental conditions: liposomes sample in CO_2 atmosphere with a composition of 100:1:2:0.2 or 100:1:2:0.02 of main lipid:(14:0 PEG2000 PE):**RuC$_9$:CoTTP**, with c(main lipid) = 0.3 mM and c(sodium ascorbate) = 0.1M. All values were acquired in triplicate with error bars presenting the standard deviation.*

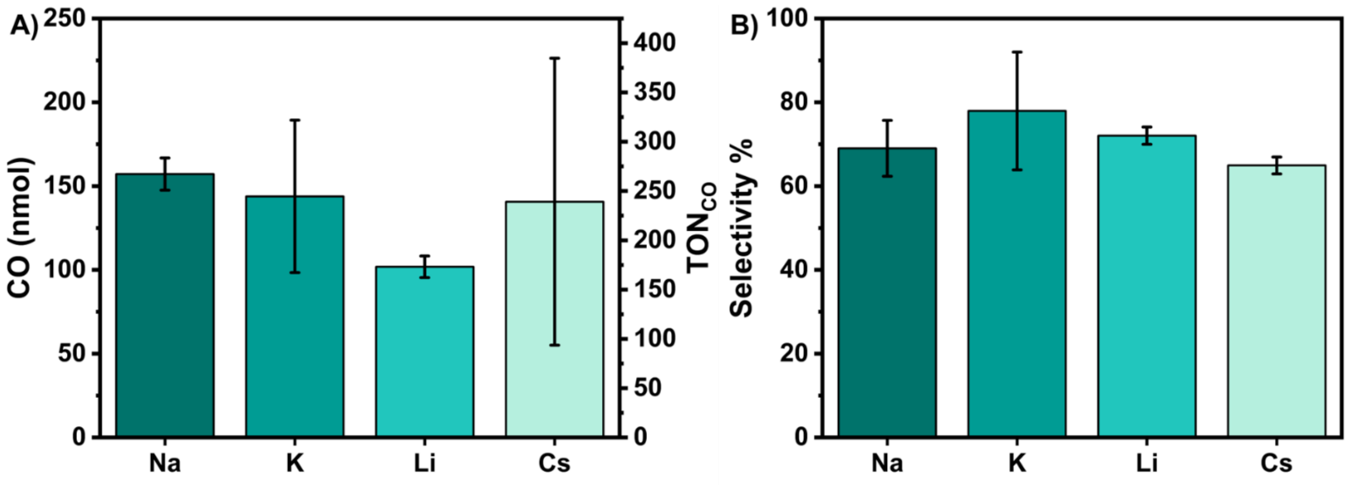

Figure S7. *A) TON$_{CO}$ results, after 24 h, of the light-active liposomes (negative: DPPG), under irradiation of 460 nm LED and in presence of ascorbate M as sacrificial electron donor (Na^+ replaced with K^+, Li^+ or Cs^+). B) Corresponding selectivity (%) of the photocatalytic systems. Experimental conditions: liposomes sample in CO_2 atmosphere with a composition of 100:1:2:0.2 of main lipid:(14:0 PEG2000 PE):**RuC$_9$:CoTTP**, with c(main lipid) = 0.3 mM and c(ascorbate) = 0.1M. All values were acquired in triplicate with error bars presenting the standard deviation.*

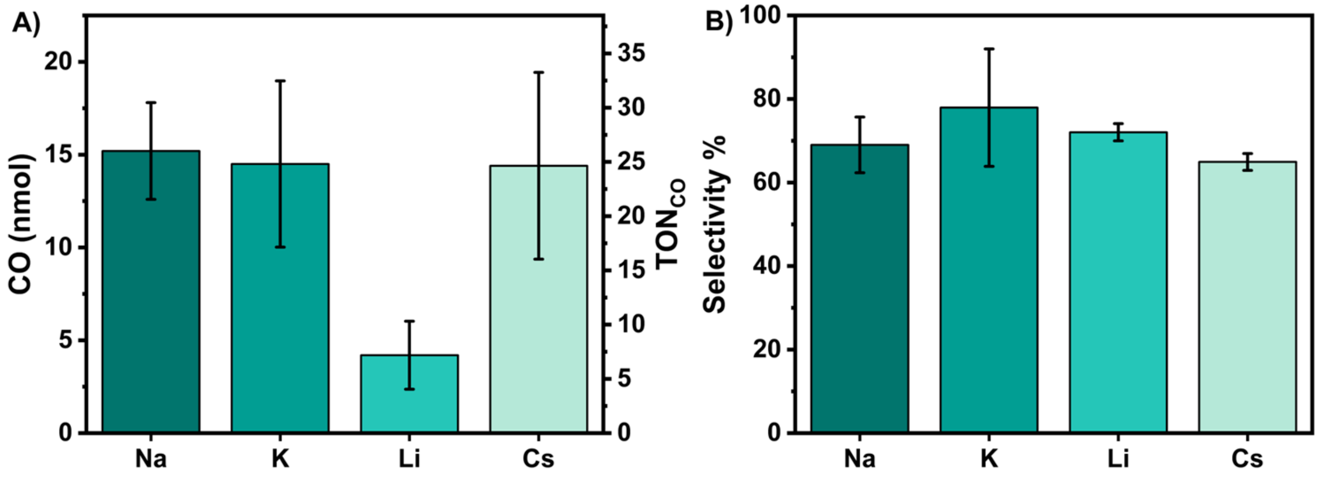

Figure S8. *A) TON$_{CO}$ results, after 24 h, of the light-active liposomes (neutral: DPPC), under irradiation of 460 nm LED and in presence of ascorbate M as sacrificial electron donor (Na^+ replaced with K^+, Li^+ or Cs^+). B) Corresponding selectivity (%) of the photocatalytic systems. Experimental conditions: liposomes sample in CO_2 atmosphere with a composition of 100:1:2:0.2 of main lipid:(14:0 PEG2000 PE):**RuC$_9$:CoTTP**, with c(main lipid) = 0.3 mM and c(ascorbate) = 0.1 M. All values were acquired in triplicate with error bars presenting the standard deviation.*

4. GC-MS and NMR analysis

GC-MS analysis

To actually confirmed that the carbon monoxide produced in the photocatalytic reaction originates from external bubbled CO_2 not from decomposition of ascorbate or other components, we conducted a series of controlled experiments using ^{13}C isotope labeling reagents.

GC-MS set up: The headspace gas was analyzed using a Shimadzu GC-MS QP 2010 Plus system. Prior to injection, the gas samples were pretreated with a HS-20 headspace sampler, operating at an oven temperature of 60 °C and a sample transfer line temperature of 150 °C. The injected headspace gas was separated using RT-Qbond column (30 m length, 0.32 mm diameter, 10 µm film thickness) and detected by electron ionization mass spectrometry (EI-MS).

Sample preparation: In this study, we used two types of bicarbonate buffer solutions. One containing ^{13}C-labeled sodium bicarbonate ($NaH^{13}CO_3$, 0.1 M) and the other is a non-labeled buffer ($NaH^{12}CO_3$, 0.1 M). Both buffers were treated with a sodium hydroxide concentration of 0.04 M, reaching the pH of 6.9. For a typical sample preparation a 300 µL of liposomes with the main lipid at 5 mM, 2200 µL of the respective bicarbonate buffer (labeled or non-labeled) and 2500 µL of sodium ascorbate solution (0.2 M, dissolved in same buffer) were mixed directly into the 20 ml of screw neck GC-MS vial (vial size: 75.5 x 22.5mm). The vial was sealed, degassed with Ar for 20 minutes, and purged with labeled or non-labeled CO_2 gas for 10 minutes. The vial was then placed into a 3D printed photoreactor and irradiated by a blue LED light-source (λ = 460 nm, 800 to 1250 mW). The experiment was performed at room temperature using a ventilation system. After 24 hrs of irradiation the headspace gases were analyzed using the direct headspeace injection in the GC-MS.

In this experiment, we used the best performing liposomes based on DPPG and DMPC lipids. For each lipid, four different controlled samples were prepared as follows

Sample 1. Purged with $^{13}CO_2$ gas and ^{13}C labeled sodium bicarbonate buffer.

Sample 2. Purged with $^{13}CO_2$ gas and non-labeled ^{12}C sodium bicarbonate buffer.

Sample 3. Purged with $^{12}CO_2$ and ^{13}C labeled sodium bicarbonate buffer.

Sample 4. Purged with $^{12}CO_2$ and non-labeled ^{12}C sodium bicarbonate buffer.

Sample 5. Standard air injection as an instrumental background noise.

The headspace GC-MS analysis detected CO, N_2, Ar, and O_2 appearing as the first peak at the retention time of 2.1 minutes. A second peak was observed at the retention time of 2.8 minutes that is assigned to $^{13}CO_2$/ $^{12}CO_2$.

First conclusion from the results of this experiment is the exclusion of methane, ethane, formaldehyde, formic acid, and other products with higher number of carbons. The following chromatograms show only the presence of ^{13}CO as a product of the photocatalytic reaction.

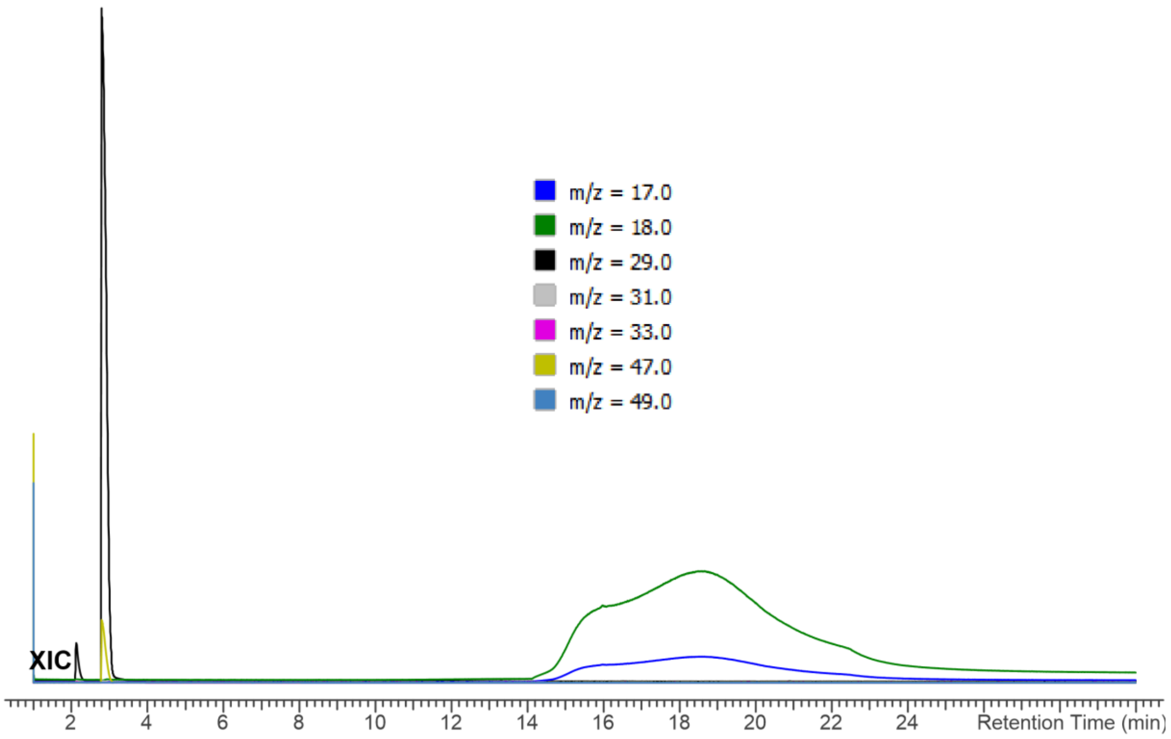

Figure S9. *Extracted ion chromatograms from GC-MS total ion chromatogram obtained from headspace injection after photocatalytic* $^{13}CO_2$ *reduction in a DMPC-based liposomes in a* ^{13}C *labelled buffer. The sample composition is 100:1:2:0.2 of main lipid:(14:0 PEG2000 PE):**RuC9:CoTTP**, with c(main lipid) = 0.3 mM and c(ascorbate) = 0.1 M.*

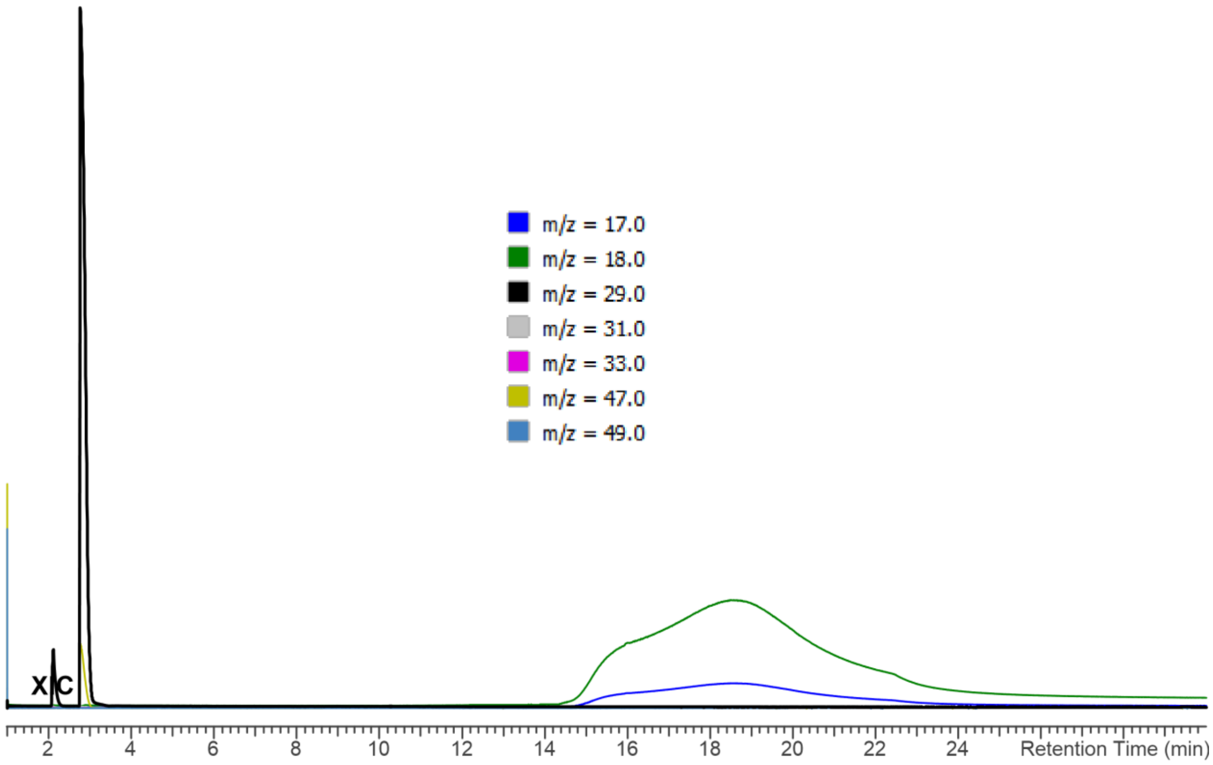

Figure S10. *Extracted ion chromatograms from GC-MS total ion chromatogram obtained from headspace injection after photocatalytic* $^{13}CO_2$ *reduction in a DPPG-based liposomes in a* ^{13}C *labelled buffer. The sample composition is 100:1:2:0.2 of main lipid:(14:0 PEG2000 PE):**RuC$_9$:CoTTP**, with c(main lipid) = 0.3 mM and c(ascorbate) = 0.1 M.*

Second conclusion is that these results confirm the source of CO. For each sample, the extracted ion chromatogram for the ions m/z = 28 and m/z = 29 (^{13}CO) were obtained. The area under the peak for each ion and the area ratio of the 29-ion to the 28-ion were calculated (sample chromatograms are presented in Figures S11-14). The results are summarized in Table S8. The data show an enhancement in the ^{13}CO signal only when $^{13}CO_2$ was introduced to samples. This confirms that the source of CO is CO_2 and not any other source in the reaction mixture.

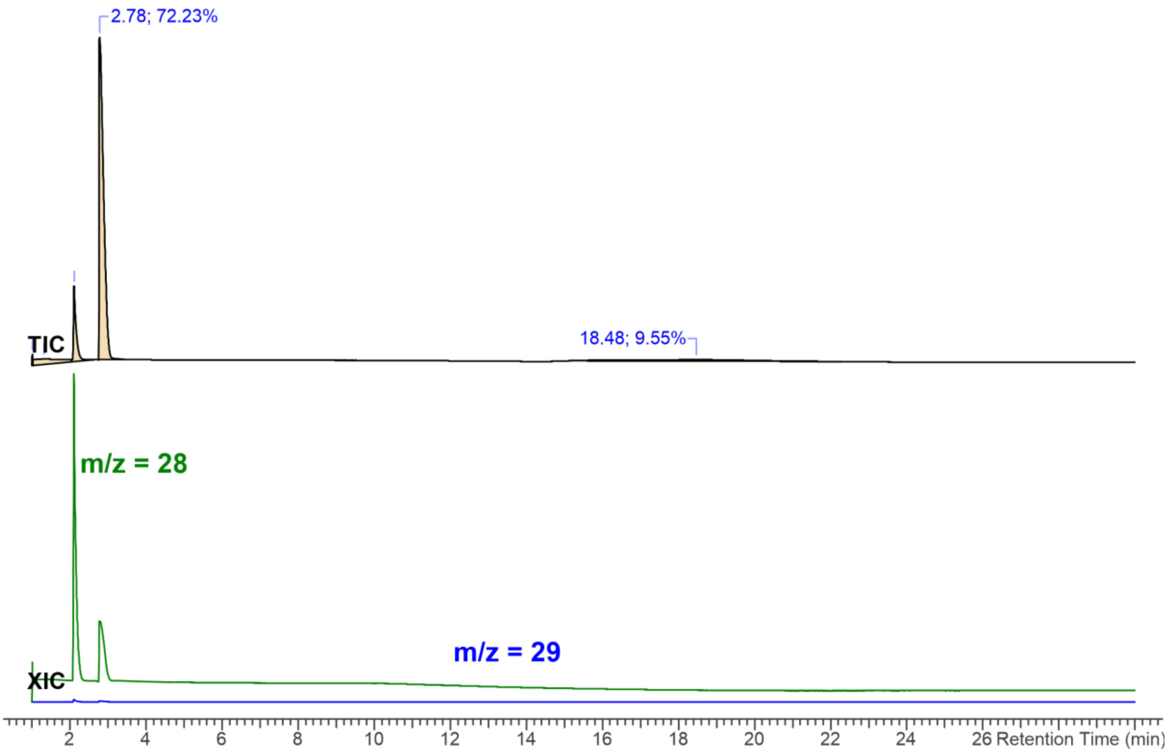

Figure S11. *Extracted ion chromatograms of ions m/z = 28 and m/z = 29 from GC-MS total ion chromatogram obtained from headspace injection after photocatalytic* $^{12}CO_2$ *reduction in a* ***DMPC****-based liposomes in a non-labelled buffer. The sample composition is 100:1:2:0.2 of main lipid:(14:0 PEG2000 PE):****RuC9:CoTTP****, with c(main lipid) = 0.3 mM and c(ascorbate) = 0.1 M.*

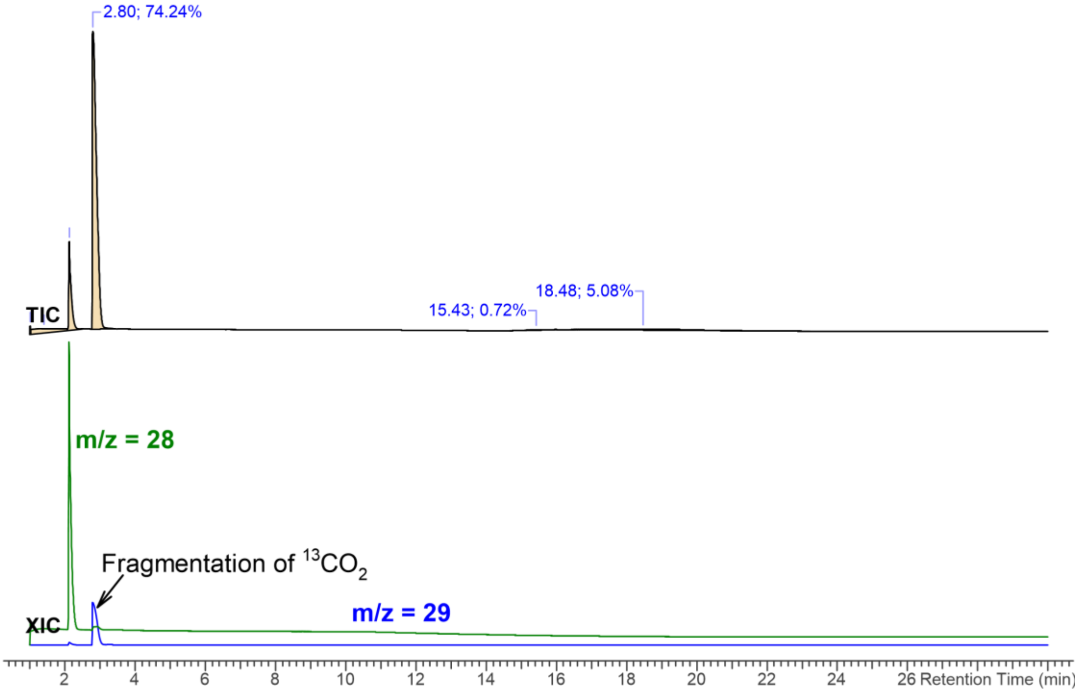

Figure S12. *Extracted ion chromatograms of ions m/z = 28 and m/z = 29 from GC-MS total ion chromatogram obtained from headspace injection after photocatalytic* $^{13}CO_2$ *reduction in a* ***DMPC****-based liposomes in a* ^{13}C *labelled buffer. The sample composition is 100:1:2:0.2 of main lipid:(14:0 PEG2000 PE):****RuC9:CoTTP****, with c(main lipid) = 0.3 mM and c(ascorbate) = 0.1 M.*

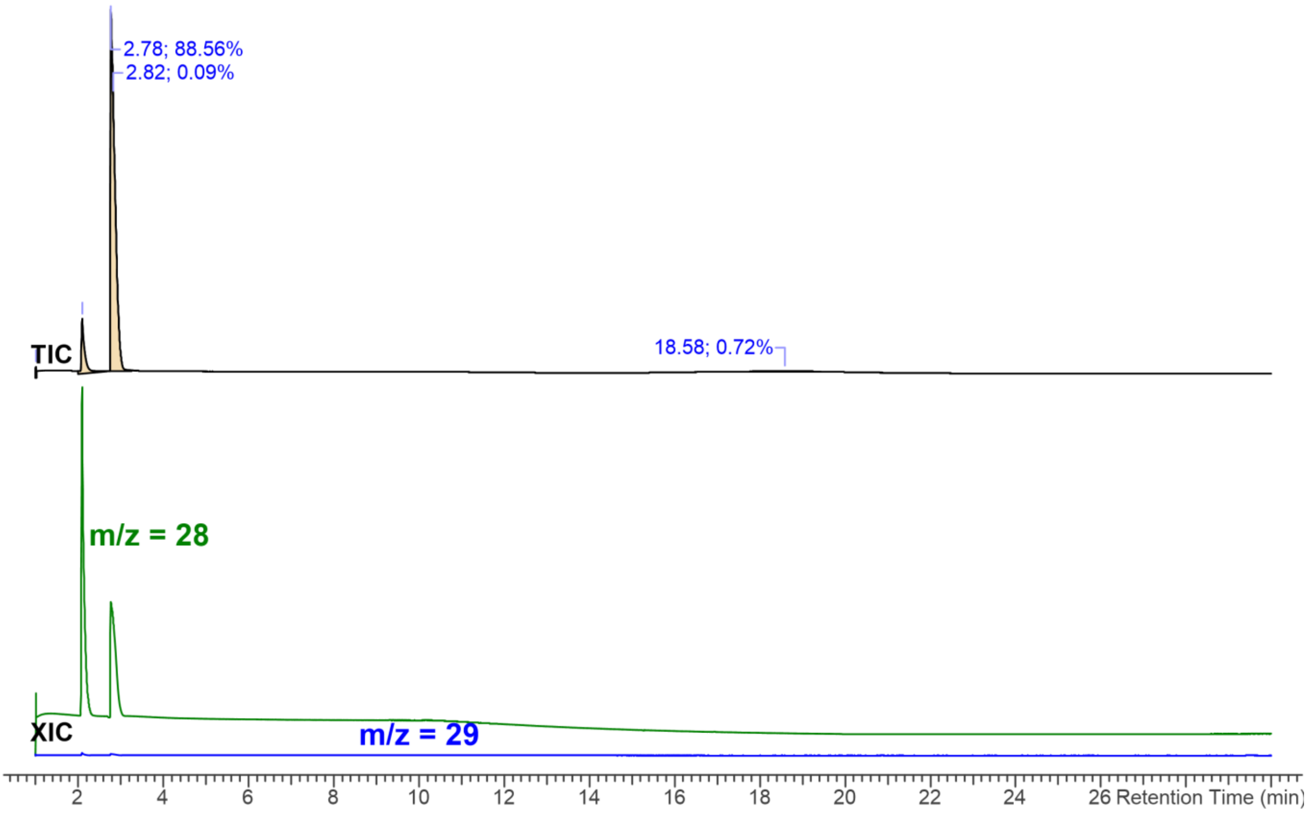

Figure S13. *Extracted ion chromatograms of ions m/z = 28 and m/z = 29 from GC-MS total ion chromatogram obtained from headspace injection after photocatalytic $^{12}CO_2$ reduction in a **DPPG**-based liposomes in a non-labelled buffer. The sample composition is 100:1:2:0.2 of main lipid:(14:0 PEG2000 PE):**RuC9:CoTTP**, with c(main lipid) = 0.3 mM and c(ascorbate) = 0.1 M.*

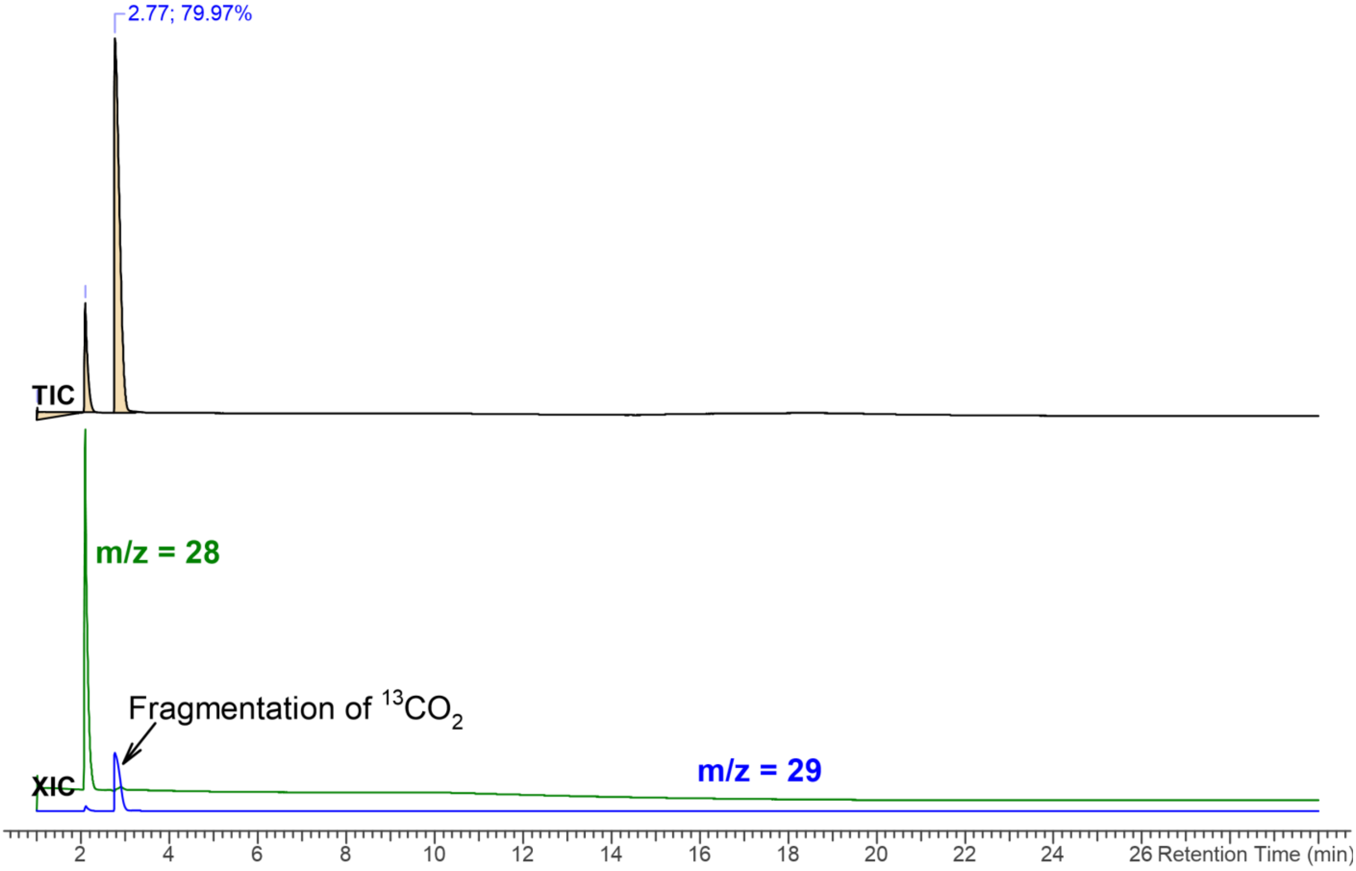

Figure S14. *Extracted ion chromatograms of ions m/z = 28 and m/z = 29 from GC-MS total ion chromatogram obtained from headspace injection after photocatalytic $^{13}CO_2$ reduction in a **DPPG**-based liposomes in a ^{13}C labelled buffer. The sample composition is 100:1:2:0.2 of main lipid:(14:0 PEG2000 PE):**RuC9:CoTTP**, with c(main lipid) = 0.3 mM and c(ascorbate) = 0.1 M.*

Table S8. *Summary of the areas calculated from the extracted ion chromatograms of the ions m/z = 28 and m/z = 29 (^{13}CO) together with the ratio between the area of the 29-ion to the 28-ion. Experimental conditions: sample composition is 100:1:2:0.2 of main lipid:(14:0 PEG2000*

*PE):**RuC9:CoTTP**, with c(main lipid) = 0.3 mM and c(ascorbate) = 0.1 M, where the main lipid is DMPC or DPPG. Samples were prepared in 13C labelled buffer or non-labelled buffer with $^{13}CO_2$ or $^{12}CO_2$ bubbling.*

Lipid	Entry	Area under m/z = 28	Area under m/z = 29	$(Area_{29}/Area_{28}) \cdot 100$
**DMPC**	$^{12}CO_2$, non-labelled buffer	1667461632	12407999	0.744
	$^{12}CO_2$, labelled buffer	1628266752	11632801	0.714
	$^{13}CO_2$, non-labelled buffer	1241323904	13852204	1.116
	$^{13}CO_2$, labelled buffer	2148104885	23195560	1.080
**DPPG**	$^{12}CO_2$, non-labelled buffer	907541376	6582293	0.725
	$^{12}CO_2$, labelled buffer	2327163392	16556083	0.711
	$^{13}CO_2$, non-labelled buffer	1151056128	22722435	1.974
	$^{13}CO_2$, labelled buffer	2262711552	35098792	1.551
**Air**	Background of the instrument	847396416	6214189	0.733

NMR analysis

After 24 hours of photocatalytic reaction, the aqueous phase was analyzed by NMR spectroscopy to check if any other CO_2 reduction products are formed other then CO. To easily detect the presence of these products using $^{13}C\{^1H\}$ NMR, the experiment was performed with ^{13}C labeled bicarbonate buffer and $^{13}CO_2$ gas.

To establish a clear baseline, we first recorded reference ^1H NMR spectra of the blank reaction mixture using excitation sculpting with gradients to suppress the water signal. These reference measurements allowed us to identify if there is any trace CO_2 reduction products already present in the system prior to photocatalysis. The reference ^1H NMR spectra were submitted for (0.1 M $NaH^{13}CO_3$ with 0.04 M NaOH, Pink Figure S15), DMPC:(14:0 PEG2000 PE) = 100:1 (DMPC 300 µM in buffer, Black Figure S15), DPPG:(14:0 PEG2000 PE) = 100:1 (DPPG 300 µM in buffer, Red Figure S15), sodium ascorbate (0.2 M in buffer, Blue Figure S15). It is important to note that the spectra shown in Figure S15 have been scaled relative to the suppressed water signal peak. Therefore, the signal intensities do not directly reflect actual concentrations. We detected a weak formate signal peak at 8.36 ppm (Figure S15), which can be attributed to small amounts of formate already present in the sodium ascorbate of the labelled buffer.

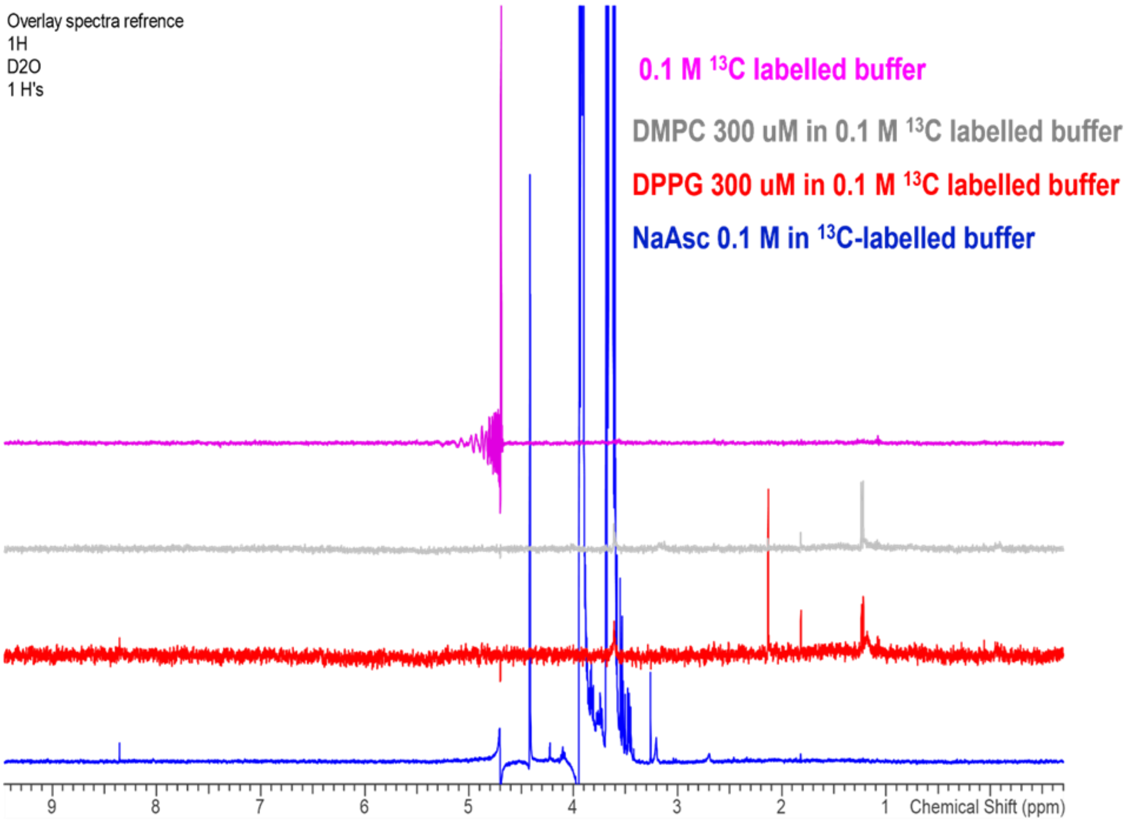

Figure S15. *^{1}H NMR spectra of sodium ascorbate (0.2 M in ^{13}C labelled buffer, Blue), DMPC (300 µM in ^{13}C-labelled buffer, Black), DPPG (300 µM in ^{13}C-labelled buffer, Red), 0.1 M ^{13}C-labelled buffer Pink.*

^{1}H NMR spectra were recorded for the photocatalysis samples both with and without irradiation. The samples for NMR spectroscopy were prepared by following the procedure. First, 2 mL of DPPG$_{\mathbf{PS\text{-}CAT}}$ liposomes were prepared in sodium ascorbate (0.2 M) ^{13}C labelled buffer using a molar ratio of 100:1:2:0.2 for DPPG, (14:0 PEG2000 PE), PS, and CAT, with DPPG at 300 µM concentration. After the liposome formation in a 5-mL vial the sample was bubbled with $^{13}CO_2$ for 7 min. For quantitative analysis, 400 µL of the liposomal solution was taken and mixed with 100 µL of a maleic acid-D_2O solution (11.4 mM) in an NMR tube, where maleic acid served as the internal standard (IS). This sample was submitted for measuring ^{1}H NMR and ^{13}C NMR before irradiation (Figure S16, red). The rest of the liposomal sample was sealed in the vial and was irradiated for 24 hours. Then again 400 µL of the irradiated sample were mixed with 100 µL of maleic acid-D_2O solution (11.4 mM) in an NMR tube. The ^{1}H NMR and ^{13}C NMR spectra were collected from this tube as the NMR spectrum after 24 hr of irradiation (Figure S16, blue). Chemical shifts and peak integrals in figure S16 are calibrated against maleic acid ($\delta_^{1}H$ = 5.90 ppm, 2H) as an internal reference. The formate peak ($\delta \approx 8.36$ ppm) showed an integral increase from 0.0259 H (before irradiation) to 0.0303 H (after irradiation), corresponding to $\Delta I = 0.0044$ H. Using the maleic acid-D_2O internal standard calibration, this change in integral translates to an estimated formate concentration of around 12.5 µM. Based on literature a minimum concentration of 150 µM of formate is required for precise quantification using ^{1}H NMR spectroscopy.[4] As the detected amount of formate in NMR tube was far below this amount, the formate concentration cannot be determined by ^{1}H NMR spectroscopy. The ^{13}C NMR spectrum showed the characteristic resonance signals of sodium ascorbate at δ_13C = 62.64, 69.60, 78.35, 113.85, 175.56, and 177.51 ppm,[5] confirming the presence of ascorbate in the sample. Additionally, the peaks for ^{13}C-labeled HCO_3^- and dissolved $^{13}CO_2$ were observed at 160.28 and 24.63 ppm, respectively.[6] The maleic acid shows two signal peaks at 130.52 ppm and 173.30 ppm, assigned to its olefinic and carboxyl carbon respectively.[7–9] Since, we also do not observe any formate peak at 171.1 ppm[10] in the ^{13}C NMR spectrum (Figure S17), the formate detected in the ^{1}H NMR measurements is unlikely to arise from direct $^{13}CO_2$ reduction. Instead, it likely appears from background formate originates from the minor decomposition of ascorbate. This observation is in line with the headspace GC-MS that excludes the formation of formic acid.

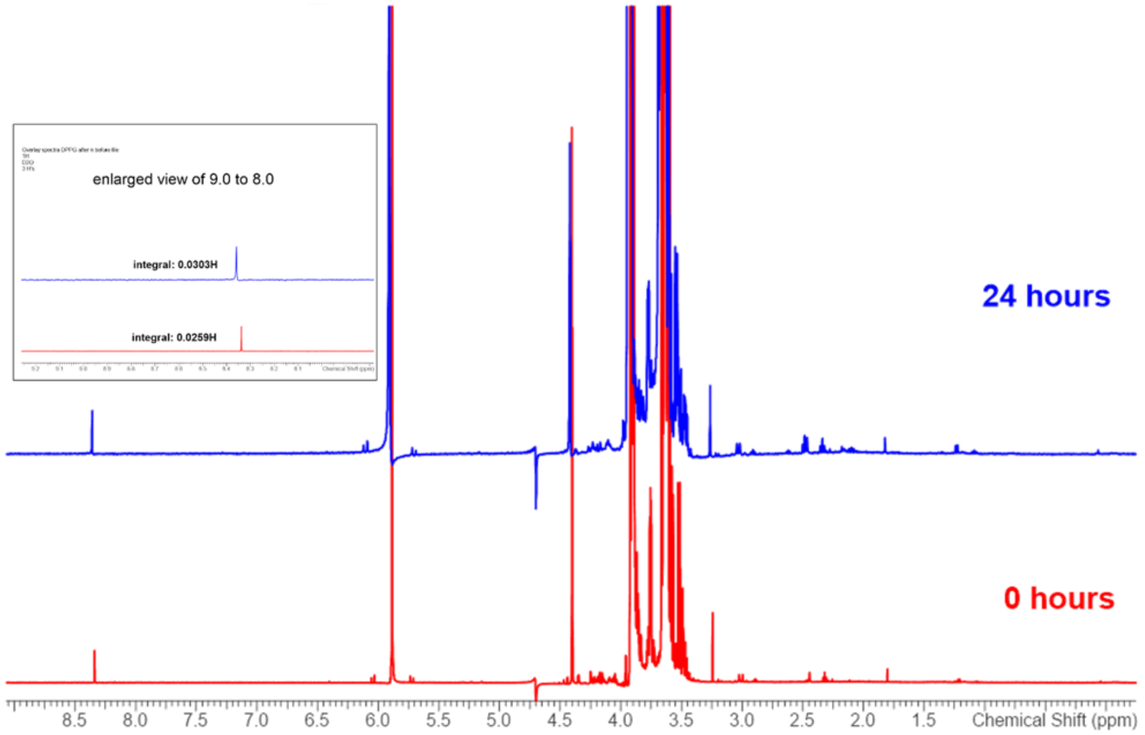

Figure S16. *^{1}H NMR spectra of DPPG photocatalytic samples without light irradiation (red spectrum) and with 24 hours of irradiation (blue spectrum).*

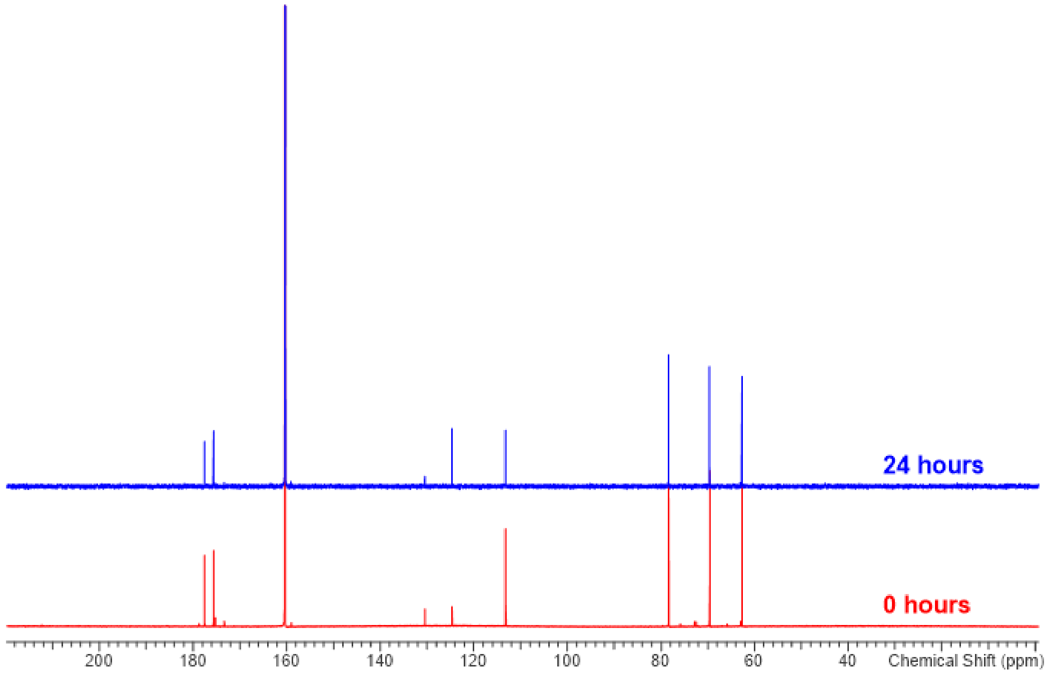

Figure S17. *^{13}C NMR spectra of DPPG photocatalytic samples without light irradiation (red spectrum) and after 24 hours of irradiation (blue spectrum).*

The same experimental procedure was performed in the case of DMPC lipid and similar results were observed (Figure S18). The formate peak (δ ≈ 8.36 ppm) in ^{1}H NMR spectra showed an integral increase from 0.0242H to 0.0309 H (after irradiation), corresponds to ΔI = 0.0067 H. Using the calibrated maleic acid-D_2O internal standard, this change in integral translates to an estimated formate concentration of approximately 19 µM which was again too low to accurately quantitfy the amount of formate. No other higher hydrocarbon products were found in aqeous solution as is seen in the ^{13}C NMR as well.

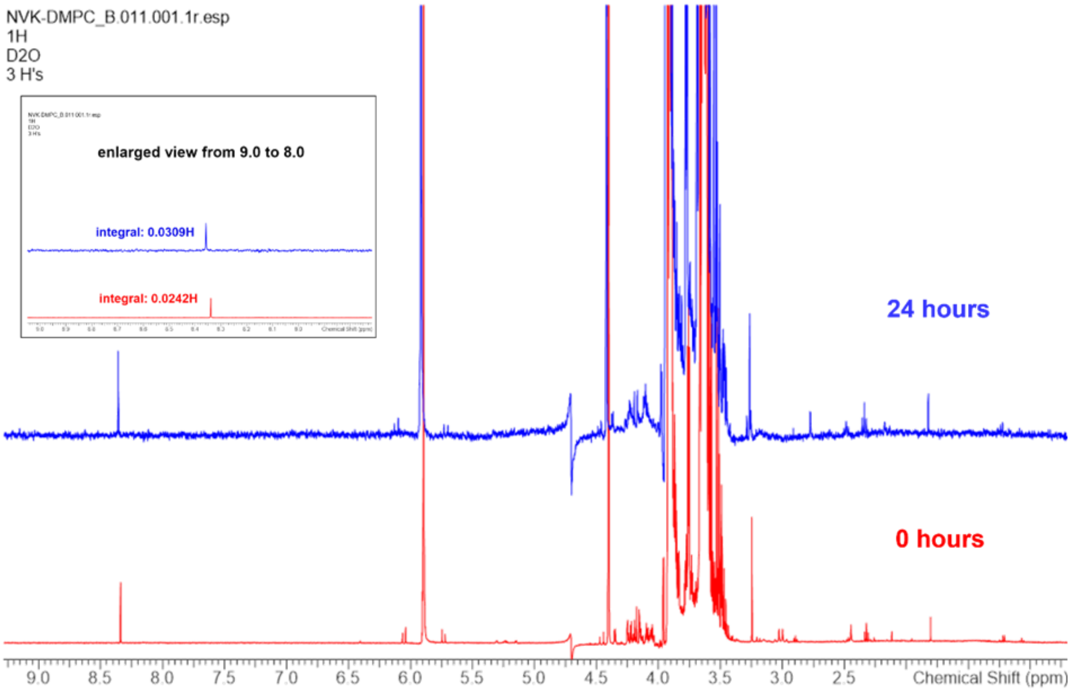

Figure S18. *¹H NMR spectra of DMPC photocatalytic samples without light irradiation (red spectrum) and after 24 hours of irradiation (blue spectrum).*

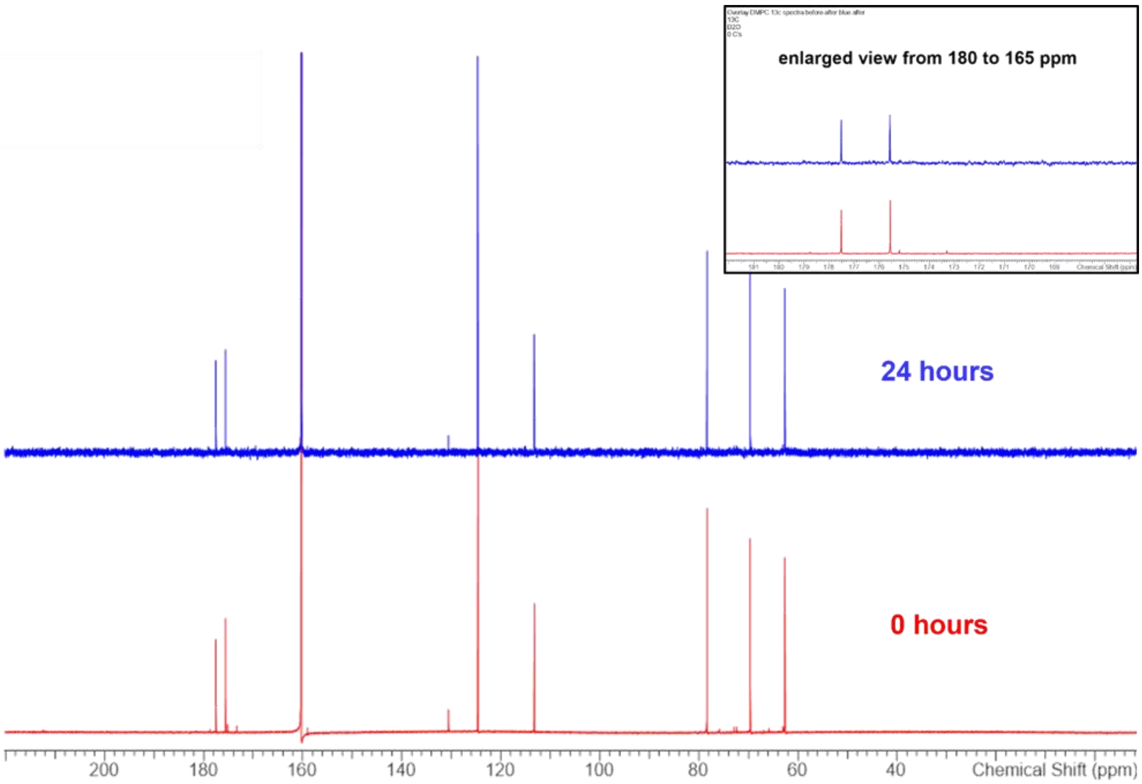

Figure S19. *¹³C NMR spectra of DMPC photocatalytic samples without light irradiation (red spectrum) and with 24 hours of irradiation (blue spectrum).*

5. Computational studies

The molecular dynamics simulations are performed according to a simulation protocol that has been used by some of us before.[2] The initial systems are generated using the membrane builder of the web-based input generator CHARMM-GUI.[11,12] Each lipid bilayer is constructed in the xy-plane of an orthorhombic simulation box with side lengths in x- and y-directions set to 80 Å, resulting in, depending on the lipid type, around 100 lipid molecules in each leaflet. The membrane is solvated in water to attain a hydration number of 50, which is well in excess of experimental hydration numbers (eg. 32.8 for DOPC or 26.6 for DMPC),[13] resulting in a box height of roughly 90 Å. **CoTTP** is placed upright into one of the leaflets, such that two phenyl groups touch the head groups and the other two are located directly at the membrane center. The negatively charged lipids are neutralized by adding potassium ions, and in all systems KCl ion pairs are added to reach a salt concentration of 0.15 M.

All simulations are performed using the program package Amber22 and AmberTools22[14] using the GPU (CUDA) version of pmemd.[15–17] **CoTTP** is described by the Generalized Amber Force Field[18] with additional parameters for Cobalt adapted from an iron-based cytochrome,[19] as has been previously done in literature.[20] Atomic charges for **CoTTP** are included as computed using B3LYP[21,22]/def2-SVP[23,24]-D3BJ[25] with the CHelpG charge scheme[26] in implicit water modeled with the conductor-like polarizable continuum model,[27,28] in Gaussian 16, Revision C.01.[29] The phospholipids are described using the LIPID21[30] force field, while water and ions are described using the optimal point charge (OPC)[31,32] force field as implemented in AmberTools21. The cutoff for non-bonded interactions is set to 10 Å.

Throughout, periodic boundary conditions at constant pressure are enforced using anisotropic pressure scaling. Prior to simulation, the energy of each system is minimized in 5000 steps employing a steepest descent algorithm, and additional 5000 steps using a conjugate gradient algorithm. For all simulations, the time step is set to 2 fs. To achieve this large time step, the SHAKE algorithm[33,34] is used to freeze the bond lengths of bonds containing hydrogen at a relative tolerance of 1e-7.

At first, the systems are heated from 0 to 100 K in 5 ps (2,500 steps) and from 100 to 300 K in 100 ps (50,000 steps) using the Langevin thermostat at a collision frequency of 1 ps^{-1}. The pressure relaxation time is increased to 2 ps for the second heating step only and set to 1 ps in all other simulation steps. Prior to trajectory production, each system is equilibrated for 10 ns (5,000,000 steps). Subsequently, the systems are simulated for 1.4 µs (700,000,000 steps) each, and simulation frames every 0.1 ns are saved for the analysis (14,000 frames per trajectory). The analysis is performed using the CPPTRAJ program[35] included in AmberTools22.

Vertical reduction energies are computed using a quantum mechanics/molecular mechanics hybrid approach with electrostatic embedding. The Amber22 interface to Gaussian 16 is employed to compute the electronic energy of **CoTTP** with B3LYP/def2-SVP. The effect of the environment on **CoTTP** is represented as point charges. In two separate computations on the same geometry, **CoTTP** is once computed in its neutral doublet state, and once in its reduced, singly negatively charged singlet state. The vertical reduction energies are obtained as the difference between the final self-consisting field energies. This computation is performed on 100 structures (one snapshot every 14 ns) for each system.

The density profiles for Co in the neutral and negative lipid bilayer membranes are shown in Figure S20. In the top row, the real-space density profiles as they result from the molecular dynamics simulations are shown, with the center of each membrane, ie. half the distance between the head group density peaks, set to zero. For this, the head group densities of each system are fitted with two gaussian functions, one for the upper and one for the lower leaflet, and the center of this gaussian is used as the head group density peak. As can be seen in Figure S20, **CoTTP** does not remain in one of the leaflets, but rather crosses between the two. This crossing is more or less pronounced in the different lipids, which makes it harder to compare the densities. To alleviate this discrepancy, positive and negative values at the same distance to the membrane center are added and displayed as their absolute values for all density profiles in the middle row of Figure S20, making use of the symmetry of a phospholipid bilayer membrane. In this way, the Co density in either lipid can be compared. This representation allows to analyze the different distances to the membrane center, but not to the interface between the membrane surface and the aqueous bulk solution, as the membranes are of different widths. The distance to this interface is displayed in the bottom row of Figure S20, where the peaks of the head group densities are set to position zero. Now, the Co density profiles extend up to different distances, in turn displaying the different widths of the membranes. In Figure 4 of the main manuscript, these different membrane widths are accounted for by scaling (dividing) all distances of the symmetrized density profiles (middle row of Figure

S20) by half the respective membrane width, such that the membrane center remains at 0, and the head group density peaks are at a relative distance of the membrane center of 1. The corresponding widths are displayed in Figure S21. All density profiles of Co both in the main manuscript as well as here in the Supporting Information are smoothened using a spline function, as they originally are quite noisy due to the low Co concentration, while the computation of the average insertion depth is performed on the original data.

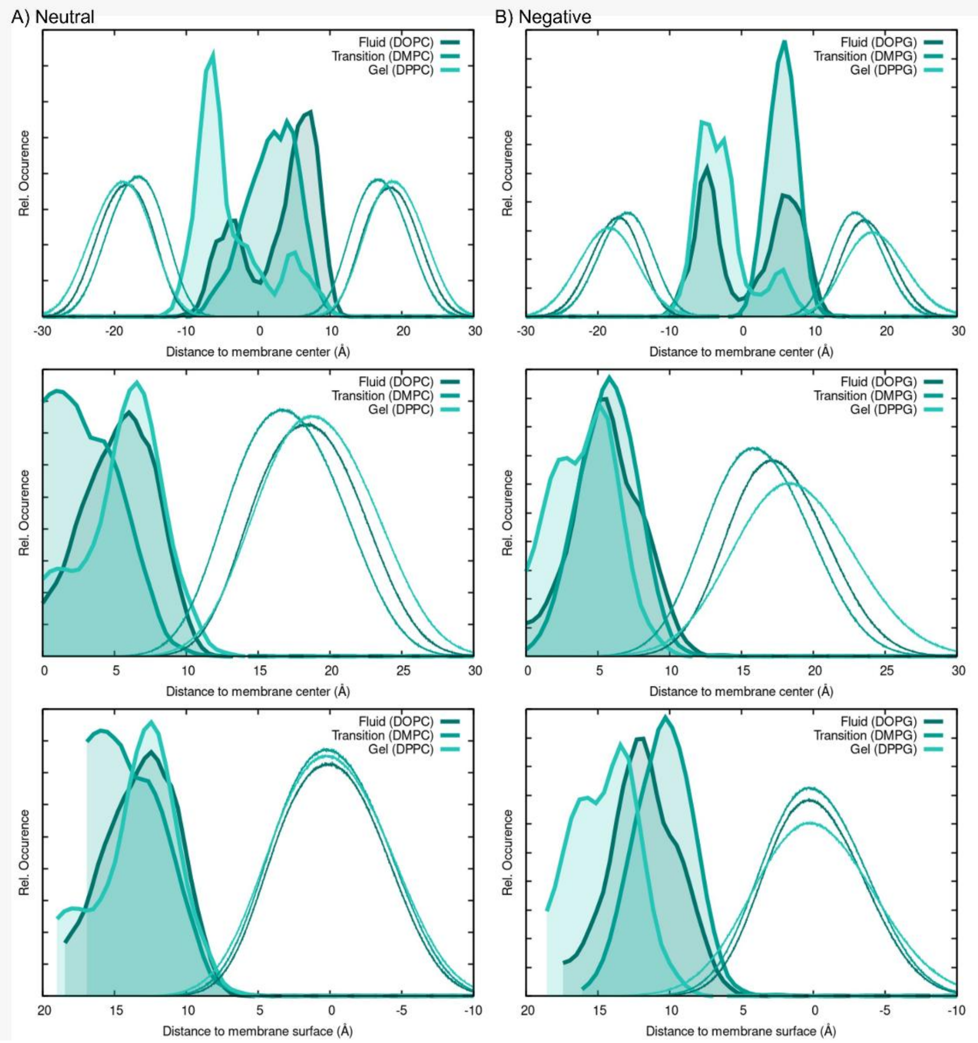

Figure S20. *Computed density profiles for A) the neutral and B) the negatively charged liposomes. Top row: Raw density profiles. Middle row: Symmetrized density profiles. Bottom row: Density profiles with respect to the aqueous interface.*

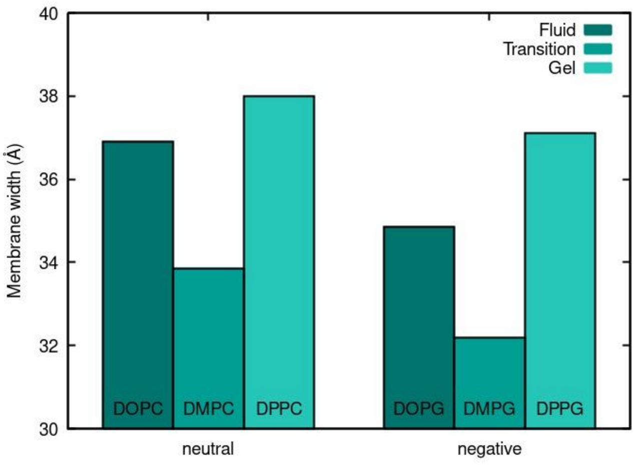

Figure S21. *Computed width of the phospholipid bilayers.*

Figure S22 shows the histograms of the angle between the normal vector of the membrane and the normal vector of the **CoTTP** molecular plane. For this representation, the normal vector to the membrane surface is represented by the z-axis, as the lipid bilayer membranes by design of the simulation box are assembled in the xy-plane and remain there

due to the periodicity of the simulation box. The normal to the molecular plane is computed as the dot product between the two vectors connecting each of the two opposing Nitrogen atoms around the metal center. In this way, the angle between these two normal vectors shows how upright **CoTTP** is within the membrane. Angles close to 90° signify that **CoTTP** is upright in the membrane, while angles close to 0° correspond the molecular plane of **CoTTP** being parallel to the surface of the membrane. Overall, there are only little differences between the liposomes. In the negative liposomes, lower angles are slightly more common than in the neutral liposomes, and the DO-liposomes seem to enforce lower angles more than the DM or DP variants do.

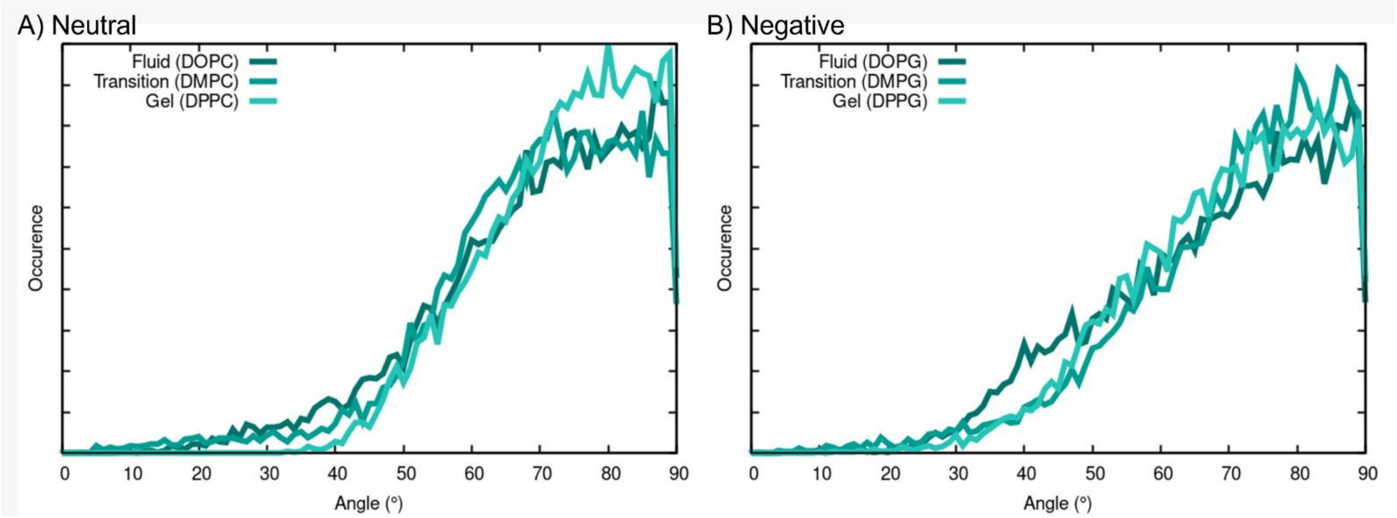

Figure S22. *Computed angle between the normal vector to the membrane surface and the normal vector to the **CoTTP** plane.*

Figure S23 shows angles and dihedrals that describe the distortion away from planarity of **CoTTP** in the different lipids. The top row shows the histograms of the N-Co-N angle as indicated in the pictogram (and its symmetry equivalent, both added to the same histogram). For all lipids, the angles are very close to 180°, which indicates that these five atoms are mostly in the same plane. The middle row shows the C-Co-C angle as indicated in the histogram (and its symmetry equivalent both added to the same histogram). The range of angles here is significantly larger, which is caused by out-of-plane distortions of **CoTTP** along these axes. However, this distortion is not in the same direction for both the symmetry-equivalent angles, but in opposite directions, such that the shape of the molecular plane can be compared to a saddle (x^2-y^2) with the saddle point located in the Cobalt center. Again, this distortion of **CoTTP** occurs in all liposomes with no measurable differences. Displayed in the bottom row is the dihedral between two N-N connecting vectors as indicated in the pictogram. This dihedral accounts for twists of the molecular plane which are not accounted for by either of the preceding angles. However, this twist is not present in **CoTTP** as evidenced by the histograms close to 180°. In general, it can be concluded that the geometry of **CoTTP** is not influenced to a measurable degree by the liposome environment.

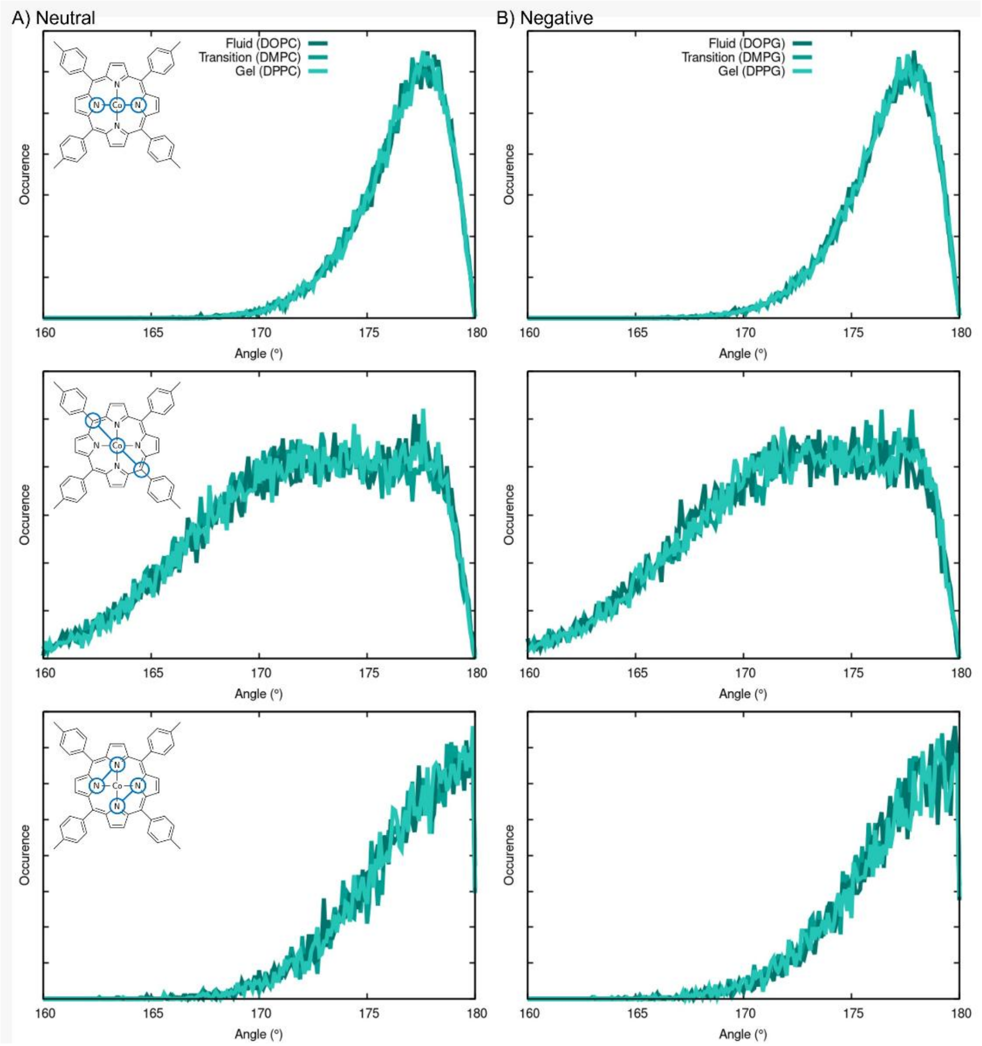

Figure S23. *Computed measures of geometric distortions of* ***CoTTP*** *in A) the neutral and B) the negatively charged liposomes. Top row: N-Co-N angle. Middle row: C-Co-C angle. Bottom row: Dihedral of the four nitrogen atoms.*

Figure S24 shows radial distribution functions (RDFs) of all non-**CoTTP** atom types around the cobalt center in the different lipids. The carbon RDFs exhibit a periodic signature, which most probably stems from the individual lipid tails. The relative order of phosphorus RDFs of the different liposomes is governed by the insertion depth (compare Figure S20). **CoTTP** in DMPC and DPPG exhibit the largest distance to the surface of the membrane and thus have the lowest values for the phosphorous RDFs, as phosphorous is part of the lipid head groups. Similar trends can be observed for the other lipids and the remaining RDFs, though the ordering is not always directly correlated with the insertion depths. In general, aside from the carbon RDFs, all RDFs start to increase only at comparably high distances, confirming that the electrostatic environment of the Cobalt atom is dominated by the hydrophobic aliphatic tails.

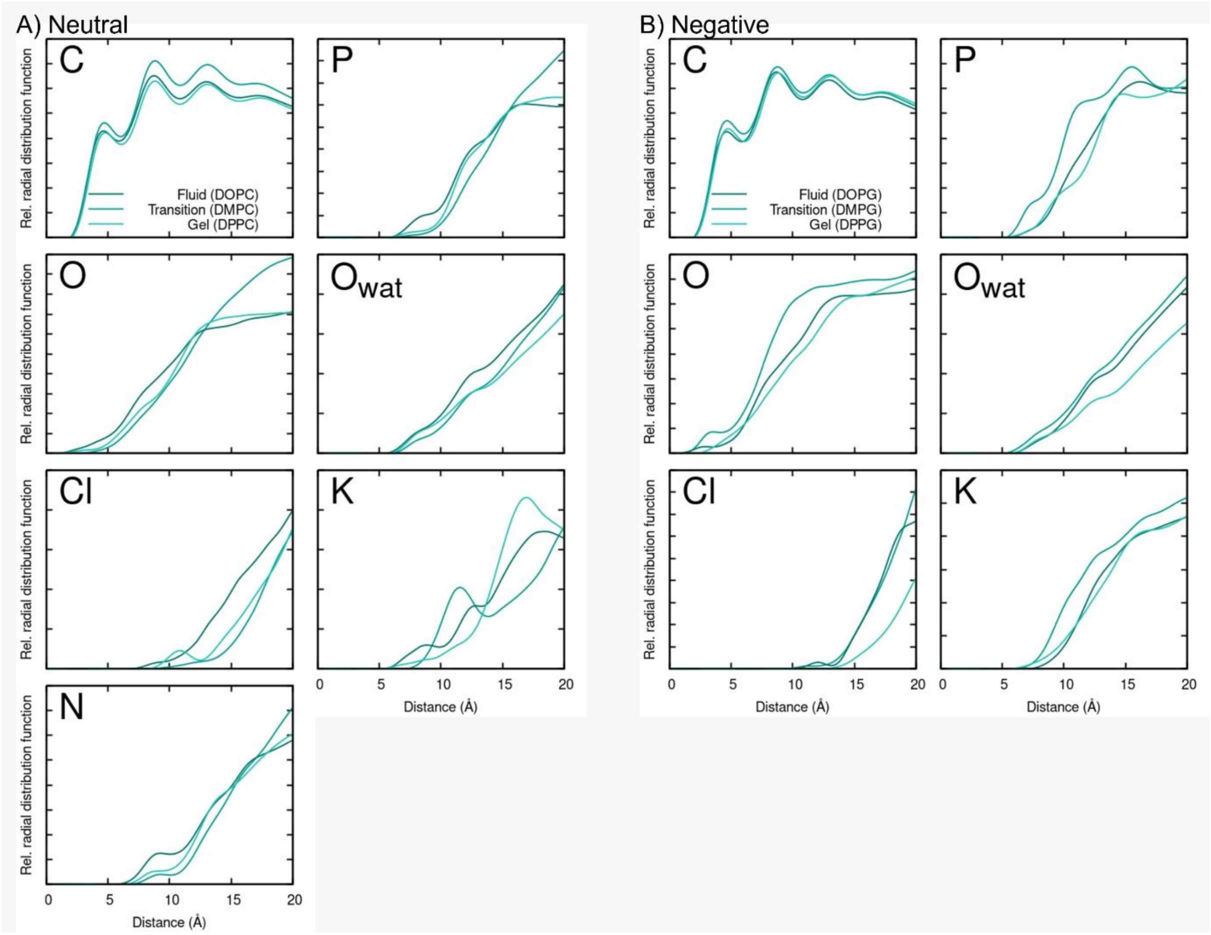

Figure S24. Computed radial distribution functions around the Co center in A) the neutral and B) the negatively charged liposomes for carbon, phosphorus, oxygen, oxygen (water only), chlorine, potassium and nitrogen (only neutral head groups). Atoms of **CoTTP** are not included in the analysis.

6. Excited-state electron-transfer dynamics

Samples were prepared using quartz glass cuvettes (d = 10.0 mm). Typically, 200 µL of liposomes solution at 5 mM of main lipid, with only **RuC₉** presence, and 2.8 mL of CO_2 saturated sodium bicarbonate solution were combined. Increasing aliquots of sodium ascorbate 0.8 M were added right before the emission intensity and lifetime measurements. Similarly, 200 µL of liposomes solution at 5 mM of main lipid, with different catalyst loadings, and 2.8 mL of CO_2 saturated sodium bicarbonate solution were combined to prepare the samples for each measurement. 0.5 mL of ascorbic acid 0.6 M were added to each sample right before the emission intensity and lifetime measurements. The Stern-Volmer equation was applied (see eq. 1), to obtain the K_{sv} and k_q values. Based on literature and previous experimental works on similar devices, the excited state lifetime error was estimated to be at 10 %. Standard deviation of K_{sv} was calculated, a general formula of error propagation theory was applied to calculate k_q standard deviation (eq. S1):

$$\frac{\Delta c}{c} = \frac{\Delta a}{a} + \frac{\Delta b}{b} \quad (S1)$$

where $\frac{\Delta c}{c}$ is the relative error and Δc the standard deviation of the quotient.

Table S9. *Summary of Stern-Volmer constants, quenching rate constants and lifetime from mechanistic studies. Experimental conditions: neutral (DOPC, DMPC and DPPC) and negative (DOPG, DMPG and DPPG) liposome samples with: i) a composition of 100:1:2 of main lipid:(14:0 PEG2000 PE):**RuC_9**, with c(main lipid) = 0.3 mM, containing sodium ascorbate as sacrificial electron donor. ii) and iii) a composition of 100:1:2:X of main lipid:(14:0 PEG2000 PE):**RuC_9**:**CoTTP** with c(main lipid) = 0.3 mM, at various loadings of **CoTTP**, in absence and presence of sodium ascorbate (0.1 M) as sacrificial electron donor, respectively. Samples were prepared in CO_2-saturated 0.1 M bicarbonate solution. Values are reported as mean values +/- standard deviation.*

**Sample**	**Host lipid**	**K_{sv} (L mol^{-1})**	**k_q (L mol^{-1} s^{-1})**	**τ_0 (ns)**	**Quenching mechanism**
**i)**	**DOPC**	3.0±0.9	$(6.0±2.0)\cdot10^{6}$	527	dynamic
	**DMPC**	21.0±2.0	$(4.0±0.8)\cdot10^{7}$	480	dynamic
	**DPPC**	3.2±0.9	$(6.8±2.6)\cdot10^{6}$	470	dynamic
	**DOPG**	2.0±0.2	$(3.0±0.7)\cdot10^{6}$	588	static
	**DMPG**	0.4±0.3	$(1.5±0.6)\cdot10^{6}$	533	dynamic
	**DPPG**	3.8±0.4	$(6.7±1.4)\cdot10^{6}$	564	dynamic
**ii)**	**DOPC**	$(1.2±0.2)\cdot10^{-2}$	$(2.2±0.5)\cdot10^{4}$	527	static + dynamic
	**DMPC**	$(2.0±1.0)\cdot10^{-2}$	$(4.2±2.0)\cdot10^{4}$	480	dynamic
	**DPPC**	$(1.0±0.3)\cdot10^{-2}$	$(2.0±0.9)\cdot10^{4}$	475	dynamic
	**DOPG**	$(1.1±0.4)\cdot10^{-2}$	$(2.0±0.9)\cdot10^{4}$	572	dynamic
	**DMPG**	$(2.5±0.4)\cdot10^{-2}$	$(4.0±1.0)\cdot10^{4}$	533	dynamic
	**DPPG**	$(9.2±1.6)\cdot10^{-2}$	$(2.0±0.5)\cdot10^{5}$	530	static
**iii)**	**DOPC**	$(1.8±0.1)\cdot10^{-2}$	$(4.0±0.8)\cdot10^{4}$	427	static + dynamic
	**DMPC**	$(2.8±1.6)\cdot10^{-2}$	$(1.5±1.0)\cdot10^{5}$	197	static + dynamic
	**DPPC**	$(0.9±0.2)\cdot10^{-2}$	$(3.0±0.3)\cdot10^{4}$	183	dynamic
	**DOPG**	$(2.1±0.4)\cdot10^{-2}$	$(4.0±1.0)\cdot10^{4}$	546	static + dynamic
	**DMPG**	$(3.1±1.2)\cdot10^{-2}$	$(7.0±3.0)\cdot10^{4}$	517	dynamic
	**DPPG**	$(11.0±3.0)\cdot10^{-2}$	$(2.0±0.8)\cdot10^{5}$	445	static

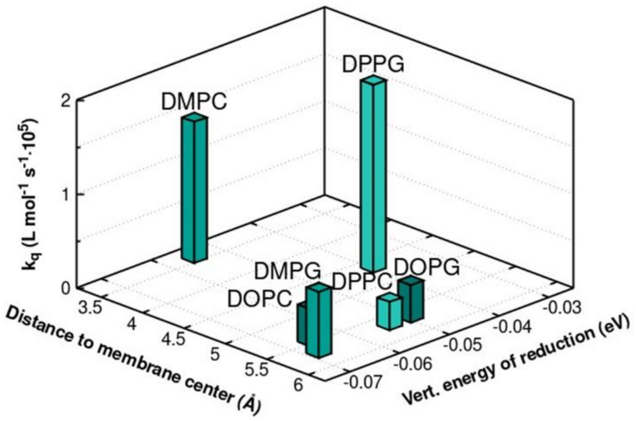

Figure S25. *3D diagrams depicting the overall trend between neutral (DOPC, DMPC and DPPC) and negative (DOPG, DMPG and DPPG) lipids, with the following parameters: Distance to membrane center, vertical reduction energy and quenching rate constants.*

*The following graphs show the photoinduced charge transfer in liposomes samples, where I_0 and τ_0 are the values in absence of quencher (sodium ascorbate or catalyst). Emission intensity, normalized lifetimes measurements and Stern-Volmer plots as a function of quencher concentration, respectively from DOPC, DMPC, DPPC, DOPG, DMPG and DPPG liposomes samples, with a composition of 100:1:2:0.2 of main lipid:(14:0 PEG2000 PE):**RuC_9** or 100:1:2:X of main lipid:(14:0 PEG2000 PE):**RuC_9:CoTTP** (main lipid = 0.3 mM), prepared in CO_2-saturated 0.1 M bicarbonate solution.*

DOPC

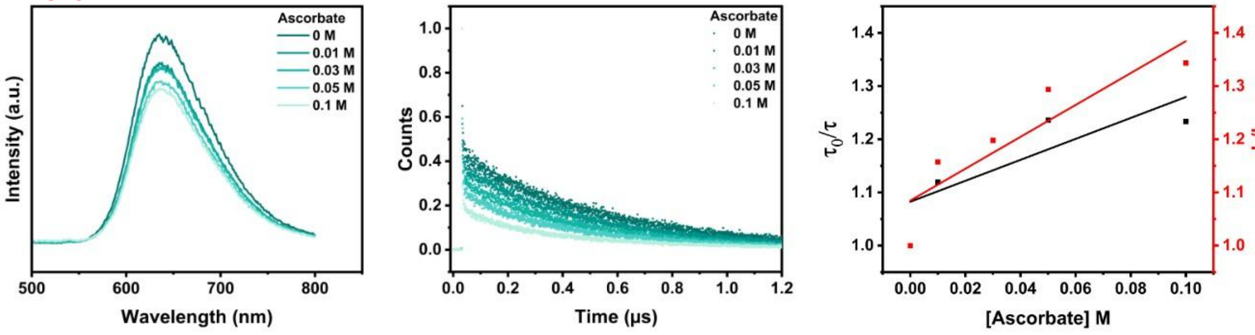

DMPC

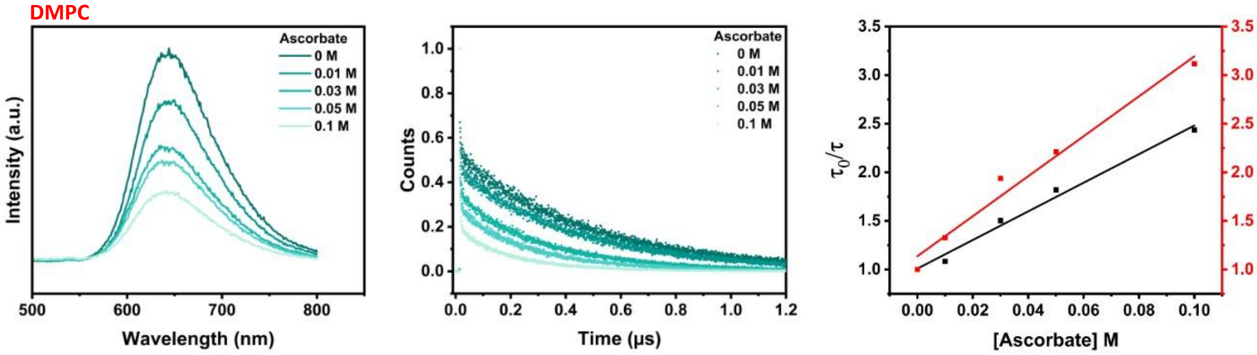

DPPC

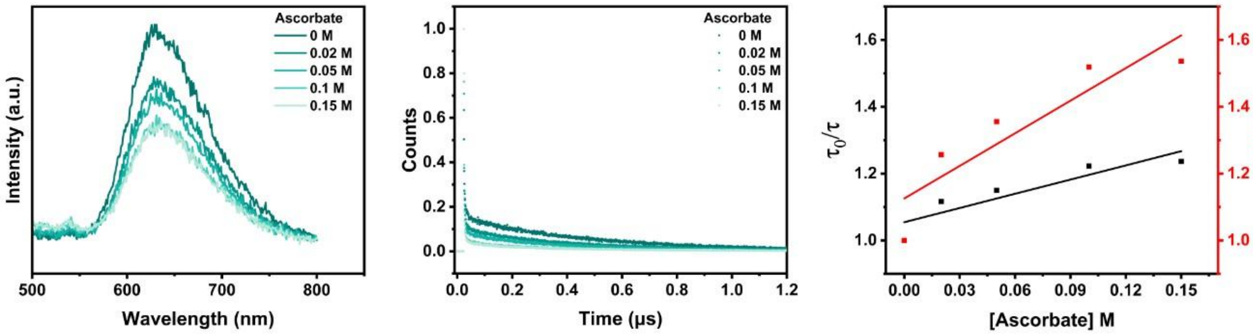

DOPG

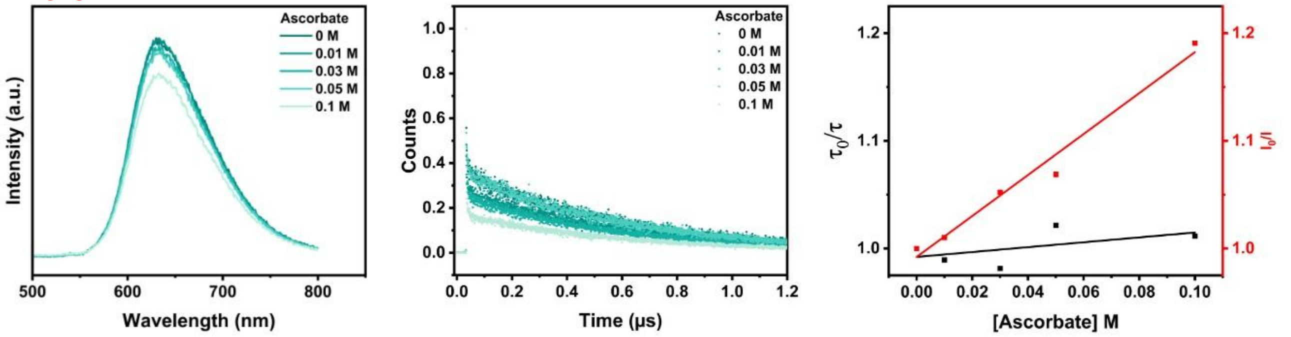

DMPG

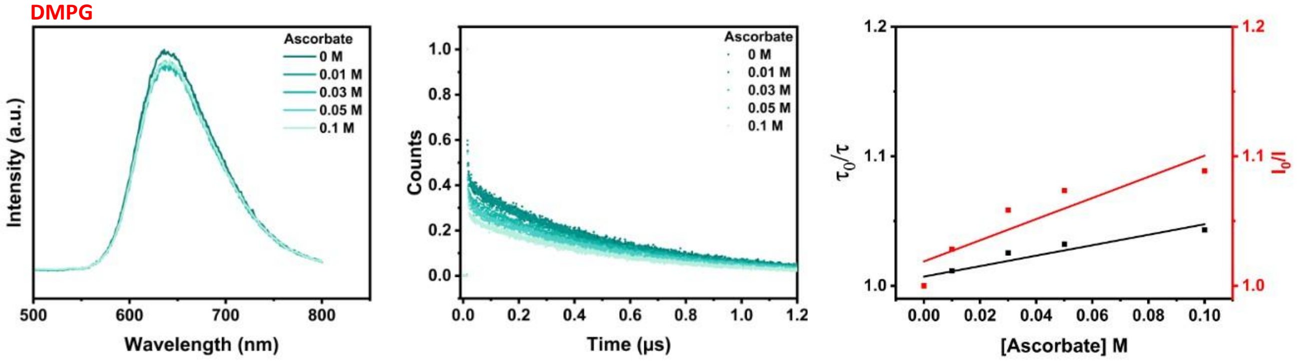

DPPG

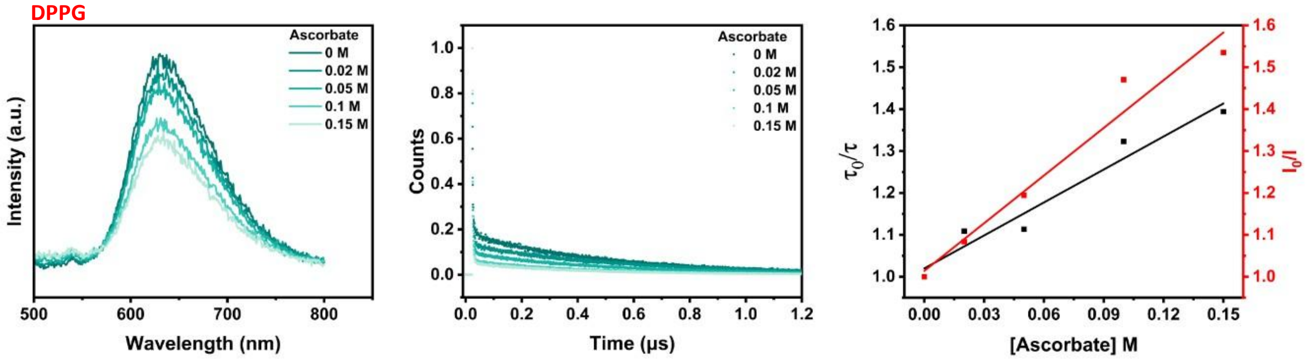

DOPC

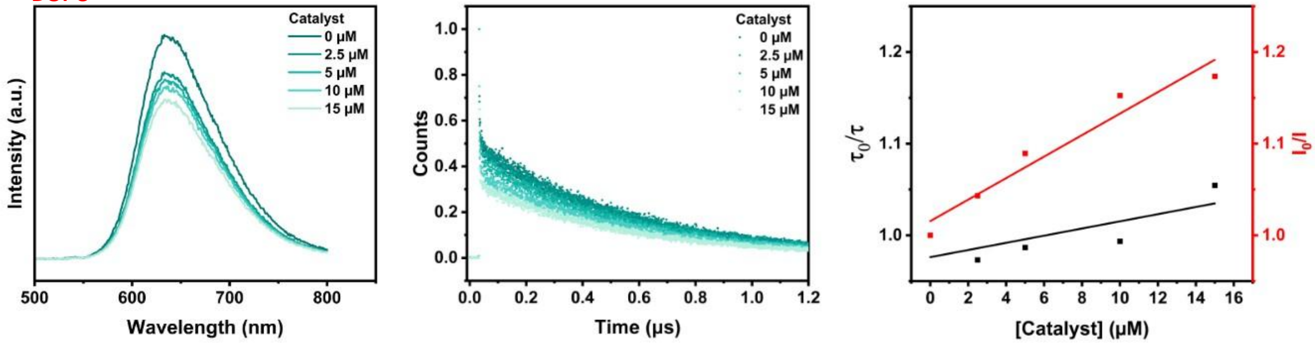

DMPC

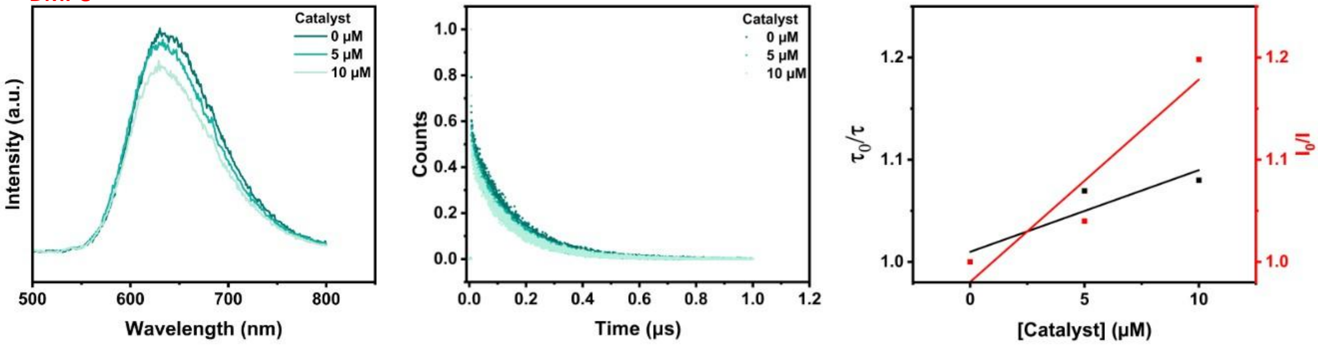

DPPC

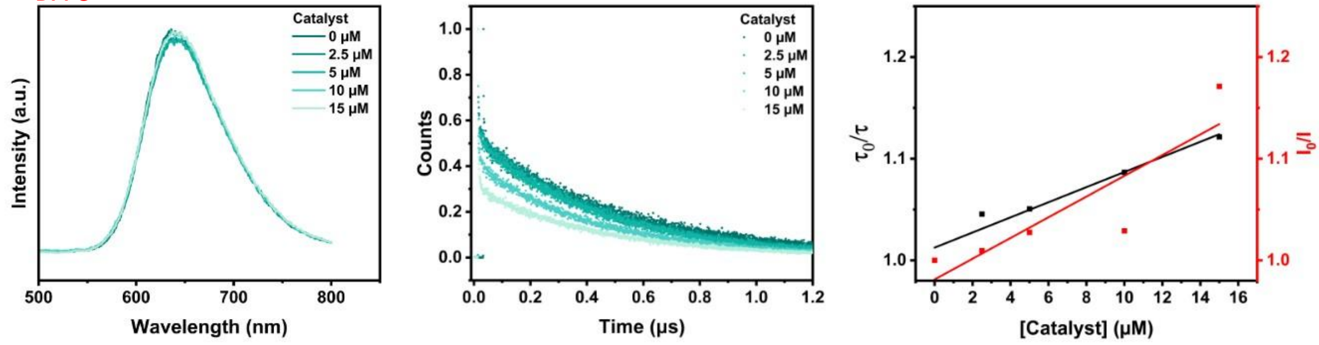

DOPG

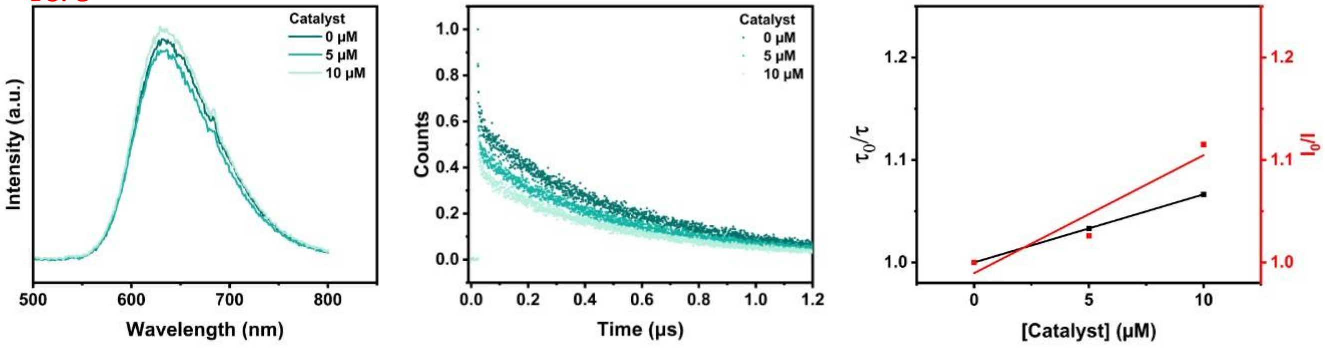

DMPG

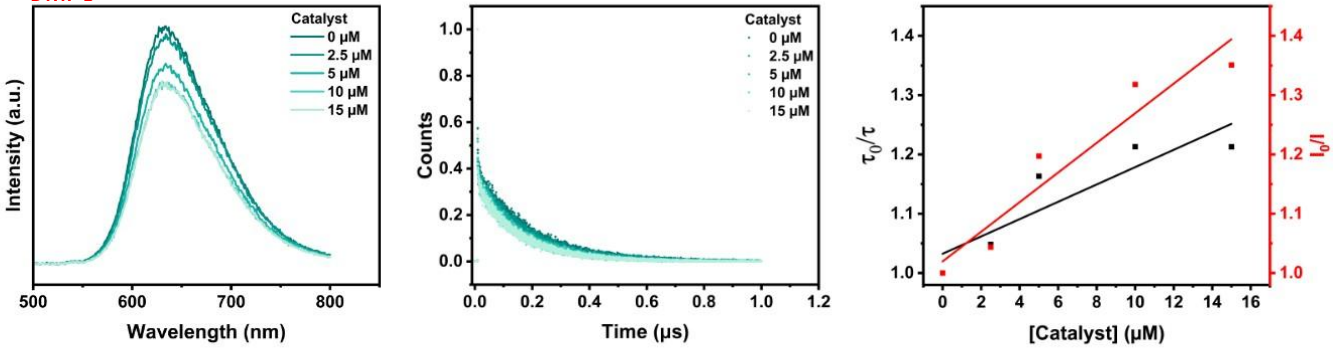

DPPG

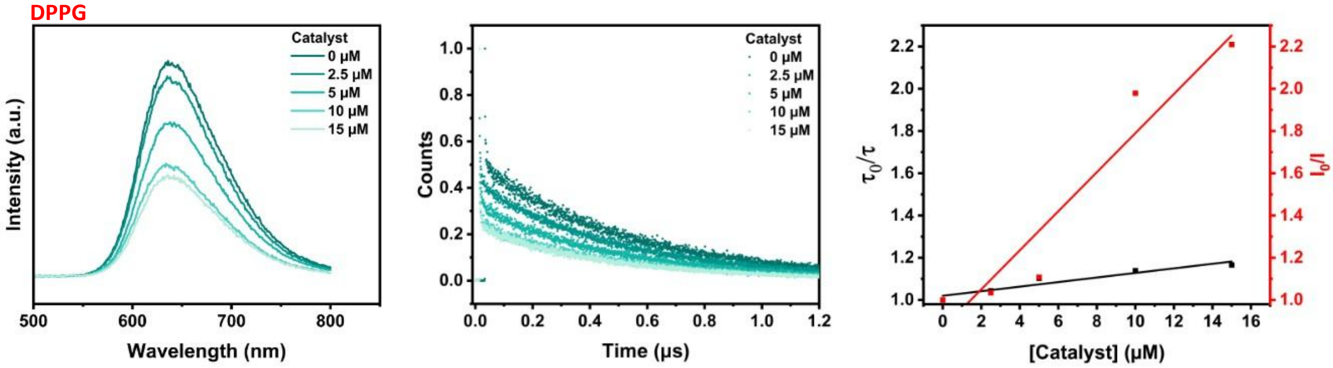

DOPC

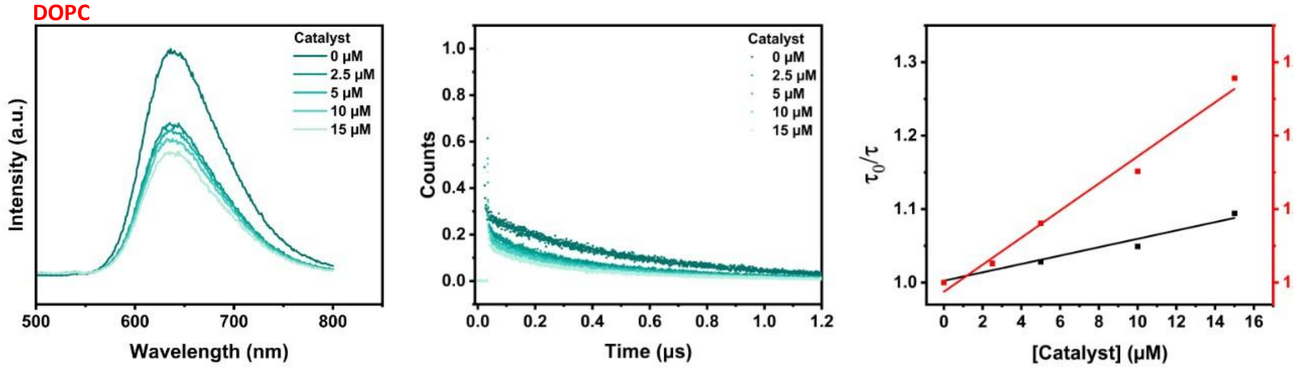

DMPC

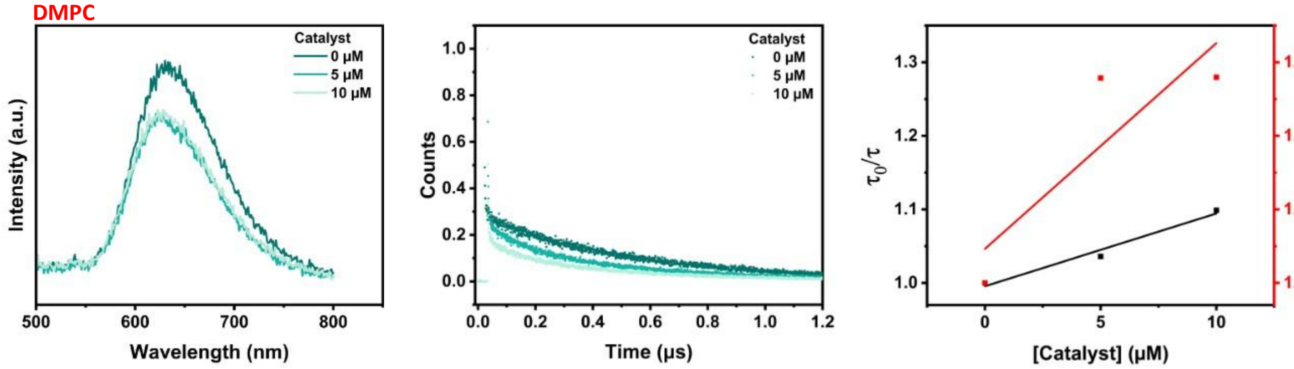

DPPC

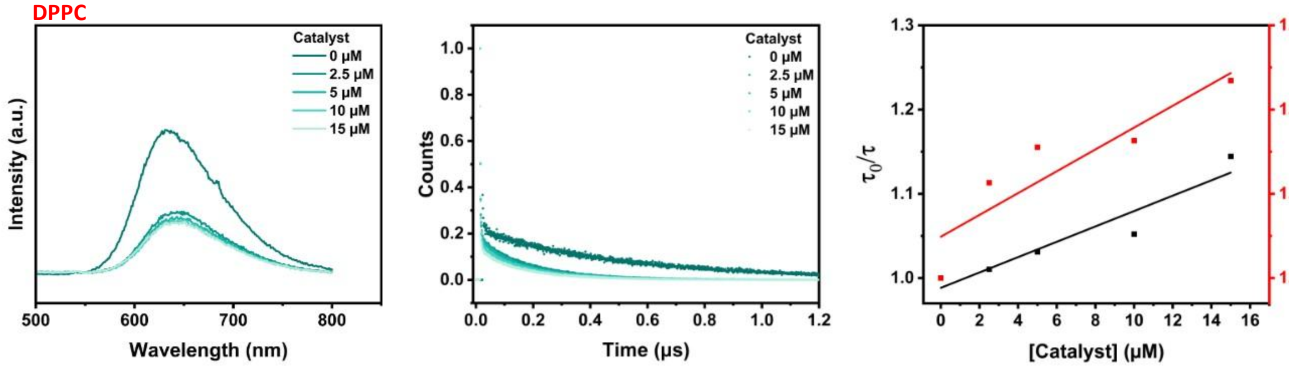

DOPG

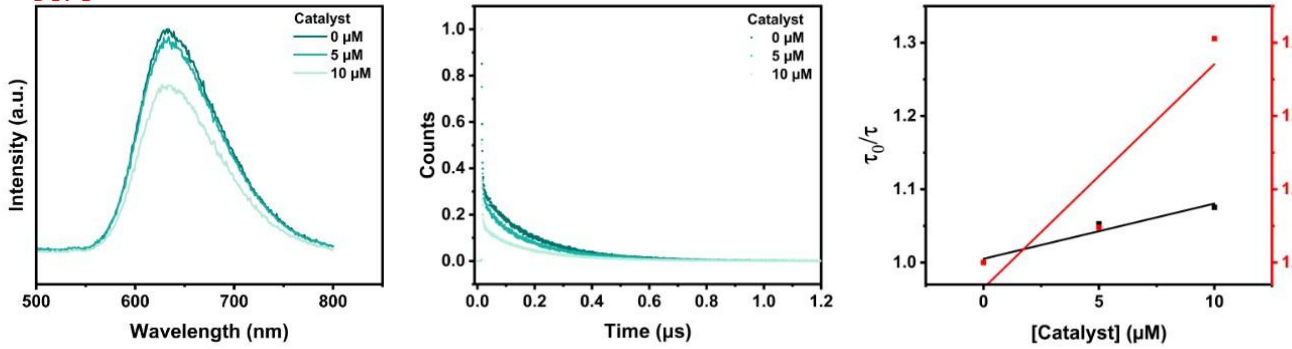

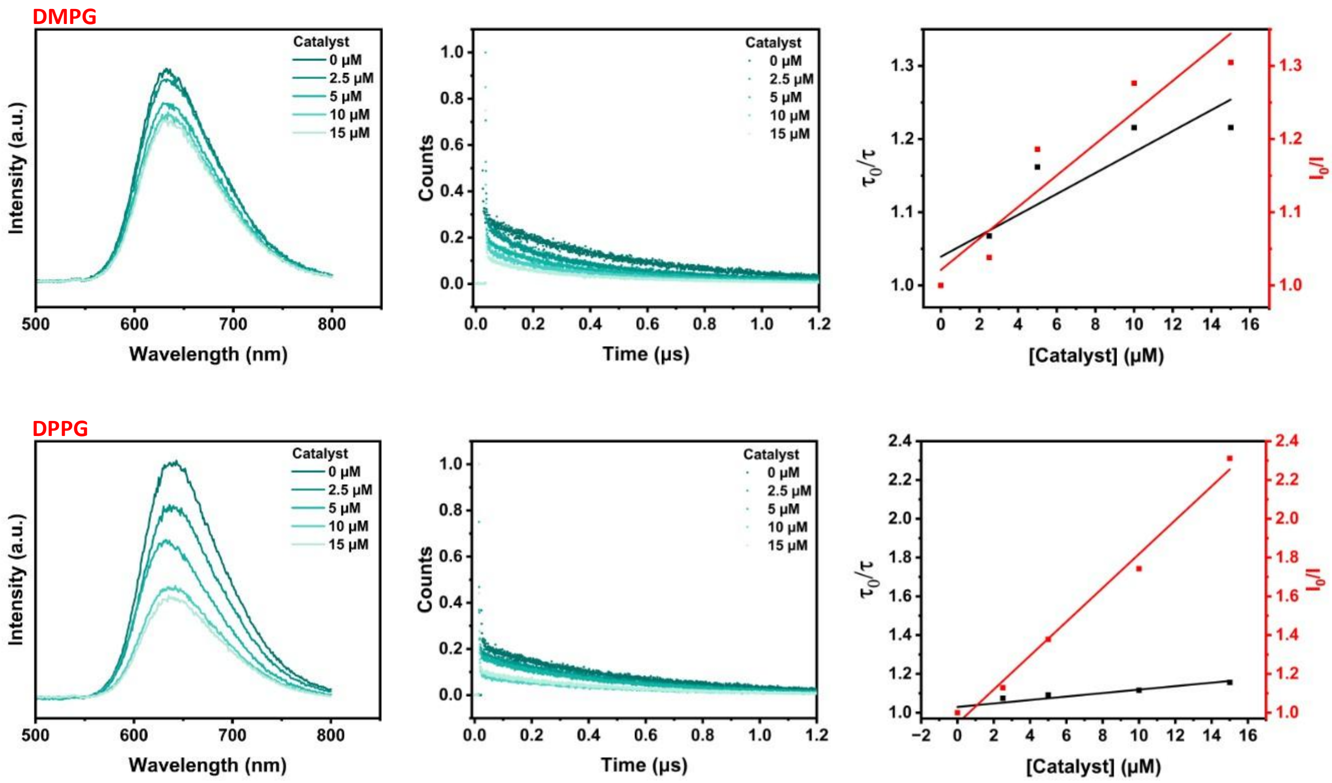

References

(1) Nau, R. E. P.; Bösking, J.; Pannwitz, A. Compartmentalization Accelerates Photosensitized NADH to NAD+ Conversion. *ChemPhotoChem* **2022**, *6* (11), e202200158. https://doi.org/10.1002/CPTC.202200158.

(2) Jacobi, R.; Hernandez-Castillo, D.; Sinambela, N.; Bosking, J.; Pannwitz, A.; Gonzalez, L. Computation of Förster Resonance Energy Transfer in Lipid Bilayer Membranes. *J. Phys. Chem. A* **2022**, *126* (43), 8070–8081. https://doi.org/10.1021/acs.jpca.2c04524.

(3) H.-M. Shen.; M.-Y. Hu; L. Liu; B. Qi; H.-L. Lie; Y.-B. She. Efficient and Selective Oxidation of Tertiary Benzylic CH Bonds with O2 Catalyzed by Metalloporphyrins under Mild and Solvent-Free Conditions. *Appl. Catal. A: Gen.* **2020**, *599*, 117599. https://doi.org/10.1016/j.apcata.2020.117599.

(4) Chatterjee, T.; Boutin, E.; Robert, M. Manifesto for the Routine Use of NMR for the Liquid Product Analysis of Aqueous CO2 Reduction: From Comprehensive Chemical Shift Data to Formaldehyde Quantification in Water. *Dalton Trans.* **2020**, *49* (14), 4257–4265. https://doi.org/10.1039/C9DT04749B.

(5) Tajmir-Riahi, H. A. Coordination Chemistry of Vitamin C. Part I. Interaction of L-Ascorbic Acid with Alkaline Earth Metal Ions in the Crystalline Solid and Aqueous Solution. *J. Inorg. Biochem.* **1990**, *40* (2), 181–188. https://doi.org/10.1016/0162-0134(90)80051-X.

(6) Seravalli, J.; Ragsdale, S. W. 13C NMR Characterization of an Exchange Reaction between CO and CO2 Catalyzed by Carbon Monoxide Dehydrogenase†. *Biochemistry* **2008**, *47* (26), 6770–6781. https://doi.org/10.1021/BI8004522.

(7) Bonhomme, F.; Alam, T. M.; Celestian, A. J.; Tallant, D. R.; Boyle, T. J.; Cherry, B. R.; Tissot, R. G.; Rodriguez, M. A.; Parise, J. B.; Nyman, M. Tribasic Lead Maleate and Lead Maleate: Synthesis and Structural and Spectroscopic Characterizations. *Inorg. Chem.* **2005**, *44* (21), 7394–7402. https://doi.org/10.1021/ic050611y.

(8) Komber, H. The 1H and 13C NMR Spectra of an Alternating Ethene/Maleic Anhydride Copolymer and the Corresponding Acid and Sodium Salt. *Macromol. Chem. Phys.* **1995**, *196* (2), 669–678. https://doi.org/10.1002/macp.1995.021960222.

(9) Ilczyszyn, M.; Godzisz, D.; Ilczyszyn, M. M. Sarcosine–Maleic Acid (1:1) Crystal: Structure, 13C NMR and Vibrational Properties, Protonation Character. *Spectrochimica Acta Part A* **2003**, *59* (8), 1815–1828. https://doi.org/10.1016/S1386-1425(02)00413-4.

(10) Moret, S.; Dyson, P. J.; Laurenczy, G. Direct, in Situ Determination of PH and Solute Concentrations in Formic Acid Dehydrogenation and CO2 Hydrogenation in Pressurised Aqueous Solutions Using 1H and 13C NMR Spectroscopy. *Dalton Trans.* **2013**, *42* (13), 4353–4356. https://doi.org/10.1039/C3DT00081H.

(11) Jo, S.; Kim, T.; Iyer, V. G.; Im, W. CHARMM-GUI: A Web-Based Graphical User Interface for CHARMM. *J Comput Chem* **2008**, *29* (11), 1859–1865. https://doi.org/10.1002/JCC.20945.

(12) Wu, E. L.; Cheng, X.; Jo, S.; Rui, H.; Song, K. C.; Dávila-Contreras, E. M.; Qi, Y.; Lee, J.; Monje-Galvan, V.; Venable, R. M.; Klauda, J. B.; Im, W. CHARMM-GUI Membrane Builder toward Realistic Biological Membrane Simulations. *J Comput Chem* **2014**, *35* (27), 1997–2004. https://doi.org/10.1002/JCC.23702.

(13) Kučerka, N.; Tristram-Nagle, S.; Nagle, J. F. Structure of Fully Hydrated Fluid Phase Lipid Bilayers with Monounsaturated Chains. *J. Membr. Biol* **2005**, *208* (3), 193–202. https://doi.org/10.1007/S00232-005-7006-8.

(14) Case, D.; Aktulga, H. M.; Belfon, K.; Ben-Shalom, I.; Berryman, J.; Brozell, S.; Cerutti, D.; Cheatham, T.; Cisneros, G. A.; Cruzeiro, V.; Darden, T.; Duke, R.; Giambasu, G.; Gilson, M.; Gohlke, H.; Götz, A.; Harris, R.; Izadi, S.; Izmailov, S.; et al. *Amber 2022*; University of California: San Francisco, CA, USA, 2022. https://doi.org/10.13140/RG.2.2.31337.77924.

(15) Salomon-Ferrer, R.; Götz, A. W.; Poole, D.; Le Grand, S.; Walker, R. C. Routine Microsecond Molecular Dynamics Simulations with AMBER on GPUs. 2. Explicit Solvent Particle Mesh Ewald. *J. Chem. Theory Comput.* **2013**, *9* (9), 3878–3888. https://doi.org/doi/full/10.1021/ct400314y.

(16) Götz, A. W.; Williamson, M. J.; Xu, D.; Poole, D.; Le Grand, S.; Walker, R. C. Routine Microsecond Molecular Dynamics Simulations with AMBER on GPUs. 1. Generalized Born. *J. Chem. Theory Comput.* **2012**, *8* (5), 1542–1555. https://doi.org/doi/full/10.1021/ct200909j.

(17) Le Grand, S.; Götz, A. W.; Walker, R. C. SPFP: Speed without Compromise—A Mixed Precision Model for GPU Accelerated Molecular Dynamics Simulations. *Comput. Phys. Commun.* **2013**, *184* (2), 374–380. https://doi.org/10.1016/j.cpc.2012.09.022.

(18) Wang, J.; Wolf, R. M.; Caldwell, J. W.; Kollman, P. A.; Case, D. A. Development and Testing of a General Amber Force Field. *J. Comput. Chem.* **2004**, *25* (9), 1157–1174. https://doi.org/10.1002/JCC.20035.

(19) Shahrokh, K.; Orendt, A.; Yost, G. S.; Cheatham, T. E. Quantum Mechanically Derived AMBER-Compatible Heme Parameters for Various States of the Cytochrome P450 Catalytic Cycle. *J. Comput. Chem.* **2012**, *33* (2), 119–133. https://doi.org/10.1002/JCC.21922.

(20) Chen, X.; Hu, X. M.; Daasbjerg, K.; Ahlquist, M. S. G. Understanding the Enhanced Catalytic CO2 Reduction upon Adhering Cobalt Porphyrin to Carbon Nanotubes and the Inverse Loading Effect. *Organometallics* **2020**, *39* (9), 1634–1641. https://doi.org/10.1021/acs.organomet.9b00726.

(21) Becke, A. D. Density-functional Thermochemistry. III. The Role of Exact Exchange. *J. Chem. Phys.* **1993**, *98* (7), 5648–5652. https://doi.org/10.1063/1.464913.

(22) Stephens, P. J.; Devlin, F. J.; Chabalowski, C. F.; Frisch, M. J. Ab Initio Calculation of Vibrational Absorption and Circular Dichroism Spectra Using Density Functional Force Fields. *J. Phys. Chem.* **1994**, *98* (45), 11623–11627. https://doi.org/10.1021/j100096a001.

(23) Weigend, F.; Ahlrichs, R. Balanced Basis Sets of Split Valence, Triple Zeta Valence and Quadruple Zeta Valence Quality for H to Rn: Design and Assessment of Accuracy. *Phys. Chem. Chem. Phys.* **2005**, *7* (18), 3297–3305. https://doi.org/10.1039/B508541A.

(24) Weigend, F. Accurate Coulomb-Fitting Basis Sets for H to Rn. *Phys. Chem. Chem. Phys.* **2006**, *8* (9), 1057–1065. https://doi.org/10.1039/B515623H.

(25) Grimme, S.; Ehrlich, S.; Goerigk, L. Effect of the Damping Function in Dispersion Corrected Density Functional Theory. *J. Comput. Chem.* **2011**, *32* (7), 1456–1465. https://doi.org/10.1002/JCC.21759.

(26) Breneman, C. M.; Wiberg, K. B. Determining Atom-Centered Monopoles from Molecular Electrostatic Potentials. The Need for High Sampling Density in Formamide Conformational Analysis. *J. Comput. Chem.* **1990**, *11* (3), 361–373. https://doi.org/10.1002/JCC.540110311.

(27) Barone, V.; Cossi, M. Quantum Calculation of Molecular Energies and Energy Gradients in Solution by a Conductor Solvent Model. *J. Phys. Chem. A* **1998**, *102* (11), 1995–2001. https://doi.org/10.1021/jp9716997.

(28) Cossi, M.; Rega, N.; Scalmani, G.; Barone, V. Energies, Structures, and Electronic Properties of Molecules in Solution with the C-PCM Solvation Model. *J. Comput. Chem.* **2003**, *24* (6), 669–681. https://doi.org/10.1002/JCC.10189.

(29) Frisch, M. J.; Trucks, G. W.; Schlegel, H. B.; Scuseria, G. E.; Robb, M. A.; Cheeseman, J. R.; Scalmani, G.; Barone, V.; Petersson, G. A.; Nakatsuji, H.; et al. Gaussian16 , Rev. C.01. *Gaussian16, Rev. C.01* **2016**.

(30) Dickson, C. J.; Walker, R. C.; Gould, I. R. Lipid21: Complex Lipid Membrane Simulations with AMBER. *J. Chem. Theory Comput.* **2022**, *18* (3), 1726–1736. https://doi.org/10.1021/acs.jctc.1c01217.

(31) Izadi, S.; Anandakrishnan, R.; Onufriev, A. V. Building Water Models: A Different Approach. *J. Phys. Chem. Lett.* **2014**, *5* (21), 3863–3871. https://doi.org/10.1021/jz501780a.

(32) Sengupta, A.; Li, Z.; Song, L. F.; Li, P.; Merz, K. M. Parameterization of Monovalent Ions for the OPC3, OPC, TIP3P-FB, and TIP4P-FB Water Models. *J. Chem. Inf. Model.* **2021**, *61* (2), 869–880. https://doi.org/10.1021/acs.jcim.0c01390.

(33) Ryckaert, J. P.; Ciccotti, G.; Berendsen, H. J. C. Numerical Integration of the Cartesian Equations of Motion of a System with Constraints: Molecular Dynamics of n-Alkanes. *J. Comput. Phys.* **1977**, *23* (3), 327–341. https://doi.org/10.1016/0021-9991(77)90098-5.

(34) Miyamoto, S.; Kollman, P. A. Settle: An Analytical Version of the SHAKE and RATTLE Algorithm for Rigid Water Models. *J. Comput. Chem.* **1992**, *13* (8), 952–962. https://doi.org/10.1002/JCC.540130805.

(35) Roe, D. R.; Cheatham, T. E. PTRAJ and CPPTRAJ: Software for Processing and Analysis of Molecular Dynamics Trajectory Data. *J. Chem. Theory Comput.* **2013**, *9* (7), 3084–3095. https://doi.org/10.1021/ct400341p.
